# Coevolution analysis of *Hepatitis C* virus genome to identify the structural and functional dependency network of viral proteins

**DOI:** 10.1038/srep26401

**Published:** 2016-05-20

**Authors:** Raphaël Champeimont, Elodie Laine, Shuang-Wei Hu, Francois Penin, Alessandra Carbone

**Affiliations:** 1 Sorbonne Universités, UPMC-Univ P6, CNRS, Laboratoire de Biologie Computationnelle et Quantitative - UMR 7238, 15 rue de l’Ecole de Médecine, 75006 Paris, France; 2 CNRS, UMR5086, Bases Moléculaires et Structurales des Systèmes Infectieux, Institut de Biologie et Chimie des Protéines, 7 Passage du Vercors, Cedex 07, F-69367 Lyon, France; 3 LABEX Ecofect, Université de Lyon, Lyon, France; 4 Institut Universitaire de France, 75005, Paris, France

## Abstract

A novel computational approach of coevolution analysis allowed us to reconstruct the protein-protein interaction network of the Hepatitis C Virus (HCV) at the residue resolution. For the first time, coevolution analysis of an entire viral genome was realized, based on a limited set of protein sequences with high sequence identity within genotypes. The identified coevolving residues constitute highly relevant predictions of protein-protein interactions for further experimental identification of HCV protein complexes. The method can be used to analyse other viral genomes and to predict the associated protein interaction networks.

Protein-protein interactions may involve two or more partners. The molecular mechanisms underlying these interactions and their implication for the regulation of biological processes might be multiple. Their analyses are difficult^1^,^2^,^3^,^4^,^5^,^6^,^7^,^8^,^9^,^10^, not only because to detect their existence is intrinsically complicated^10^,^11^,^12^,^13^,^14^,^15^,^16^,^17^,^18^,^19^,^20^,^21^,^22^,^23^, but also because several interactions might involve the same residues and to discriminate the different roles of a residue in different interactions^9^,^23^,^24^,^25^ remains nowadays a challenge. This means that computational tools helping to unravel such information are most welcome.

A particular focus has been drawn in recent years to coevolving residues, within a protein and among proteins. Coevolving residues in a protein structure, possibly a complex, correspond to groups of residues whose mutations have arisen simultaneously during the evolution of different species, and this is due to several possible reasons involving the three-dimensional shape of the protein: functional interactions, conformational changes and folding. Several studies addressed the problem of extracting signals of coevolution between residues. All these methods provide sets of coevolved residues that are usually close in the three-dimensional structure^26^,^27^,^28^,^29^,^30^,^31^,^32^,^33^, form connected networks covering roughly a third of the entire structure, and have been demonstrated for a few protein complexes (for which experimental data was available) to play a crucial role in allosteric mechanisms^26^,^28^,^34^, to maintain short paths in network communication and to mediate signaling^35^,^36^. For an overview of the many methods for coevolution analysis developed in recent years, see^37^. These methods are applicable to protein families displaying a large number of evolutionarily related sequences and sufficient divergence, these characteristics constituting the bottleneck of today coevolution analysis methods^30^,^38^,^39^. Several studies pointed out that a correct theoretical framework of molecular coevolution would strongly help to assess the evolutionary origin of the signals observed^40^,^41^,^42^,^43^,^44^.

For many proteins, characteristic of vertebrate or viral species, coevolution methods are not applicable because of the reduced number of sequences (either coming from species or from populations) and their conservation. Statistical approaches that estimate the “background noise” in these sequences cannot be applied and alternative paradigms should be followed. To overcome these difficulties, we developed a fast algorithm for the coevolution analysis of relatively small sets of sequences (where “small” means <50 sequences) displaying high similarity, called BIS^2^. BIS^2^ is a new computationally efficient version of Blocks In Sequences (BIS)^45^, a coevolution analysis method that could successfully handle highly conserved proteins such as the Amyloid beta peptide for Alzheimer’s disease and families of very few sequences such as the ATPase protein families. These studies highlighted that coevolving protein fragments are indicators of important information explaining folding intermediates, peptide assembly, key mutations with known roles in genetic diseases, distinguished subfamily-dependent motifs^45^. They could capture, with high precision, experimentally verified hotspots residues^45^. The BIS method demonstrated to go beyond the bottleneck of analysis present in current coevolution studies and its improved performance in the present study allows us to realise a complete coevolution analysis of the small Hepatitis C Virus (HCV) genome of 10 proteins, opening the way to coevolution studies of protein-protein interaction networks in viral genomes.

Coevolution of proteins has to comply with multiple protein interactions^46^,^47^ as well as avoid a huge amount of potential interactions with non-partners^48^,^49^. Such non-partners might be proteins entering in competition, but also molecules, such as DNA, RNA, small peptides. In previous studies^49^, it was shown that inhibitors, enzymes, antibodies and antigens evolved to avoid the interaction among proteins of the same class. Viral genomes like that of HCV, coding for a dozen proteins, form less complex interacting systems compared to genomes hosting hundreds or thousands of genes. Consequently, they appear as good starting points for investigating the feasibility of coevolution studies to reconstruct protein-protein networks.

Our goal is to identify the interaction network between HCV proteins and to describe, at the residue level, how these interactions take place, by identifying the amino acids that are involved. We have chosen to work with HCV for several reasons. On the one hand, it is because of its obvious medical interest: HCV infection is a leading cause of chronic hepatitis, liver cirrhosis and hepatocellular carcinoma worldwide^50^. On the other hand, HCV has a relatively simple genome. Indeed, HCV contains a 9.6-kb positive-strand RNA genome encoding a single polyprotein precursor that is processed by cellular and viral proteases into ten mature proteins (reviewed in^51^; see Fig. 1A). HCV genes do not overlap, in contrast to other viral genomes, and their independent gene encoding reduces the evolutionary constraints that the genome could undergo at the nucleotide level. The structural proteins, which form the viral particle, include the core protein and the envelope glycoproteins E1 and E2. The nonstructural proteins include the p7 viroporin, the NS2 protease, the NS3-4A complex harboring protease and NTPase/RNA helicase activities, the NS4B and NS5A proteins, and the NS5B RNA-dependent RNA polymerase. Great progress has been made over the past years in elucidating the structure and function of these proteins, most of which are still actively being pursued as antiviral targets^52^. (See Fig. 1B)

In contrast, the molecular mechanisms of HCV replication remain largely unknown. In particular, although the overall interaction network between HCV proteins has been studied by various approaches^53^,^54^ (indexed in HCVpro database^55^), little is known about the detailed physical interactions between these proteins. We used coevolution analysis of protein residues to address this question on the full HCV polyprotein, that is, on the 10 HCV proteins considered at once. An analogous computational experiment was never realised before for two reasons. First, we look at genotype sequences, that is sequences that are evolutionarily very close to each other, and therefore very conserved, while existing coevolution analysis methodologies treat sets of divergent sequences and their application to sets of conserved sequences is impossible. The second reason is that the number of available sequences is limited here, varying from 24 to 40, while available coevolution analysis tools demand at least 100 sequences and in certain cases, several thousands^26^,^27^,^28^,^29^,^30^,^32^,^38^,^39^.

Our computational analysis reveals a complex network of interactions between the different proteins coded by the HCV genome. The description that it provides is finer than the one encoded in a classical protein-protein interaction network which only reports whether interactions between pairs of proteins do exist (presence of an edge) or do not exist (absence of an edge). The reason is twofold: 1. we identified the protein domain-domain interaction network, highlighting what are the residues and the domains that play a role in the interactions with other proteins, 2. we constructed the network from correlations involving multiple proteins instead of using information involving pairs of proteins. Importantly, the corresponding database of coevolving residues constitute a set of highly relevant predictions of protein-protein interactions for further experimental identification of HCV protein complexes.

## Results

### Coevolution analysis of the HCV genome

Along protein sequences, coevolution analysis identifies residue pairs that demonstrate either a specific co-adaptation, where changes in one of the residues are compensated by changes in the other during evolution, or a less specific external force that affects the evolutionary rates of both residues in a similar magnitude. In both cases, independently of the underlying cause, co-evolutionary signatures within or between proteins serve as markers of physical interactions and/or functional relationships.

The coevolution analysis method BIS^45^, and its new implementation BIS^2^, start from a coevolution analysis of a pool of aligned protein sequences, provides a score of coevolution for each pair of positions in the sequence alignment, and clusters together those positions that display similar scores of coevolution with all other positions in the alignment (Fig. 2A–C). The clustering step allows to group together those residues that exhibit co-evolution during sequence evolution. BIS^2^ can be applied on a single protein, on a pair of proteins and also on multiple proteins at once as it is the case here for the HCV polyprotein.

In our HCV analysis, we only consider “perfect patterns” of coevolution, that is pairs of positions in the alignment described by distribution of residues where a change in a position appears simultaneously as a change in the other position. In other words, there is a perfect bijection between amino acids within the first and the second position. Formally, this means that for any two pairs of amino acids *a*_1_, *b*_1_ and *a*_2_, *b*_2_ that one reads on two different aligned sequences at positions *p*_1_, *p*_2_, we have 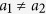 if and only if 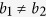. BIS^2^ associates a maximum coevolution score to such patterns. In Fig. 2A, a bijection exists for positions 4 and 9, colored dark red because they reach the highest score of coevolution: in position 4, residues *SKAW* occur exactly when residues *KEGH* occur in position 9, respectively. If the same perfect pattern is observed in more than two positions, BIS^2^ identifies a cluster of positions that will be studied together. The observation of these Òperfect patternsÓ in HCV genome sequences supports the pertinence of studying HCV polyproteins through coevolution analysis of strong signals (with maximal score). The detection of weaker signals (with scores that are not maximal, e.g. the orange columns in Fig. 2A) is envisageable and it will be reported elsewhere.

BIS^2^ coevolution analysis provides a large number of clusters of residues that are filtered to retain only those that are statistically significant. This means that we retain only clusters with a perfect coevolution pattern, as illustrated in Fig. 2A–C and that are not fully conserved (see Methods). For each cluster, statistical tests provide a p-value that allows to control the False Discovery Rate (FDR; see Methods). For simplicity, even though clusters are computed as sets of alignment positions, we refer to them as clusters of residues, referring to the residues associated to the sequence alignment.

It is important to notice that HCV genotype sequences are very conserved and that BIS^2^ identifies perfect patterns only among the few positions in the alignment that are not fully conserved. In practice, this means that for HCV sets of sequences, coevolution signals are only detectable on a quite restricted set of positions. In Fig. 2D, we report the ensemble of positions where BIS^2^ analysis of the NS5A protein can be applied to, for three different HCV genotype datasets. For each genotype, one observes a relatively small set of non fully conserved residues that, in many cases, are not in contact with other non fully conserved residues.

### From interaction links to a network

Coevolution among residues in a cluster can be due to direct interactions, corresponding to physical contacts, or to indirect interactions, corresponding to dependency relations as allostericity, or signalling. Both kinds of links between residues can be observed within a protein (“intra”) and between proteins (“inter”).

Clusters of residues are intended to highlight the residues in a structure that are crucial to the protein functional activity, structural stability and/or interaction with other proteins. In particular, one should not expect that all residues in a cluster establish direct contacts with each other. Namely, there are three possible ways that a single cluster can stand for the interaction of distinct proteins *P*_1_, *P*_2_, *P*_3_ by containing residues from *P*_1_, *P*_2_ and *P*_3_. These ways are illustrated in Fig. 3A:

1. *P*_1_ can interact with *P*_2_ and *P*_3_ through the same physical contacts but in different spatio-temporal contexts; this implies a direct interaction for *P*_1_, *P*_2_ and *P*_1_, *P*_3_ but an indirect coevolution link between *P*_2_, *P*_3_ (Fig. 3A, left).
2. *P*_1_ can interact with *P*_2_ and *P*_3_ through different physical contacts, and this implies a direct interaction for *P*_1_, *P*_2_ and *P*_1_, *P*_3_, and an indirect coevolution link between *P*_2_, *P*_3_. It also implies indirect intra coevolution links involving residues in the two interactions of *P*_1_ (Fig. 3A, center).
3. A complex comprising the three proteins is formed and the physical interactions are distinct for the three direct interactions involving *P*_1_, *P*_2_, *P*_1_, *P*_3_ and *P*_2_, *P*_3_; this asks for indirect intra coevolution links within *P*_1_, *P*_2_ and *P*_3_ (Fig. 3A, right).

In Fig. 3B, we illustrate the graph-like representation of the interactions between *P*_1_, *P*_2_ and *P*_3_ described in points 1–3, when their residues belong to a same cluster. The three conditions correspond to the formation of a “three-edges cycle” in the graph.

If two distinct clusters stand for the interactions of proteins *P*_1_, *P*_2_, *P*_3_, the resulting graph can be different from the one in Fig. 3B. In fact, if the two interactions described by the first two schema in Fig. 3A are represented by different clusters as illustrated in Fig. 3C, then the graph-like representation of the interactions is a “branching motif” as illustrated in Fig. 3D. However, if the three interactions described in the right-hand-side schema of Fig. 3A are represented by different clusters (Fig. 3E), then the corresponding graph of interaction is as in Fig. 3B.

In the sequel, we shall build up graphs of interaction by superimposing three-edges cycles and branching motifs corresponding to interactions among more than 3 proteins, as illustrated in Fig. 4 (bottom). In this figure, selected examples of clusters of co-evolving residues are shown along the HCV polyprotein represented as a strip, and their associated graph is also reported. (See Supplementary Figures 1–3 for visualisation and details of all predicted clusters.).

Given a cluster, we shall consider all the links between those proteins whose residue positions are involved in the cluster. This means that the associated graph of interactions will represent the ensemble of all observed links and it will display the amount of observed coevolving residues between pairs of proteins. Formally, this is done by associating to an edge, between two proteins *P*_1_, *P*_2_, a weight defined as the sum of the correlated positions in *P*_1_ and *P*_2_, and it is indicated by the thickness of the edge. For example, if in a cluster there are 3 positions from protein *P*_1_ and 5 positions from protein *P*_2_, we count 5 + 3 positions for the *P*_1_–*P*_2_ interaction. As exemplified on the right of Fig. 4, thick edges between HCV proteins correspond to a high number of coevolving positions and they might represent a direct or an indirect interaction.

Given all clusters issued by coevolution analysis, we generate a graph of protein interactions representing how much evidence for the interaction between pairs of proteins is found in all clusters (Fig. 5A). Namely, the nodes of the graph correspond to the 10 HCV proteins and an edge between *P*_1_ and *P*_2_ exists if there is at least one cluster that contains positions from *P*_1_ and *P*_2_. The weight of an edge between *P*_1_ and *P*_2_ is computed as the sum of the weights of the edges between *P*_1_ and *P*_2_ over all graphs associated to clusters. We refer to the weight as the “strength” of the interaction link.

### Comparison with the topology of the experimental network

The graph of protein interactions issued from BIS^2^ analysis of the HCV polyprotein is illustrated in Fig. 5A in comparison to the network of reported HCV protein-protein interactions determined by using various experimental methods^54^ (Fig. 5B). Both graphs indicate the existence of an entangled protein-protein network in HCV, likely reflecting a complex dynamics in the viral life cycle. This is expected from what is already known of the dynamics of the HCV virus as discussed below.

Note that most links detected by BIS^2^ are also detected experimentally, as shown by the large proportion of black links on the graph in Fig. 5A (these links are shared with experimental ones) compared to blue links that are missing in the graph of Fig. 5B. We performed a correlative analysis between the strength of the inferred links in Fig. 5A and the presence/absence of edges from experiment in Fig. 5B. Namely, we considered the average number of coevolving residues predicted for experimentally confirmed interactions (29.59) and the average number of coevolving residues predicted for interactions that are not experimentally confirmed (13.62), and we performed a t-test (one-tailed, Welch variant, i.e. without asking for equal variances) to see if the average of the first population is greater than the average of the second one. We obtained a p-value of 0.01528 showing that there is a statistically meaningful correlation between experimentally validated interactions and the number of coevolving residues predicted for these interactions. This test confirms that the network of coevolution links (Fig. 5A) reflects biological reality (Fig. 5B).

**Figure 1:**
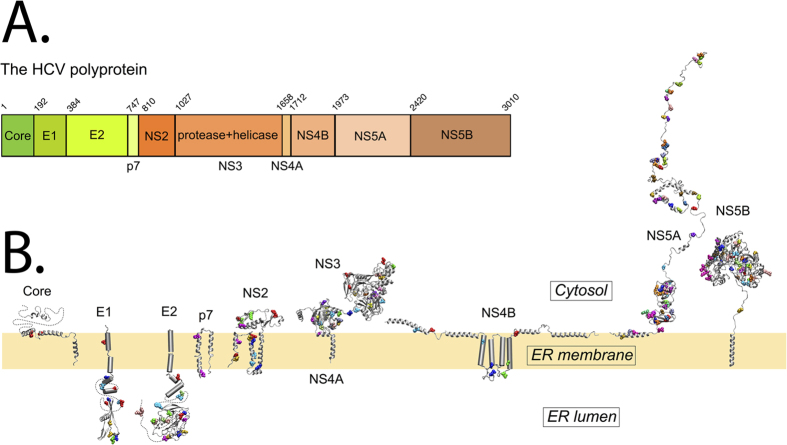
A global view of the intra protein coevolving residues in HCV proteins. (**A**) The HCV polyprotein is described as a strip where proteins are identifiable with their name and their amino acid position along the strip. The positions correspond to the residue numbers in the Con1 strain HCV polyprotein (accession number: AJ238799). (**B**) The structures of the proteins in the HCV polyprotein are localized with respect to the membrane, the cytosol and the ER lumen. Coevolution analysis was run on individual HCV proteins and only clusters that include at least two positions in the associated protein 3D structure were gathered and mapped on the structures. (See section “Mapping to structures” in Methods for the mapping between alignment and structure). For each cluster, coevolving residues are shown as spheres and colored with a different color. Notice that colors are independent from protein to protein, that is, similar colors on different proteins do not indicate them to belong to the same cluster. Known protein structures are shown as ribbon diagrams. The structures and the membrane bilayer are shown at the same scale. Proteins or protein segments of unresolved structure are represented as cylinders with their approximate sizes for helices and dotted lines for unknown structures. From left to right: core protein, E1 and E2 envelop glycoproteins, p7 viroporin (monomer model), NS2 autoprotease, NS3 serine protease domain associated to NS4A protein and linked to NS3, NTPase/RNA helices domain, NS4B integral membrane protein, NS5A regulation phosphoprotein, and NS5B RNA-dependent RNA polymerase (for detailed structural information, see section “Structures and modeling of the HCV proteins”). The membrane is schematically represented in yellow (bilayer thickness of POPC (1-palmitoyl,2-oleoyl-sn-glycero-3-phosphocholine)). The positioning of in-plane and transmembrane segments are either deduced from molecular dynamics simulations in POPC bilayer (p7, NS5A in-plane membrane helix) or tentative (all other proteins). The figure was generated from the structure coordinates deposited in the PDB using Visual Molecular Dynamics (VMD; http://www.ks.uiuc.edu/Research/vmd/) and rendered with POV-Ray (http://www.povray.org/).

All blue links in Fig. 5A connect E1, E2 and p7 with other HCV proteins. Namely, p7 with NS4A, NS5B and NS3 (protease and helicase), E1 with NS4A and NS3 (protease and helicase) and E2 with NS5A and NS4A. These predictions suggest that E1 and E2 should be in the same cellular compartment as the NS proteins to ensure their interactions. In fact, the ectodomains of the former are located in the ER lumen while the latter are in the cytosol (see Fig. 1). However, the presence of HCV E2 protein in the cytosol has been previously observed^56^,^57^ (see “Discussion” section). These predictions of protein interactions must be validated experimentally.

The main important difference between the experimental network and the coevolution network lies in their level of resolution. Indeed, to date, the experimental network provides a “yes/no” answer to the question of the existence of an interaction between pairs of proteins (Fig. 5B, top), while the coevolution network provides, for each pair of proteins, information on potential interactions between protein domains and their interacting residues. It also highlights potential interactions among several proteins and not just between protein pairs (Fig. 5A, top). This information has important consequences, both for our understanding of the complex viral protein interaction systems and towards the design of molecules interfering with the virus. It should be experimentally assessed.

### Networks associated to different genotypes

HCV proteins appear to coevolve with each other and a visual representation of these quantitative mapping is reported in the squared matrices of Fig. 6 (left), where each squared matrix has as many rows and columns as the proteins in the HCV polyprotein of the various genotypes. The values in the matrix represent the amount of evidence for a coevolution link that results from coevolution analysis and they describe the number of residues in a protein that are involved (directly or indirectly) in the coevolution link. Such residues are identified through one or several coevolution clusters, and the values in the matrix are obtained as the sum of the values computed over each cluster. For instance, consider cluster 38 of genotype 4 and the interaction E1-NS5A (see Supplementary Figure 3). In this cluster, there are 1 position from protein E1 and 3 positions from protein NS5A, and we count 1 + 3 as a weight for the E1-NS5A interaction. This value will be added up to three more weights coming from the three remaining clusters of genotype 4 that contain positions for both E1 and NS5A (clusters 39, 43, 46, Supplementary Figure 3). Each of these clusters contain 1 position for E1 but 3, 2 and 1 positions for NS5A respectively. By adding up the four weights together, we obtain 13 in the cells E1-NS5A and NS5A-E1 (the matrix is symmetric) as illustrated in Fig. 6 (left). From the E1 and NS5A positions in cluster 38, we also need to add 3 to the entry NS5A-NS5A of the matrix, since these residues might correspond to intra-protein interactions. Note that no contribution will be made by cluster 38 on the entry E1-E1 because a single position cannot be evidence for an intra-coevolution link.

These calculations were done for all clusters (for data availability, see Methods). Therefore, the numbers in the entries of a matrix are the total number of positions summed on all clusters for the given genotype (if specified), or on all clusters of several genotypes (when “three genotypes” is specified). Three matrices correspond to the three sets of sequences reported here for different genotype analyses (1b-MD, 2b, 4; Fig. 6, top left) and a fourth one (Fig. 6, bottom left) corresponds to the analysis of clusters from genotypes 1b-MD, 2b, 4 considered together. Note that the thickness of the edges in the interaction network in Fig. 5A corresponds to this matrix entries.

The entries lying in the diagonal of the matrix represent intra-molecular interactions. They can be also studied by analysing each protein separately as reported in Fig. 1B. Visual inspection of the resulting coevolving residues highlights many pairs of residues (coloured the same way in Fig. 1B) located on different secondary structure elements and facing each other in space, clearly showing that they likely play an intra-molecular role in the formation and/or the stability of the protein tertiary structure.

Longer proteins are likely to have more predicted coevolution links. (For HCV protein sizes, see Fig. 1A.) Indeed, as one observes in Figs 5A and 6 (bottom left), the large proteins NS5A and NS5B have a larger number of coevolution links than other proteins. To understand better this output, we re-did the analysis by considering domain-domain coevolution links instead. The issue of the protein length is partly resolved because longer proteins are split into a larger number of domains of more comparable sizes. The resulting domain-domain matrix, for the “three genotypes” confounded, is reported in Fig. 6 (right). The corresponding matrices for genotypes 1b-MD, 2b and 4 are reported in Supplementary Figures 4–6, respectively. One observes that links are not uniformly distributed over all domains, indicating that only certain domains are involved in protein-protein interactions.

The analyses of both protein-protein and domain-domain coevolution links realized on specific genotypes highlights that the links (as well as the absence of links) identified within a genotype are generally confirmed by the others. To statistically evaluate the similarity of the matrices describing domain-domain coevolution, we computed the Spearman correlation coefficient between pairs of matrices and obtained *ρ* = 0.39 for genotype 1b-MD vs 2b, *ρ* = 0.28 for 1b-MD vs 4 and *ρ* = 0.33 for 2b vs 4. The three coefficients have a p-value < 2.2*e* − 16, stating their high statistical significance.

In conclusion, some HCV domains are involved in a lot of coevolution links (red colors in Fig. 6) while many others are involved in a very small number of interactions (pale yellow colors in Fig. 6), possibly none (white in Fig. 6). The comparison of coevolution analysis based on different genotype datasets confirms that coevolution signals concern specific domains in proteins, and that a highly/lowly involved domain remains highly/lowly involved in all genotypes analysis.

### Predicted inter-protein coevolution links versus reported experimental protein-protein interactions

Due to the flexible nature of protein structures, protein physical interactions are expected to be specific, *i.e.* they are established through particular interaction sites at the surface of the protein, but not necessarily precise, in the sense that neighbouring residues might be more or less involved in the interaction (several concepts have been introduced to analyze such observations^58^,^59^,^60^,^61^,^62^,^63^,^64^ in the past). Consequently, a genotype correlated mutation is expected to highlight a “zone” in the protein surface where the direct physical interaction takes place, such as a patch of residues on the protein surface or a specific domain, but without necessarily identifying the same interacting residues highlighted by another genotype analysis. An example is reported in Fig. 7 where we compare the prediction highlighted by coevolution analysis to experimental results obtained by NMR on HCV proteins NS2 and p7^65^. Our coevolution analysis predicts an interaction between the two hydrophobic residues F14 of NS2 and I19 in TM1 of p7 (coloured red in Fig. 7). The residues experimentally identified by NMR to be in interaction are V15 and A12 of NS2 and W48, located in TM2 of p7 (coloured green). Among those, W48 and V15 are 100% conserved in genotype 2b and thus cannot be detected by coevolution analysis. We found that I19 and F14, which is very close to A12 and V15, coevolved. This prediction does not contradict the experimental finding but rather supports them by identifying an additional potential interaction between P7 and NS2. The veracity of the predicted interaction between residue 14 of NS2 and residue 19 of p7 could be tested experimentally by mutating one of these residues and searching for the emergence of the complementary mutation in the other protein after several cycles of viral replication in HCV infected cells. Thus, one can expect that coevolution interactions predicted with BIS^2^ could be useful to predict protein-protein physical interactions and guide experimentalists searching for protein-protein interactions.

A typical example to be explored through experiments is illustrated in Fig. 8, where the coevolution links between the four residues (colored magenta) located on NS5B surface and those observed in NS3, NS5A and E2, support the hypothesis of possible physical interactions between these coevolving residues.

### Predicted intra-protein coevolution links

Coevolution methods do not discriminate between inter-protein and intra-protein interactions, as they simply detect coevolving residues, wherever they are. But intra-protein interactions can be useful because they allow us to verify whether the prediction method works on proteins with known structure. Indeed, for several HCV proteins, we know the 3D structure (see Fig. 1B), so we can map the clusters discovered by coevolution analysis in each structure and see if residues in a cluster are compatible with the known three-dimensional structure: typically we expect to see pairs of coevolving residues corresponding to close positions in the 3D structure. By plotting all clusters on protein structures, one observes that many of them contain only two positions in a structure and that they are “close” to each other (<10Å as minimal atomic distance between pairs of residues, see Methods; this bound is coherent with previous analyses on predicted residue contacts^30^). This is for example the case for the green predicted coevolving residues of structure NS5B in Fig. 8. The two green residues have been detected in cluster 8 from genotype 4 sequence alignment (Supplementary Figure 3). At positions 239 and 293 of the alignment, the two green residues display either amino acids V and L, or amino acids I and V. The similar physico-chemical properties of the V, L, I residues as well as the presence of the V residue either in one position or in the other, are a good indicator of a physical contact. In contrast, the three residues coloured cyan in NS5B (Fig. 8; positions 47, 77, 517 deduced from cluster 4 of genotype 2b, Supplementary Figure 3) are most likely indirect intramolecular coevolution links. Spatial representations of the intramolecular coevolution links are reported in Supplementary Figures 7–11 for known structures E2, NS2, NS3, NS5A and NS5B.

We wished to evaluate whether the proximity of coevolving residues, as the green and magenta residues in NS5B of Fig. 8 or the black residues of cluster 39 for genotype 4 in NS5A of Fig. 9, is statistically relevant or not. To check this in the NS5A and NS5B proteins, we counted the number of predicted close interactions and compared it to the distribution of close interactions obtained with a null model. The null model has been constructed by randomly shuffling positions in predicted interactions (permutations have been realised separately, in each one of the proteins). We repeated this reshuffling 10000 times to estimate the p-value of the original distribution. Strictly speaking this procedure has been realised over the full HCV polyprotein to avoid multiple testing and gain statistical power. (The analysis was done on the following proteins: E2, p7, NS2, NS3, NS5A, NS5B, for which at least a part of the three-dimensional structure is known). To do this, we counted the number of predicted interactions over the HCV polyprotein and compared them with the null distribution, consisting of a random permutation of residues in each protein while keeping the same cluster organisation. The p-value we obtained is 0.0017, corresponding to <1% of known interacting intra-pairs of residues. (This corresponds to 13 interactions identified on the HCV polyprotein against an average of 5.13 interactions obtained in the simulations.) This value is clearly statistically significant and we can conclude that intra-protein coevolving positions detected by BIS^2^, as in NS5A and NS5B, tend to be close to each other more often than expected by chance.

**Figure 2:**
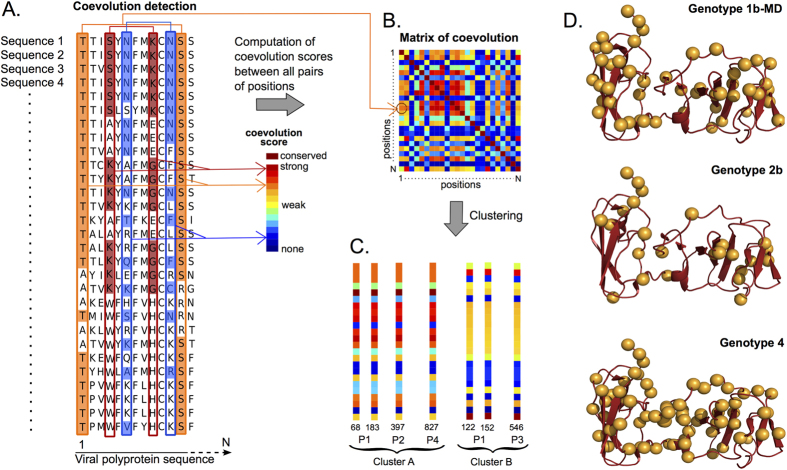
Methodology for coevolution analysis. Pipeline of BIS/BIS^2^ coevolution analysis: (**A**) The BIS method first detects coevolving residues among each pair of alignment positions and associates a coevolution score to the pairs. In the toy sequence alignment shown here, we report the analysis of three pairs of positions (1, 12), (4, 9) and (6, 11). The coevolution score associated to the amino acid distribution in a pair of positions is represented by a color (color range from blue/absence to dark red/strong signal of coevolution). For best visualisation, on each column, conserved blocks of residues are, in alternation, coloured and left white. As an example, the average score of (4, 9) (dark red) corresponds to the strongest coevolution score and reflects the fact that an amino acid at position 4 appears always coupled with the same amino acid at position 9 (K coupled with G, for instance). In contrast, the average score of (1, 12) (orange) reflects the fact that the amino acids T and A in column 1 are roughly positioned in front of the amino acids S and R in column 12, respectively. On the other hand, the average score of (6, 11) (blue) corresponds to the weakest coevolution score and reflects the fact that a letter at position 6 appears often coupled with a different letter at position 11. For (6, 11), note R coupled with L and K. (**B**) BIS constructs a coevolution score matrix, for pairs of positions in the sequence alignment. Colors in the matrix correspond to coevolution scores. For instance, the score for the entry (1, 12) (circled) is coloured orange as in A. The brown diagonal in the matrix highlights that a position is evaluated against itself. (This color is labelled “conserved” and it ranges outside the color scale for coevolution.) (**C**) The third step in BIS clusters the coevolution matrix in B and identifies groups of positions displaying the same coevolution scores with all other positions in the alignment. The schema illustrates two fictitious clusters, one made of 4 positions and the second made of 3 (involving 3 and 2 proteins, respectively), where coevolution scores correspond for each position in the cluster. Positions in a cluster might belong to different proteins *P*_*i*_ of the polyprotein, as for cluster A where residues 68 and 183 belong to *P*_1_ and residue 397 to *P*_2_. (**D**) Structure of the NS5A protein (PDB entry 1ZH1) where only residues that are not 100% conserved in the sequence alignment associated to genotypes 1b-MD (top), 2b (middle) and 4 (bottom) are represented with yellow balls. They are 50, 24 and 71 for 1b-MD, 2b and 4 respectively (for a total of 163 residues in 1ZH1). Fully conserved positions are represented by a dark red cartoon. Note that if we eliminate also the positions that are 100% conserved with the exception of one sequence, as done in BIS^2^, only 31, 18 and 45 residues of the NS5A protein will be analysed for genotypes 1b-MD, 2b and 4 respectively.

**Figure 3:**
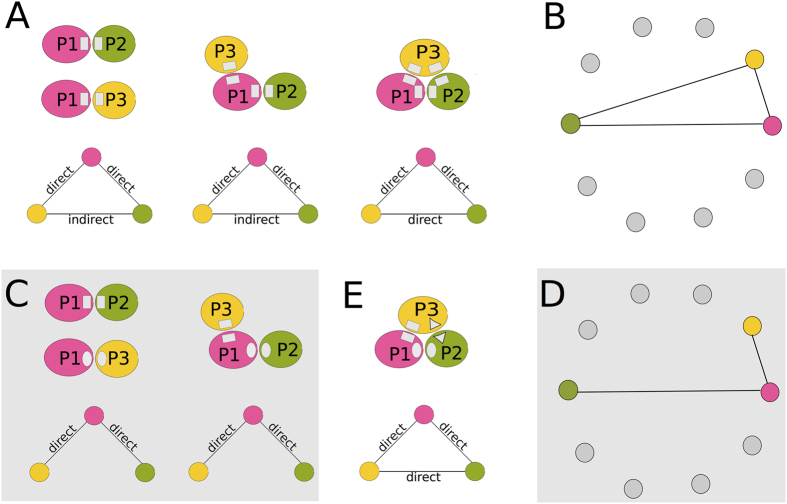
Protein interaction and the construction of a dependency network. (**A**) Three different kinds of relations among three interacting proteins (top) are illustrated. They involve either direct physical interactions or indirect interactions based on a mediator protein. For each kind of relation, a network is constructed where nodes correspond to proteins and edges correspond to interactions taking place through the physical binding of the proteins (bottom). Proteins (top) and nodes corresponding to them (bottom) are coloured pink, green and yellow. Note that the physical binding in the three interaction schemas are represented by the same rectangular symbol, indicating that there are residues involved in the interaction that belong to the same cluster. (**B**) Dependency network associated to three interacting proteins, satisfying one of the relations illustrated in A or in E. The colours of the nodes in the graph correspond to the colours of the proteins in the schema A and E. Notice that edges in the network correspond to direct or indirect inter-protein interactions (solid edges in A). Intra-protein interactions are not represented. (**C**) As in the first two interaction schema illustrated in A but where residues involved in the interaction belong to different clusters (rectangular, circular, triangular). (**D**) Dependency network associated to the two interaction schema in C (on a grey background). Nodes and edges as in B. Compare with B. (**E**) As in the third interaction schema illustrated in A but where residues involved in the interaction belong to different clusters (rectangular, triangular, circular).

As explained in Section “From interaction links to a network”, not all coevolving residue pairs are expected to be proximal in the structure. As an example, Fig. 9 shows the contact map of the NS5A structure (PDB entry 1ZH1) and the pairs of BIS^2^ coevolving residues. In the three-dimensional structure of NS5A, most residues analysed by BIS^2^ are in direct contact with conserved residues, that is residues that are ignored by coevolution analysis (see Fig. 2D and Supplementary Figure 12). Hence, NS5A coevolution analysis is not expected to provide any physically connected network of residues. Instead, one expects to identify pairs of positions that need to be mutated in concert to guarantee the functioning of the virus. The display illustrates the complete set of predictions realised on the non conserved residues of the three NS5A genotype datasets (Fig. 2D). It highlights a number of important general points for our HCV analysis:

1. The structural localisation of coevolving pairs suggests roles in homodimeric complex formation (Fig. 9 cluster 11 for genotype 2b), in collective movements (Fig. 9 - cluster 43 genotype 4 and cluster 9 for genotype 1bMD), in signalling (Fig. 9 - cluster 43 genotype 4; the conserved arm, illustrated in Supplementary Figure 12, connecting residue 197 to the rest of the protein might transmit signals), in interactions with other proteins (Fig. 9 - cluster 9 for genotype 1bMD and cluster 6 for genotype 2b), and in direct contacts within the crystallographic structure of the monomer (Fig. 9 - cluster 39 for genotype 4 and cluster 5 genotype 2b). In other words, coevolution signals can provide information on residue interactions all along the life of a protein and be indicators of protein interactions.
2. The information that can be extracted from the genome is very reduced, due to the high conservation patterns carried by the genotype sequences. This is illustrated in Supplementary Figure 13 (see also Fig. 2D), where the NS5A structure representing cluster 5 of genotype 2b indicates that, because of the full conservation of the amino acids in the sequences, no information can be extracted on the residues lying between residues 158 and 186, 188 in the 3D structure. Here, one would like to check whether a connected path of residues linking the three above residues exists or not but this check cannot be realized due to missing information, due to high conservation.
3. The crossed usage of multiple genotypes appears important to unravel insights into the protein functions and structure. For instance, cluster 9 for genotype 1bMD and cluster 6 for genotype 2b (Fig. 9) support pairs of proximal residues even if the two positions in each cluster are far apart in the structure. We predicted several such coupled signals involving different HCV proteins, and their identification augments confidence in cluster identification.

Finally, we analysed whether nucleotide mutations at the third codon position, known to behave “more or less” randomly^66^, could influence the detection of intra-protein coevolution signals at the amino acid level in HCV protein sequences. As expected, we highlighted a higher mutational rate for the third codon position compared to the first and second position, but with a strong bias due to a purine-purine and pyrimidine-pyrimidine substitution appearing with very high frequency. This means that mutations of HCV sequences are clearly not guided by a probability of substitution that is the same for all pairs of nucleotides at the third position. As a consequence, on the one hand, signals of coevolution at the nucleotide level on the third codon position can be identified with high probability, and on the other hand, because of the properties of the genetic code (that is, the transformation purine-purine and pyrimidine-pyrimidine does not change the amino acid), such substitutions remain synonymous and do not influence the coevolution signal at the amino acid level.

## Discussion

Many mechanisms of the functioning of HCV replication remain unknown and any insight on the HCV protein interactions could allow to make testable hypothesis on HCV activity. Here, we employed the coevolution analysis approach BIS^2^, to identify the HCV protein co-evolution network. BIS^2^ was able to uncover an important number of coevolving residues between HCV proteins which likely orchestrate the structural rearrangements and functions of various HCV multi-protein complexes involved in the replication of the virus. This large-scale network reconstruction for all HCV proteins is expected to unravel complex functional dialogs between multiple proteins coevolving together. However, these coevolution links remain predictions which need to be experimentally verified.

There are three different angles to look at this work: the data analysis with a novel way to exploit coevolution signals of viral sequences, the biology of the reconstructed protein-protein network, and the database of potentially interacting proteins described at a residue resolution. We shall comment on the three of them.

### Data analysis of viral sequences and coevolution signals

The very strong conservation and the limited number of genotype sequences demand to interpret the predictions accordingly and to establish multiple testing to augment the statistical confidence in the predictions.

#### The choice of appropriate sets of viral genome sequences is crucial

A primary limitation of current coevolution analysis approaches relies on the availability of a large number of evolutionarily-related sequences that are sufficiently divergent (but not too divergent, see below). Such sets of sequences constitute the bottleneck for today coevolution analysis methods^39^ (see also^26^,^27^,^28^,^37^). In this study, we demonstrate that BIS^2^ goes beyond these limitations and show for the first time that a coevolution analysis method can address coevolution of conserved sequences such as selected viral genotype sequences of full-length polyprotein of HCV to identify direct and indirect protein interactions and contacts. It must be stressed however that sequence differences between HCV genotypes (see Supplementary Figure 13) appeared to be too large to produce accurate data when submitted to BIS^2^ analysis. Even sequence divergence within certain genotypes were too high to yield workable coevolution information. Typically, for genotype 1b for which several hundreds of full-length HCV polyprotein sequences have been reported, we restricted our coevolution analysis to 40 sequences from a limited set of Japanese patients. This is linked to the fact that HCV infection is a highly dynamic process, with a viral half-life of only a few hours and production and clearance of approximately 10^12^ virions per day in a given individual^67^. This high replicative activity, together with the lack of a proofreading function of the viral RdRp NS5B, is the basis of the high genetic variability of HCV. Indeed, HCV exists within its host as a pool of genetically distinct but closely related variants, referred to as quasispecies^68^. This confers a significant survival advantage, as the simultaneous presence of multiple variant genomes allows rapid selection of mutants better suited to new environmental conditions. The fittest infectious virions are continuously selected as a result of selective pressures exerted by their interactions with host cell proteins and host immune responses. Hence, the coevolution of HCV proteins in infected patients is most likely a very rapid process. In addition, whereas the presence of inter-genotypic and intra-genotypic recombinants within the selected sets of sequences can be excluded, one cannot rule out the possibility of recombination between quasispecies, which remains undetectable by phylogenetic analyses. Consequently, the coevolution analysis of HCV sequences of patients distantly infected by the same genotype is garbled by numerous cycles of protein coevolution events experienced by their respective HCV quasispecies over time and possible intra-quasispecies genome recombinations. In contrast, HCV polyprotein sequences collected from a patient over time or a set of patients initially infected by the same virus source are likely related by a limited number of coevolution cycles. It is thus expected that coevolution analysis of such set of sequences should highlight primary protein-protein interactions.

#### Different evolutionary scenario underly the detected signals

For HCV genotypes, mutations appear in highly conserved alignment positions, and a given coevolving position features only 2 or at most 3 amino acids, among which one is especially well represented. This intrinsic conservation was observed in each HCV alignment considered. In particular, we observed that there are three types of scenarios describing a given distribution of amino acids in the reference alignment from the topology of the associated tree^69^. Namely, mutations can be grouped in 1. a large subtree, 2. multiple subtrees, 3. a small subtree made of 2, 3 or 4 sequences. Mutations that appear in these three topological contexts are expected to be meaningfully related, but at different confidence levels. That is:

1. When the subtrees have large dimension, the persistency of a mutation within the subtree can be interpreted as a positive evidence of its potential biological interest. The more the signal persists along time, the more likely it has important structural or/and functional implications, because otherwise it would have likely changed thereafter.
2. When the subtrees are multiple (in HCV sequence alignments there are at most two), then the appearance of the same mutations in distinguished evolutionary branches is a good indicator of correlation^44^.
3. When the subtree is small, comprising for instance only 2 sequences, then the statistical relevance of the signal might be questionable^44^ since a mutation might happen in sites that are not crucial for function and structure. However, under the hypothesis that the signal is biologically meaningful, if we were to consider more sequences, the subtree supporting a specific mutation could potentially be much larger, leading back to scenario 1, or a new subtree with the same pattern of mutations could emerge leading back to scenario 2. Because genotype sequences are few and very conserved, it is therefore important to highlight to the user the potential importance of such mutations.

To take into account these different scenarios, the tree of sequences associated to each cluster, showing explicitly to the user the location in the tree where the mutations were identified, is given (see Supplementary Figure 14). The list of hits is furnished (as a label of the leaves in a tree) for an easy reading. Based on this information, the user can properly distinguish the scenarios.

#### Conservation and missing information

High sequence identity within genotype sequences is characterised by a large number of positions that are 100% conserved or fully conserved except for one sequence. It is important to keep in mind that BIS^2^ ignores these positions from coevolution analysis. As a consequence, one should not expect that coevolution analysis of genotype sequences would highlight networks of spatially close residues but rather residues that are not physically in contact and that are linked in the protein structure by chains of conserved residues. (See Fig. 9 and Supplementary Figure 12). In this context, parallel analysis of different genotypes could be of great help for identifying correlations between residues, that could be integrated by a proper data combination or clustering.

### On the biological significance of the reconstructed protein-protein interaction network

#### Coevolution analysis probes the HCV polyprotein interaction network

The three coevolution analyses, based on sets of selected genotype sequences 1b-MD, 2b and 4, already constitute a solid basis to define the HCV proteins coevolution network. Importantly, analyses of protein-protein coevolution links realised on these sets of sequences highlight that the links (as well as the absence of links) identified within a genotype are generally confirmed by the others (Fig. 6 left, and Supplementary Figures 4–6). In particular, analyses of domain-domain coevolution links highlight that coevolution concerns only particular domains identified as interacting across genotypes. They allow the identification of highly coevolving HCV protein domains, notably the central domain of E2 glycoprotein, the protease domain of NS3, domains D1, D2 and D3 of NS5A, and the thumb domain of NS5B. The three latter NS proteins constitute the heart of the HCV genome replication machinery including RNA-dependent RNA polymerase activity (NS5B), helicase activity (NS3), and RNA-binding protein NS5A thought to tether and protect newly synthetized viral RNA (reviewed in^51^). Although the existence of interactions between these NS proteins have already been assessed experimentally (see Fig. 5, right), the residues involved in these interactions have not been identified. One can expect that the coevolving residues identified by BIS^2^ between these proteins could be helpful toward this goal. However, although coevolving clusters are indicative of which residues in a structure are crucial to the protein structural stability, and/or functional activity, and/or interaction with other proteins, they do not tell us whether these residues establish direct physico-chemical contacts with each other. Typically, the coevolution links between NS5B and NS3, NS5A and E2 proteins reported in Fig. 8 are indicative of existing physical interactions between NS5B and the other proteins, but the veracity of such interactions remains to be demonstrated experimentally. One powerful approach to check these putative interactions experimentally would be the mutation of coevolving residues in one protein to induce the emergence of complementary mutations in the other proteins after several cycles of viral replication in HCV infected cells. This powerful experimental approach has been, for example, successfully used to identify the interaction network of NS2 protein with p7, E1 and E2, and NS3 proteins^70^. But such analyzes are extremely time-consuming in terms of molecular biology experiments and they rarely give interpretable results on a mechanistic level because it is far from obvious to target one protein position and to predict the effect of its substitution by a particular residue. Carefully designed experiments based on rational predictions are thus mandatory to target residues that are potentially the most interesting on the mechanistic level. In this context, our database of predicted HCV protein-protein interactions obtained from BIS^2^ coevolution analyses offers a set of highly relevant predictions, which not only indicate the coevolving residues of a given protein but also indicate which amino acid substitution should be done, as well as which compensatory mutation(s) is(are) expected.

#### Unexpected coevolution links reveal the complexity of the HCV lifecycle

Comparatively to the network of reported HCV protein-protein interactions determined by using various experimental methods (reviewed in^54^; Fig. 5, right), several new links are observed (colored blue in Fig. 5, left), especially the strong coevolution link between NS5A with E2. This link, together with the other already reported links between E1 and E2 envelop glycoproteins and nonstructural proteins NS3, NS5A and NS5B are a priori surprising since the globular domains of the latter NS proteins are located on the cytosolic side of the ER membrane while the ectodomains of E1 and E2 are on the ER luminal side (Fig. 1B). In this context, any physical interaction between E1 and E2 and the NS proteins seems impossible. However, However, interactions between glycoproteins E1 and E2 with various non structural proteins have already been identified experimentally, as summarized in Fig. 5B and in^54^. Moreover, it has been reported that an unglycosylated form of envelop protein E2 can exist in the cytosol and inhibits the kinase activity of PKR protein, a mechanism which may contribute to the resistance of HCV to interferon^56^,^57^. Moreover, while the N- and C-terminus of p7 protein are facing toward the lumen of the ER^71^, another topology was reported where the C-terminus is exposed towards the cytosol^72^. These examples illustrate that HCV ER proteins can exist in the cytosol and might thus physically interact with nonstructural HCV proteins. Alternatively, the strong coevolution links between E1 and E2 and the cytosolic domains of NS proteins could be due to indirect links mediated by some other HCV proteins, as a result of numerous coevolution cycles. While being enigmatic, these strong coevolution links between cytosolic and luminal protein domains might bring to light essential features of HCV life cycle that have not been yet identified. Based on the clusters of coevolution residues, this intriguing question could be addressed experimentally by searching for the emergence of compensatory mutations as detailed above.

### A database of predicted interactions to guide experimentalists

#### Evaluation of the statistical significance of BIS^2^ predictions

The coevolution analysis method BIS^2^ allowed the identification of many clusters of coevolving residues within and between HCV proteins, which likely correspond to essential structural and functional motifs involved in protein-protein interactions driving the assembly of protein complexes required for the replication of the virus. Several *in silico* statistical tests have been realized to evaluate the significance of the predictions:

‐ Independent analyses of different genotypes produced highly correlated domain interaction matrices (see section “A network of coevolution links”, Fig. 6 and Supplementary Figures 4–6), attesting the existence of similar coevolution patterns between protein domains in different genotypes.

‐ All clusters of coevolving residues predicted by BIS^2^ are provided with a p-value. This allows a user to screen the results and reason about the outcomes. (See section “Cluster filtering” in Methods.)

‐ An estimation of the statistical significance of pairs of correlated residues that are found in physical contact within HCV protein structures is given. Simulations were realised to compute this estimation. (See section “Predicted intra-protein coevolution links”).

These statistical tests help to gain confidence in the predictions.

#### A database of interactions

The list of clusters reported in Supplementary Figures 1–3 constitutes a helpful database of predicted HCV protein-protein interactions for researchers who wish to experimentally test the veracity of these interactions towards the identification of HCV protein complexes. Two main observations can be highlighted for the database.

**Figure 4:**
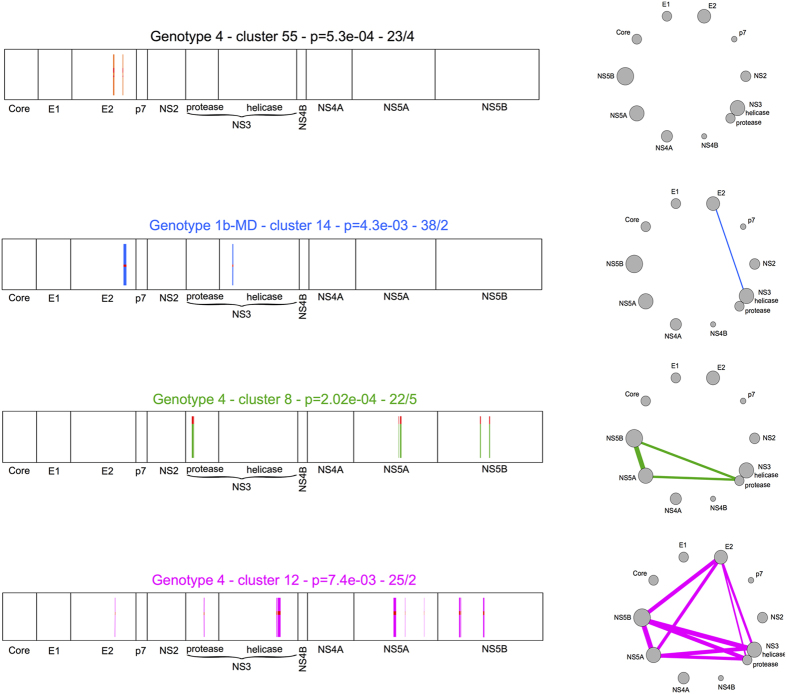
Construction of the coevolution dependency network. **Left:** Four coevolution clusters issued from BIS^2^ analysis of the HCV polyprotein sequences are reported (see Supplementary Figures 1–3 for an exhaustive list and more details). The HCV polyprotein is represented by a strip (as in Fig. 1A), and color bars represent coevolving positions along the strip. The strip is subdivided in 10 subparts scaled by the corresponding protein lengths. Above each strip, we indicate genotype name, cluster number, corrected p-value of the cluster and number of identical amino acids occurring in the alignment positions belonging to the cluster. For example, coevolving positions in cluster 55 (top) of protein E2 contain two residues, one occurring in 23 sequences and the other in 4 (“23/4”) over the 27 sequences forming genotype 4 alignment. The probability for these residues (see Methods) to occur in the proportion “23/4” is represented by the p-value 5.7*e*-5. **Right:** Coevolving networks describe clusters: nodes represent HCV proteins and edges represent coevolving links. The size of the nodes is proportional to the size of the proteins and the thickness of the edges estimates the number of links between protein pairs. From top to bottom, networks are constructed for clusters involving 1, 2, 3 and 5 HCV proteins. Intra-protein interactions are not displayed in a coevolution network (see, for instance, the network on top).

**Figure 5:**
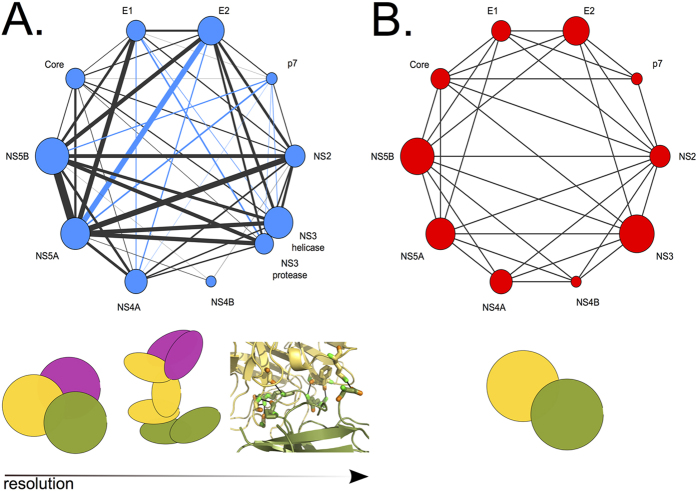
HCV protein coevolution dependency network. (**A**) The protein-protein coevolution dependency network reports the result of coevolution analysis realized on the full HCV polyprotein of “three genotypes” (1b-MD, 2b and 4, see text). The thickness of the edges is proportional to the number of predicted coevolution links (it corresponds to the numbers in the “three genotypes” matrix of Fig. 6, bottom left), and the nodes representing the proteins have an area proportional to the protein length. Figure 4 explains how the network is constructed, as the overlap of the networks associated to each coevolution cluster. Blue edges correspond to coevolving links that have not been experimentally reported (see B). The network contains, by construction, information on protein-protein interactions at different levels of resolution (bottom): among several proteins (through three-edge cycles and branching motifs, see main text), between specific domains, and on residues. (**B**) Network of reported HCV protein-protein interactions determined by using various experimental methods; adapted from Hagen2014. Edges represent the existence of interactions among pairs of HCV proteins (bottom). No information on multiple interactions, nor on interacting domains nor on residues involved in the interaction are associated to them. Compare to A.

**Figure 6:**
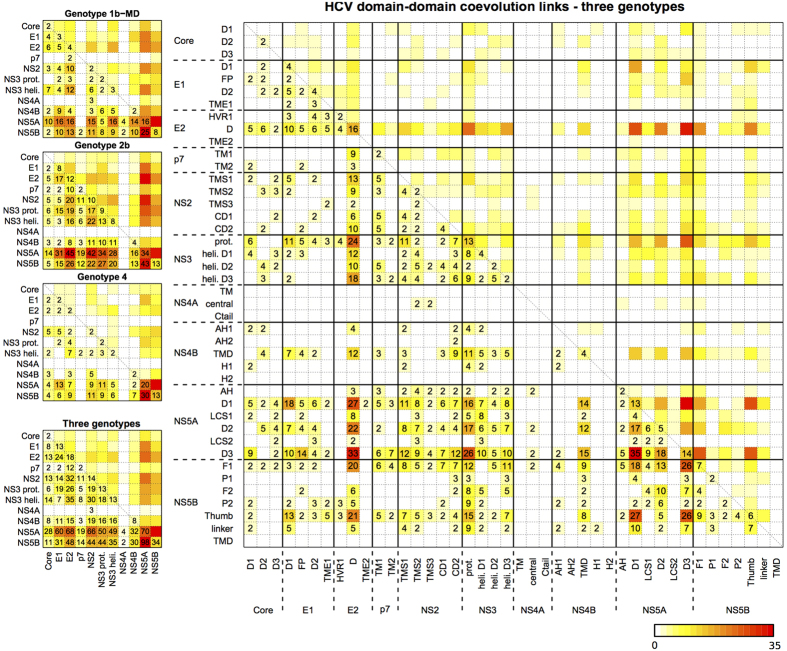
Matrices describing HCV coevolving proteins and domains. **Left:** The four matrices describe the analysis of protein-protein coevolution links for genotypes 1b-MD, 2b and 4 (top), and for the three genotypes confounded (bottom). Numbers in the matrices count coevolving residues (hits) belonging to clusters predicted by BIS^2^ analysis (see Methods). These numbers are intended to be indicators of the “amount of evidence for a coevolution link”. The diagonal (from the top left to the bottom right) of each matrix corresponds to internal coevolution links between residues in the same protein. The color scale used in each matrix corresponds to the range of the coevolving links computed for the matrix and it goes from red (maximum value for the matrix) to white (0). Note that the thickness of the edges in Fig. 5A corresponds to the entries of the matrix for the three genotypes (bottom). **Right:** The large matrix describes the analysis of domain-domain coevolution links, for the three genotypes 1b-MD, 2b and 4. It is the detailed domain-domain analysis of the protein-protein matrix shown on the top left. Thick black lines are drawn to delimit different proteins, while dotted lines delimit their domains. Detailed analyses for genotypes 1b-MD, 2b and 4 are reported as Supplementary Figures 4–6. Note that the sum of the entries for two proteins does not correspond to the value of the corresponding protein-protein entry in the matrix at the bottom left. Take, for instance, the interaction NS2-NS4A. Cluster 28 of genotype 1b-MD has 1 hit in NS4A and 2 hits in NS2, located these latter in the two different domains TMS1, TMS2. In the matrix on the bottom left, the entry NS2-NS4A is 3 since a total of 3 hits identifies the NS2-NS4A interaction. On the other hand, the domain-domain matrix reports 2 hits for NS4A-NS2/TMS1 and 2 for NS4A-NS2/TMS2, with a sum of the domain entries equal 4.

**Figure 7:**
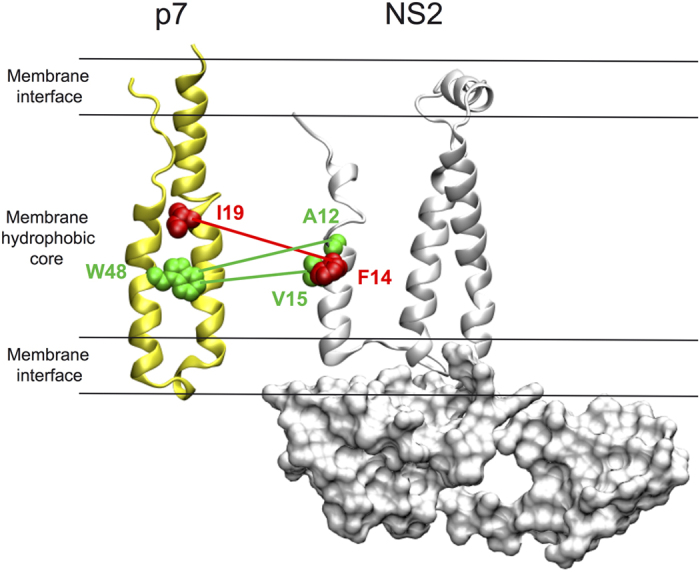
Interactions between HCV proteins p7 and NS2. The red line highlights a putative direct interaction predicted with coevolution analysis (data from cluster 39, genotype HCV-2b, see Supplementary Figure 2), while the green lines correspond to experimentally reported interactions^65^.

**Figure 8:**
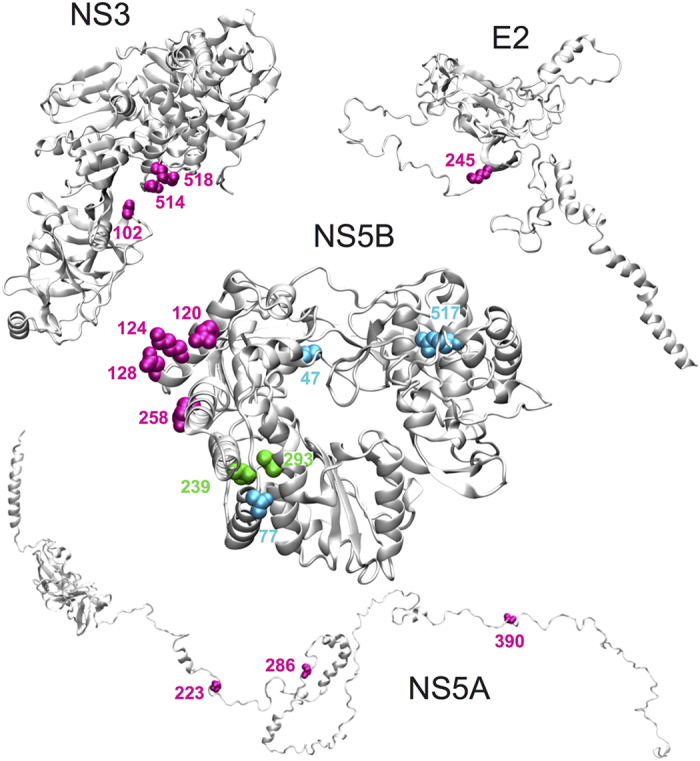
Direct and indirect coevolution links in NS5B. The intra-NS5B coevolving residues, issued from BIS^2^ analysis, correspond to the direct physical interaction 239–293 (green; data from cluster 8 of genotype 4, see Supplementary Figure 3), and indirect coevolution links for the three residues colored cyan (47, 77, 517; data from cluster 4 of genotype 2b, see Supplementary Figure 2). Coevolution links between NS5B and NS3 protease, NS3 helicase, E2 and NS5A involve four residues (magenta) located on NS5B surface (data from cluster 12 of genotype 4, see Supplementary Figure 3). Three of these residues (120, 124, 128) belong to the same side of an *α*-helix and the neighbouring fourth one (258) is located on the surface of another *α*-helix oriented on the same direction. These four residues, coevolving with residues in NS3, NS5A and E2 (also coloured magenta), support the hypothesis of existing interactions of NS5B with these proteins. Note that the four protein structures are not represented at the same scale.

**Figure 9:**
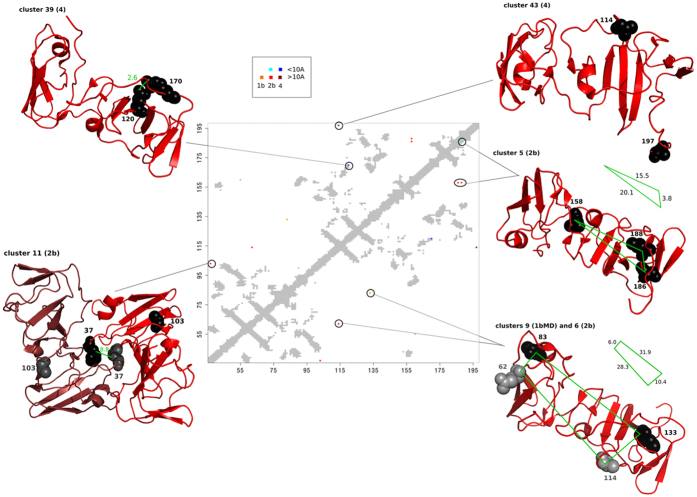
Intramolecular coevolution links in NS5A. Protein contact map representing all intramolecular coevolution links predicted in NS5A. Structural contacts at <10Å are indicated in grey. Contacts obtained with BIS analysis are coloured differently, depending on the genotype: 1b-MD in orange, 2b in red and cyan, and 4 in brown and blue. Orange, red and brown are used for residue pairs at distance >10Å, and cyan and blue for residue pairs at distance <10Å. For each coevolution link, the corresponding structure and residue localisation is given. Coevolving residues are represented by balls of the same color (back or grey).

First, this database collects relations between “groups of residues”, where mutations are present at the same time in face of the number of viral sequences and their variable homology. This ability to identify “groups of residues” determines the advantage of BIS^2^ computational platform over other technologies and makes the database unique. In fact, our protein-protein interaction network (Fig. 5A), even though it expresses pairwise links through graph edges, has been constructed as a projection of information coming from “groups of residues” (that is, the network is a superposition of smaller graphs associated to clusters of residues, as in Fig. 4, right). This is a crucial difference between our computational network (Fig. 5A) and the experimental one (Fig. 5B), that was constructed from experimentally reported pairwise interactions (reviewed in^54^). Using groups of residues instead of either single mutated residues or pairs of mutated residues opens up new avenues to the structural analysis of viral proteins since the mutational landscape of viral sequences could be more deeply investigated with well designed experiments suggested by computational evidence of multiple residue correlations. In this way, the complexity of viral evolution, expected to rely on the plasticity of protein structures, might start to be systematically investigated. For instance, predicted groups of residues known to induce neutral effects when mutated alone, could be studied together and the deleterious mutational effects of the group could be evaluated. Such groups cannot be pinpointed by random guess, and computational methods, such as BIS^2^, are needed to predict them.

Second, because of the relatively high divergence of HCV sequences used in this work, this database is certainly incomplete. One can expect that the following up of HCV polyprotein sequences of a patient over time or a limited set of patients initially infected by the same virus source to limit the number of coevolution cycles should yield more workable data, especially concerning direct/physical intra- and inter-protein-protein interactions. It would be thus of highest interest to set up specific projects to collect such sets of HCV polyprotein sequences that do not exist to date.

Third, BIS^2^ provides predictions that need to be experimentally tested and it is intended to drive the biologist to formulate novel hypothesis. Inferences are realised on relatively few genotype sequences and this limited data availability should be kept in mind when predictions are examined. It should be stressed that, as done by other groups working on viral sequences^73^, our predictions rest on the idea that a substitution at one site should rapidly follow a substitution at another site if the sites are positively epistatic. If this is the case, one should be able to meaningfully exploit the correlation signal found in viral sequences, even if there are very few of them. This means that along the evolution tree associated to the sequences, one should be able to see fast changes through common substitutions. These substitutions will be more meaningful if they appear as soon as possible, that is either up in the tree. If we find such evidence in lower positions of the tree, it might be still meaningful to indicate to the user that a potential important signal is there.

In conclusion, the novel computational approach of coevolution analysis BIS^2^, which has been successfully used here for HCV proteins, can be used for interaction predictions for other viral genomes (possibly by exploiting a larger spectrum of analyses that can be realised with it, and that requires handling scores that are not as high as those used for HCV; see orange positions in Fig. 2 for an example), and we expect that it can be generalised to help elucidation of genome-wide protein-protein interaction networks.

## Methods

### Genotype sequences

HCV sequences are classified in 7 genotypes (numbered 1–7), most of which have multiple subtypes (denoted *a, b* and so on); these genotypes and subtypes differ in their nucleotide sequences on approximately 33% and 25% of their positions, respectively^74^,^75^. It should also be mentioned that inter-genotype and inter-subtype recombinants have been identified in the HCV-infected population (reviewed in^76^). From the pool of full-length HCV polyprotein sequences available in euHCVdb (https://euhcvdb.ibcp.fr)^77^ and shown in the associated distance tree in Supplementary Figure 13, we extracted three groups of non-redundant sequences of genotypes 1b (denoted 1b-MD), 2b and 4 on which we realised coevolution analysis.

- 1b-MD: 40 full-length HCV polyprotein sequences from a limited set of japanese patients of genotype 1b^78^,^79^ corresponding to accession numbers AF165045 to AF165064 and AF207752 to AF207774, except sequences AF165055, AF165056, and AF207759 which include either additional residues or gaps comparatively to the genotype 1b consensus sequence.
- 2b: 24 full-length HCV polyprotein sequences from different patients with genotype 2b corresponding to accession numbers AB030907, AB559564, AF238486, D10988, and AY232730 to AY232749.
- 4: 27 full-length HCV polyprotein sequences of various subtypes of genotype 4 corresponding to accession numbers DQ418782 to DQ418789 (except DQ418783 and DQ418785), EU392169 to EU392175, FJ462431 to FJ462441, FJ839869, FJ839870, GU814265, Y11604, EF589161.

To avoid the use of inter-genotypic recombinants, only “confirmed” genotypes were selected^74^,^75^. Genotypes 2b and 4 have been selected because they are represented by a sufficiently large number of sequences (24 and 27, respectively) and genotype 1b-MD because it has the unique characteristic of being associated to a very small number of japanese patients studied by Nagayama and co-workers^78^,^79^. Genotypes 3, 5 and 7 were excluded from coevolution analysis because they contained too few non redundant sequences (less than 15) and this would have made the statistical analysis too weak. Genotype 6 was also excluded because it is composed of sub-genotypes (6a, 6b, 6c…) with a relatively high degree of divergence and containing very few sequences. This implies a too weak detection of the coevolution signal. The available sets of full-length polyprotein HCV sequences for genotypes 1a and 1b are very large and quite divergent. Coevolution analysis realised on such sets produced no workable signal.

BIS^2^ analysis was performed separately on the three selected datasets and a total of 62 statistically significant clusters was identified. The union of the clusters from the three datasets was also analysed, and we refer to it as the “three genotypes” analysis.

### Structures and modeling of the HCV proteins

HCV structures used in the analysis are illustrated in Fig. 1B from left to right: (i) core protein includes the N-terminal natively unfolded domain (D1) containing a helix-loop-helix motif (PDB entry 1CWX^80^) and two amphipathic *α*-helices connected by a hydrophobic loop (D2 domain^81^) as well as the core-E1 signal peptide (PDB entry 2KQI^82^) cleaved by SPP. (ii) E1 glycoprotein ectodomain containing the crystal structure of the N-terminal domain (residues 1–79, PDB entry 4UOI^83^) and associated to its C-terminal transmembrane domain (residues 351–383^84^). (iii) E2 glycoprotein ectodomain containing the crystal structure of its core region (PDB entry 4MWF^85^) and associated to its stem region (residues 705–715, PDB entry 2KZQ^86^) and C-terminal transmembrane domain (residues 714–746^87^). (iv) Monomer model of p7 solved by nuclear magnetic resonance (PDB entry 2K8J^88^ and 2MTS^65^). (v) Monomer of NS2 catalytic domain (PDB entry 2HD0^89^) connected to its N-terminal membrane domain constituted of three putative transmembrane segments (PDB entries 2JY0, 2KWT and 2KWZ^70^,^90^). (vi) NS3 serine protease domain associated with the NS4A central protease activation domain and the N-terminal transmembrane domain and the NS3 helicase domain. This representation of NS3 (derived from PDB entry 1CU1^91^) indicates that the helicase domain can no longer interact with the protease domain when the latter is associated with the membrane through the transmembrane domain of NS4A (BMRB entry 15580^92^). (vii) NS4B with the N-terminal part, including two amphipathic *α*-helices (PDB entries 2LVG^93^ and 2JXF^94^), the central part harboring four predicted transmembrane segments, and the C-terminal cytosolic part, including a predicted highly conserved *α*-helix and an amphipathic *α*-helix interacting in-plane with the membrane (PDB entry 2KDR^95^). (viii) NS5A N-terminal amphipathic *α*-helix in-plane membrane anchor (PDB entry 1R7E^96^) connected to globular domain 1 (D1; PDB entry 1ZH1^97^) and intrinsically unfolded domains 2 and 3 (D2 and D3^98^,^99^,^100^). (ix) NS5B RNA-dependent RNA polymerase (RdRp) catalytic domain (PDB entry 1GX6^101^) associated with the membrane via its C-terminal transmembrane segment^102^.

### Mapping to the Con1 sequence

Since each analysis is run on a separate sequence alignment (sharing no common HCV genome), we need to map the alignment positions of the clusters to a reference genome sequence. For this purpose, we used Con1, a reference sequence of genotype 1b. The positions provided in the Excel file significant_clusters.xls are relative to the Con1 reference sequence (accession number: AJ238799).

To achieve this mapping, two steps are performed. First, each sequence in a dataset is aligned separately to the Con1 genome with MUSCLE v3.8.31 (see below), in order to find the sequence *S* in this dataset with the highest similarity to Con1, as measured by the fraction of Con1 residues that are aligned to an identical residue in *S*. Then, the mapping between alignment positions and Con1 is performed by using the mapping from the alignment positions of all sequences in the dataset to *S* (which is straightforward because *S* is in the alignment) and from *S* to Con1. It may happen that some detected residues do not map to residues in Con1 (because of gaps or the hyper variable sequence domains in E2 glycoprotein). In this case, they are discarded. If this elimination of residues leaves a cluster with less than two elements, the cluster itself is discarded.

### Sequence alignments

Alignments of sequences belonging to a single genotype were realised to prepare BIS^2^ input and were done with MAFFT v6.861b^103^ downloaded at mafft.cbrc.jp/alignment/software/. To translate BIS^2^ analysis positions for genotypes 2 and 4 into positions of the Con1 sequence, we aligned Con1 with sequences of genotypes 2 and 4 with MUSCLE v3.8.31^104^ downloaded at www.drive5.com/muscle/. This was done to gain in precision since sequences belonging to different genotypes are more divergent.

### Mapping to structures

When mapping alignment positions to protein 3D structures, a similar procedure as the mapping to Con1 is used, except that the Con1 reference genome is replaced by the sequence associated to the structure. The other difference is the use of a local alignment (Smith-Waterman) instead of a global alignment since, unlike for Con1, the structure will cover a single protein in the complete genome.

### Proximal coevolving residues in structures

We consider as “proximal” the pairs of coevolving residues whose minimal atomic distance is <10Å. To provide proximal residue pairs together with “almost reachable” pairs, we used a distance, based on C_*α*_ atoms, of <21Å. This distance roughly corresponds to a minimal atomic distance between residues of <10Å, for residues bearing long side chains (for example, lysine). In fact, note that a lysine (positively charged) extends from a lateral chain for about 6.5Å, and a glutamate (negatively charged) for about 4.5Å. Hence, when two residues belonging to two lateral chains point one towards the other, a minimal atomic distance of *x*Å between them can be approximated by a distance between C_*α*_ atoms of roughly *x* + 11Å. Hence, a threshold of 10Å for a minimal atomic distance corresponds to 21Å for a distance based on C_*α*_ atoms.

### Cluster filtering

Coevolution analysis provides a very large number of clusters that we filter to retain only those that are statistically significant. As a first filtering step, we considered only clusters with a perfect coevolution pattern, as illustrated in Fig. 2, where a residue changes in a column, at the same moment than in another column (these are positions detected by BIS^2^ coevolution analysis with symmetric and environmental scores = 1). Among those, we filtered out positions with a full conservation pattern. They consist of alignment columns with the same residue in all sequences, but also of columns with all but one sequence with the same residue. The second case can happen because we set the BIS^2^ parameter *d* to 1, which allows exactly one sequence to violate the detected coevolution pattern^45^.

On the remaining clusters, we performed a statistical test to evaluate the probability of observing the coevolution pattern by chance. Suppose we have a cluster where a hit column *j* has *k* sequences that have a particular residue and the *l* others have another residue. Because we used only clusters with symmetric and environment scores equal to 1, this property is true for all other hit columns of the same cluster. This means that any other hit column *j*′ in the same cluster has also *k* residues of one type and *l* residues of another type in the corresponding sequences. Therefore this *k*/*l* distribution is a property of the whole cluster and not only of a single position. In other clusters, we might have 3 different residues, in which case we have a *k*/*l*/*m* distribution.

For each *k*/*l* or *k*/*l*/*m* distribution, we performed a statistical test which measures the probability to observe this pattern by chance. We do this by performing a Fisher test on a 2 × 2 or 3 × 3 matrix, respectively for 2 or 3 different residues, with zeroes in all cells except the diagonal, which contains the integers *k* and *l* (2 residues) or *k, l* and *m* (3 residues). This Fisher test gives us a p-value, which measures the probability to observe such a good (or better) pattern by chance.

With this statistical test, we have a p-value for each cluster. We then use the Benjamini-Hochberg algorithm^105^ to adjust the p-values for multiple testing, which allows us to control the False Discovery Rate (FDR). In fact, we keep all clusters with an adjusted p-value ≤ 1%, and this corresponds to a FDR of 1%. We observe that this automatically excludes all clusters with a *k*/*l* distribution where *l* or *k* equal to 1. At the end, there are 62 remaining clusters (14 for 1b-MD, 20 for 2b and 28 for 4; see Supplementary Figures 1–3 for their description and p-value, and Supplementary Figure 14 for the associated trees of sequences).

Note that Supplementary Figures 1–3 report the description of 64 clusters instead of 62. Indeed, two of these clusters (cluster 31 for 2b and cluster 33 for 4) contain two positions of which one does not have a correspondance on the Con1 sequence. Hence, we eliminated these predictions from the analysis.

### BIS^2^: coevolution analysis of small sets of conserved sequences

The detection of coevolution patterns was performed with BIS^2^, the new version of the BIS algorithm (Blocks In Sequences). The description of the algorithm and the original implementation appeared in^45^. BIS^2^ is run for block coevolution analysis, which means that it tries to identify interacting protein fragments instead of just interacting residues. (BIS^2^ works on “block mode” by default.) To do this, each position of the alignment, called a hit, is considered as a starting point for a search of all other positions in the alignment that present the same distribution of amino acids as the hit. Each hit is extended to a block by considering the maximum number of positions around the hit that preserve the same distribution. These blocks represent protein fragments. Even though the analysis is realized on blocks, clusters are collections of hits, that is individual positions (possibly belonging to the same fragment) showing specific coevolution patterns.

Note that the BIS method has been designed for alignments with different conservation levels and it can be parameterized accordingly. In the specific case of HCV alignments, we deal with very few and very conserved sequences. Consequently, we decided to consider the most stringent BIS scores for correlation identification. Indeed, BIS was applied in its full computational power but only very strong patterns of coevolution (those with scores of coevolution = 1) are reported here as statistically meaningful (with respect to a p-value threshold).

As indicated in^45^, BIS deals with correlations at scores = 1 without exploiting the distance tree of sequences that can provide information on the mutations needed to justify a residue distribution. The idea behind is that correlations scored 1 occur with a sharp (perfect) signal: the fact that there is no ambiguity in the pair of amino acids occurring at two specific positions of the alignment is in itself a strong support for the correlation. Note that we are dealing here with a very limited number of sequences and that, by definition, there is no statistical variation that can be taken into consideration in the evaluation. The method BIS^2^ is combinatorial in nature and exploits a different view from statistical methods. It is the regularity of a pattern (its “perfection”) and the distance from this regularity that are measured, with respect to minimal changes, induced by a few mutations. Contrary to this, statistical methods measure how distant two amino acids distributions are from noise.

### BIS^2^ implementation and parameters

In BIS^2^, the optimization of the code was oriented principally to improve the speed of the tool. BIS^2^ runs 100 times faster that the original one. This speed improvement was necessary to be able to run BIS on the complete HCV polyprotein including about 3000 residues. The main reasons explaining this speed increase are the following:

- The implementation of the algorithm was globally optimized.
- The program was re-written using the MATLAB programming language instead of Perl.
- The score calculation step, which is a critical part of the algorithm, is written in C++, which is faster than a MATLAB or Perl implementation.
- The clustering step (i.e. CLustering AGgregation algorithm, or CLAG for short) is now run only with Δ = 5% while computations were performed fo all four values 5%, 10%, 20% and 40% in the original BIS. Setting Δ = 5% is generally a good default choice, and values above 10% are rarely needed.

The parameters used for running BIS^2^ are *d* = 1 in “d + mode”, which means that we consider both hits with 0 or 1 exception together. For the clustering step, i.e. the CLAG algorithm^106^ that is included in BIS^2^, the parameter Δ = 5% was used. Of all clusters that were obtained, we only considered those with both symmetric and environmental score equal to 1, that is with maximum score. Such score values produce coevolving positions like the ones described in the example of Fig. 2.

#### Availability of data and tools

Genotype sequences and data produced by the HCV polyprotein analysis (alignments, list of clusters, annotated pdb structures) are found at www.lcqb.upmc.fr/HCV/. BIS is available at www.lcqb.upmc.fr/BIS/. BIS^2^ is made available upon request to AC. Note that clusters refer to residue positions in the Con1 sequence.

## Additional Information

**How to cite this article**: Champeimont, R. *et al*. Coevolution analysis of *Hepatitis C* virus genome to identify the structural and functional dependency network of viral proteins. *Sci. Rep.* **6**, 26401; doi: 10.1038/srep26401 (2016).

## Supplementary Material

Supplementary Information
