# Supplementary material for: Coevolution analysis of Hepatitis C virus genome to identify the structural and functional dependency network of viral proteins
Source: Sci Rep. 2016 May 20;6:26401. doi: 10.1038/srep26401 (PMC4873791; doi:10.1038/srep26401)
Supplement: Supplementary Information [file srep26401-s1.pdf]

Supplementary Material

Coevolution analysis of Hepatitis C virus genome to identify the structural and functional dependency network of viral proteins

R. Champeimont, E. Laine, S-W. Hu, F. Penin, A. Carbone

Supplementary Figure1 : Significant clusters of coevolving residues in selected sequences of HCV polyprotein of genotype 1b-MD (40 sequences).The HCV polyprotein is represented by a strip subdivided in 10 subparts scaled by the corresponding protein lengths. Above each strip, we indicate the cluster number, the number of coevolving positions (hits), the number of identical amino acids occurring in the sequence alignment positions belonging to the cluster, and the corrected p value. Bars represent coevolving positions, which are numbered below the bar accordingly to the polyprotein (in magenta). The corresponding coevolving residues are indicated close to the bars and colored accordingly to their observed frequency. For further detailed data, see http://www.lcqb.upmc.fr/HCV/.

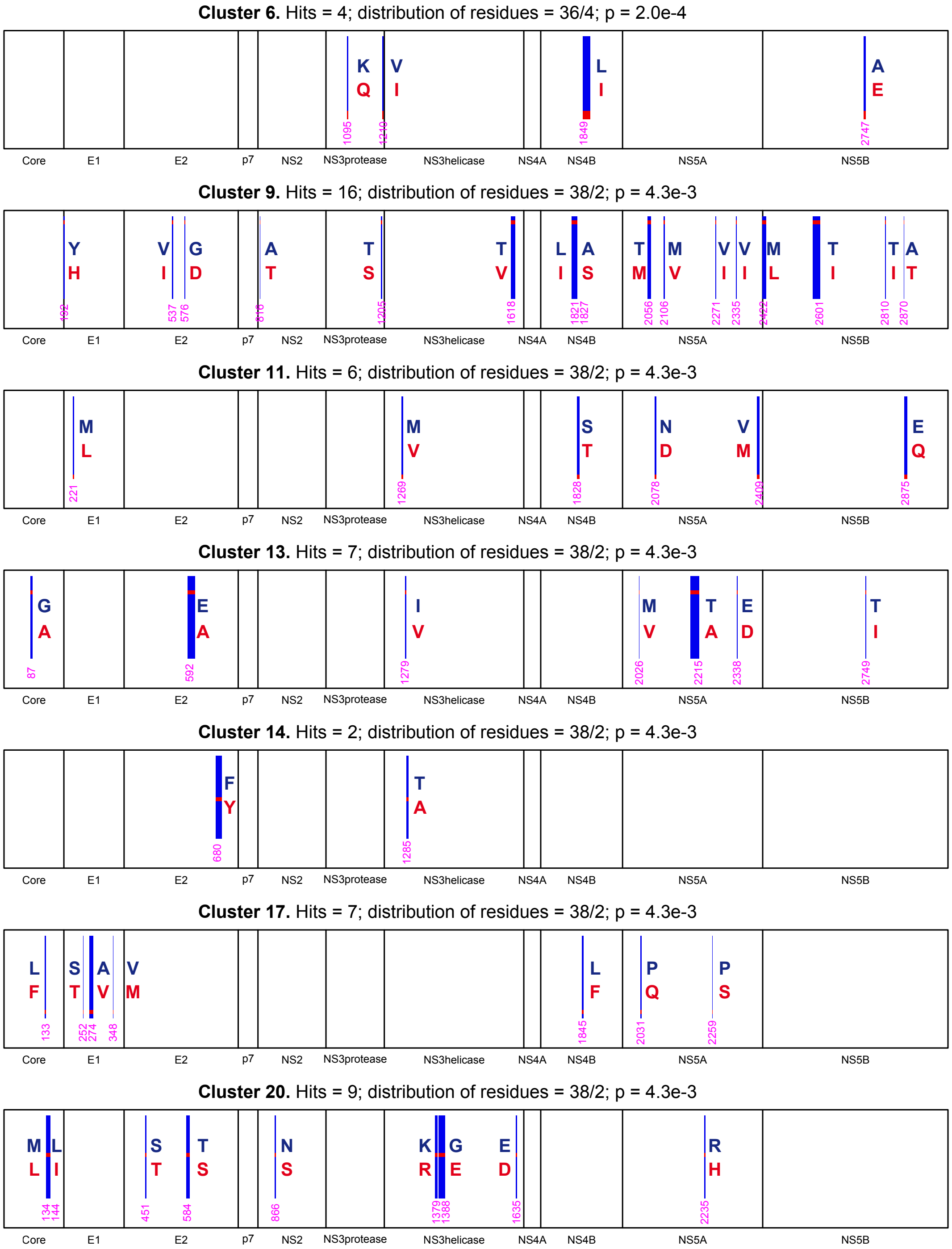

Cluster 28. Hits = 7; distribution of residues = 38/2; p = 4.3e-3

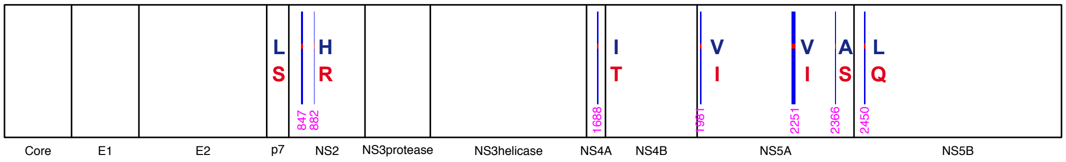

Cluster 29. Hits = 2; distribution of residues = 38/2; p = 4.3e-3

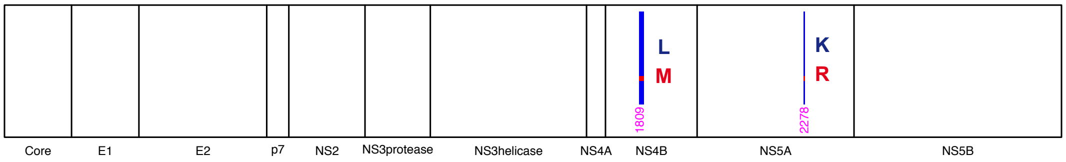

Cluster 32. Hits = 7; distribution of residues = 37/3; p = 6.6e-4

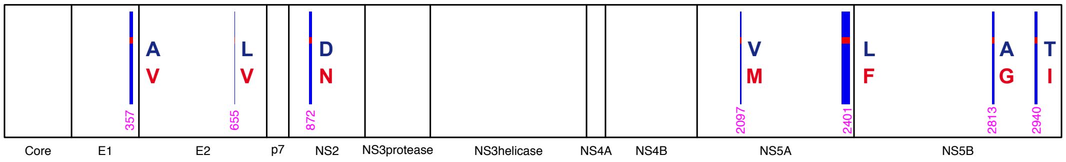

Cluster 36. Hits = 2; distribution of residues = 38/2; p = 4.3e-3

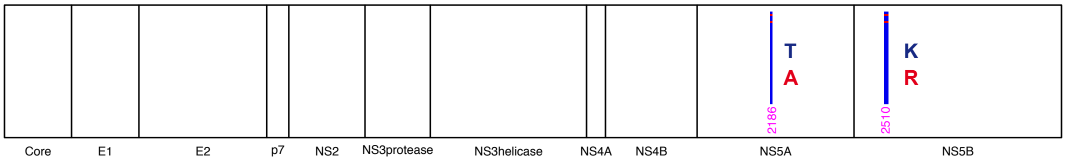

Cluster 39. Hits = 2; distribution of residues = 23/17; p = 1.5e-9

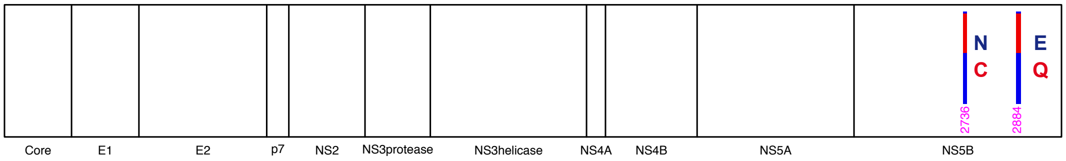

Cluster 40. Hits = 3; distribution of residues = 38/2; p = 4.3e-3

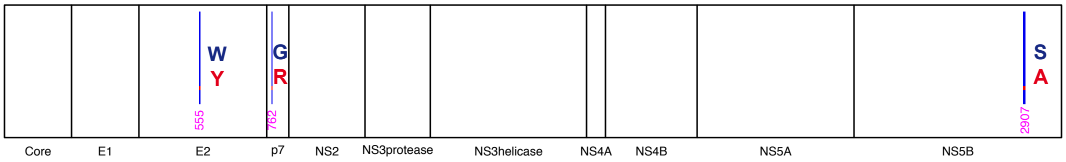

Cluster 41. Hits = 2; distribution of residues = 36/4; p = 2.0e-4

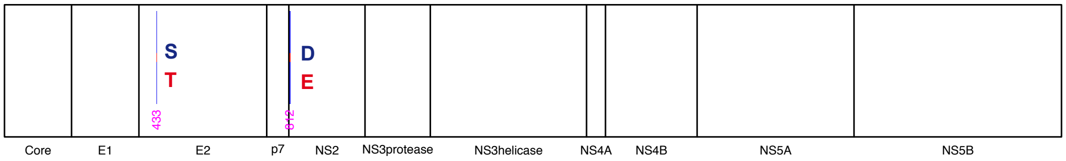

Supplementary Figure 2 : Significant clusters of coevolving residues in selected sequences of HCV polyprotein of genotype 2b (24 sequences).The HCV polyprotein is represented by a strip subdivided in 10 subparts scaled by the corresponding protein lengths. Above each strip, we indicate the cluster number, the number of coevolving positions (hits), the number of identical amino acids occurring in the sequence alignment positions belonging to the cluster, and the corrected p value. Bars represent coevolving positions, which are numbered below the bar accordingly to the polyprotein (in magenta). The corresponding coevolving residues are indicated close to the bars and colored accordingly to their observed frequency. Note that in crowded regions, some residues could not be indicated on the Figure. The corresponding position numbers are colored green and the corresponding residues are reported below by order of increasing frequency. Cluster 3, position 2342, residues G > E. Cluster 4, position 757, residues I > V; position 781, residues Y > H; position 881, residues E > Q. Cluster 5, position 2162, S > F. Cluster 6, position 2351, residues V > A; position 2353, residues S > G; position 2381, residues T > A; position 2413, residues P > S; position 2440, residues I > V. Cluster 8, position 1138, T > S. Cluster 12, position 330, L > I; position 373, residues A > V; position 385, residues T > H. For further detailed data, see http://www.lcqb.upmc.fr/HCV/.

Cluster 2. Hits = 12; distribution of residues = 22/2; p = 7.7e-3

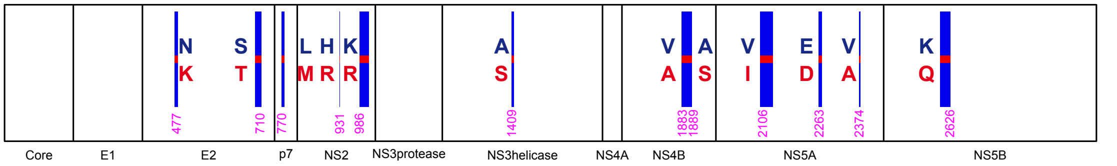

Cluster 3. Hits = 15; distribution of residues = 22/2; p = 7.7e-3

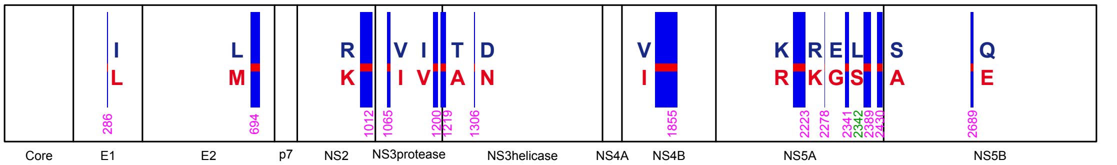

Cluster 4. Hits = 18; distribution of residues = 22/2; p = 7.7e-3

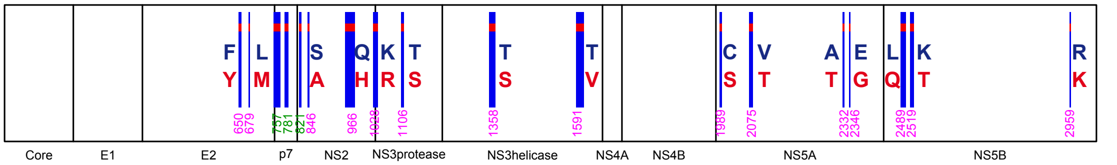

Cluster 5. Hits = 15; distribution of residues = 22/2; p = 7.7e-3

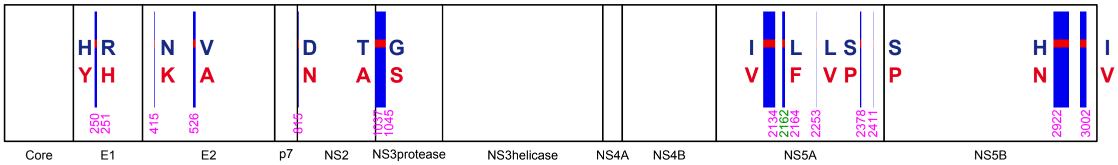

Cluster 6. Hits = 16; distribution of residues = 22/2; p = 7.7e-3

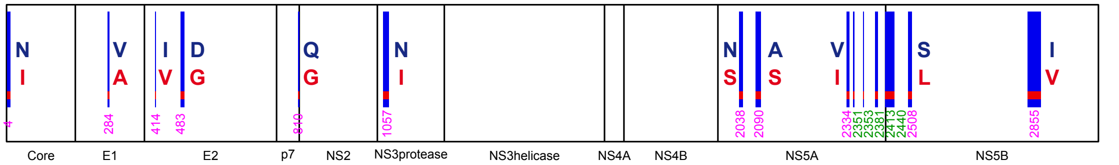

Cluster 8. Hits = 9; distribution of residues = 22/2; p = 7.7e-3

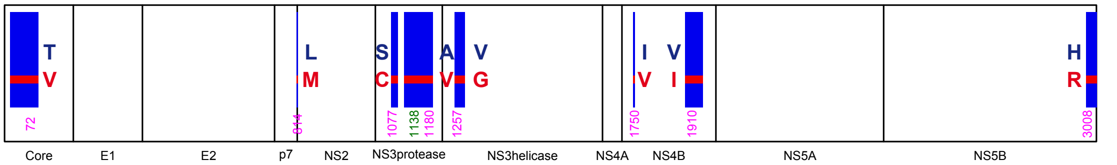

Cluster 11. Hits = 11; distribution of residues = 22/2; p = 7.7e-3

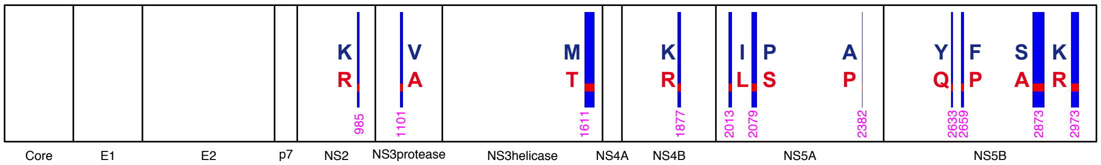

Cluster 12. Hits = 12; distribution of residues = 22/2; p = 7.7e-3

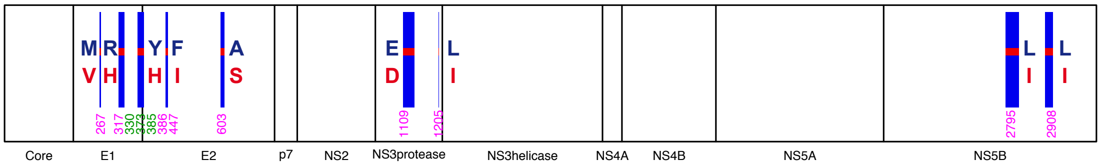

Cluster 20. Hits = 4; distribution of residues = 22/2; p = 7.7e-3

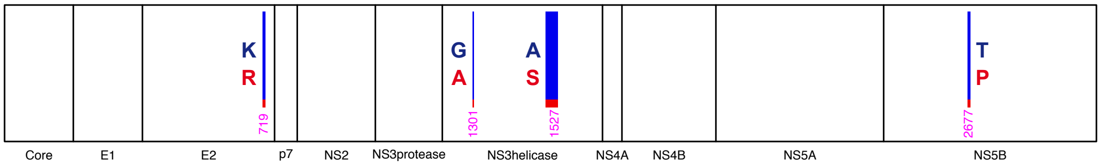

Cluster 21. Hits = 10; distribution of residues = 22/2; p = 7.7e-3

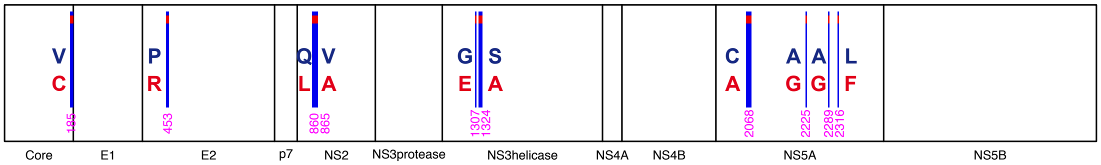

Cluster 22. Hits = 2; distribution of residues = 20/4; p = 6.5e-4

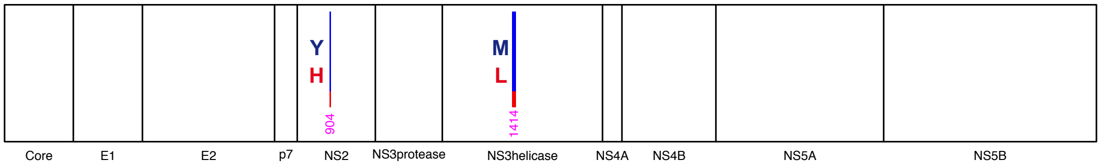

Cluster 24. Hits = 2; distribution of residues = 20/4; p = 6.5e-4

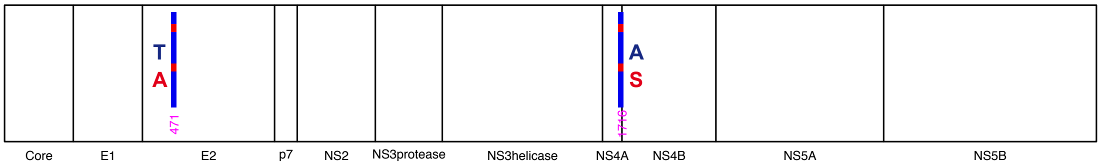

Cluster 26. Hits = 2; distribution of residues = 22/2; p = 7.7e-3

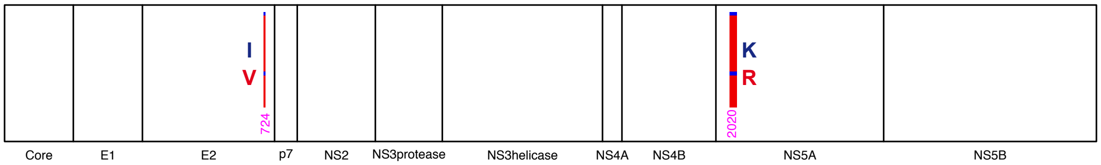

Cluster 29. Hits = 2; distribution of residues = 19/5; p = 3.1e-4

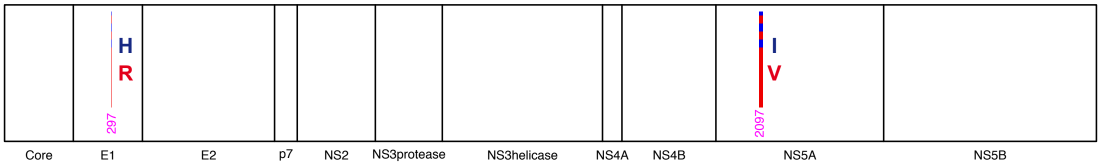

Cluster 31. Hits = 2; distribution of residues = 20/4; p = 6.5e-4

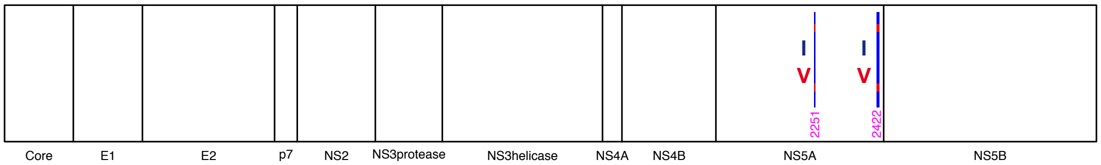

Cluster 32. Hits = 3; distribution of residues = 22/2; p = 7.7e-3

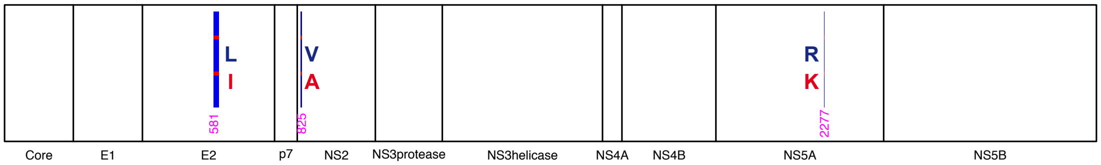

Cluster 34. Hits = 3; distribution of residues = 21/3; p = 2.2e-3

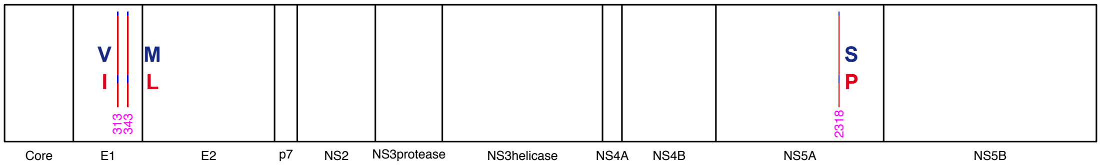

Cluster 36. Hits = 2; distribution of residues = 21/3; p = 2.2e-3

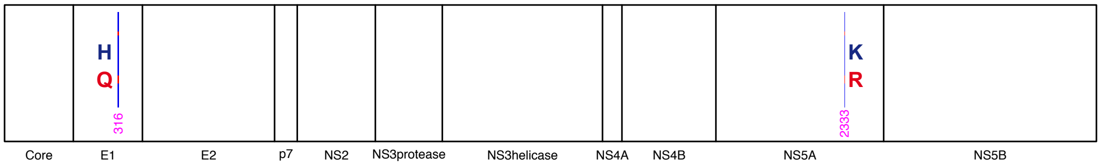

Cluster 37. Hits = 3; distribution of residues = 20/4; p = 6.5e-4

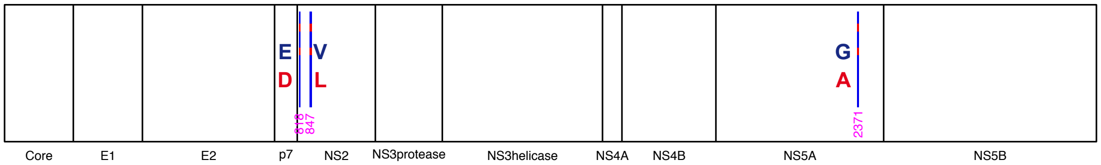

Cluster 38. Hits = 2; distribution of residues = 20/4; p = 6.5e-4

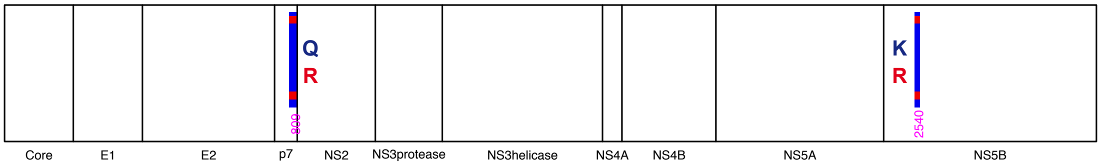

Cluster 39. Hits = 2; distribution of residues = 20/4; p = 6.5e-4

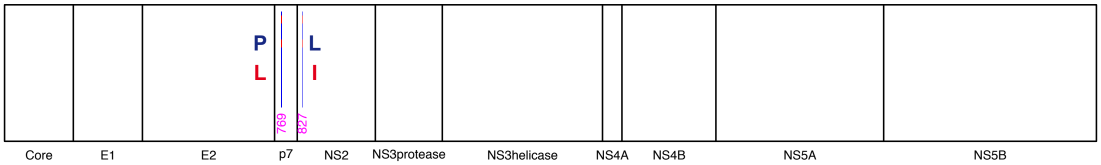

Supplementary Figure3 : Significant clusters of coevolving residues in selected sequences of HCV polyprotein of genotype 4 (27 sequences).The HCV polyprotein is represented by a strip subdivided in 10 subparts scaled by the corresponding protein lengths. Above each strip, we indicate the cluster number, the number of coevolving positions (hits), the number of identical amino acids occurring in the sequence alignment positions belonging to the cluster, and the corrected p value. Bars represent coevolving positions, which are numbered below the bar accordingly to the polyprotein (in magenta). The corresponding coevolving residues are indicated close to the bars and colored accordingly to their observed frequency. For further detailed data, see http://www.lcqb.upmc.fr/HCV/.

Cluster 3. Hits = 6; distribution of residues = 24/3; p = 1.7e-3

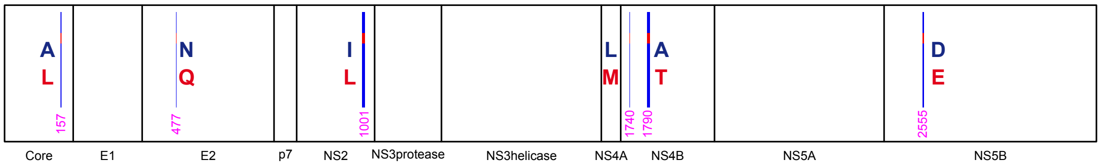

Cluster 4. Hits = 2; distribution of residues = 25/2; p = 7.5e-3

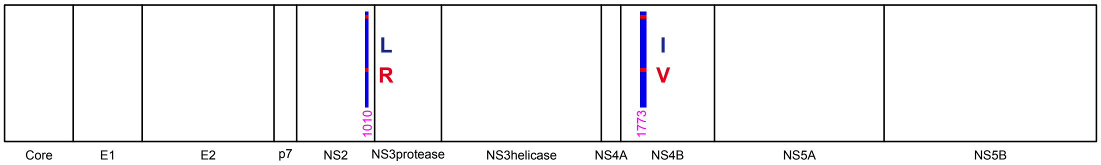

Cluster 6. Hits = 2; distribution of residues = 23/3/1; p = 2.1e-4

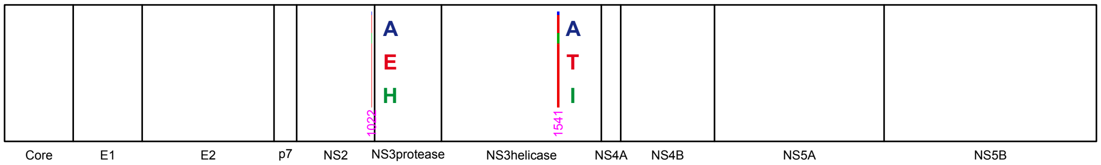

Cluster 8. Hits = 9; distribution of residues = 22/5; p = 2.0e-4

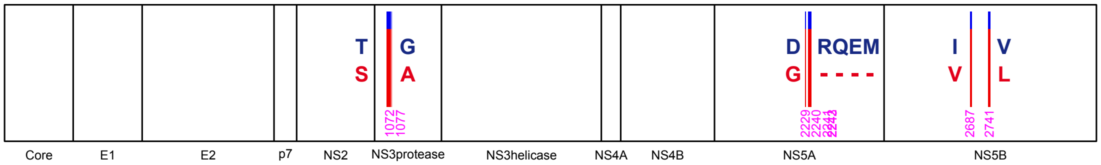

Cluster 11. Hits = 2; distribution of residues = 25/2; p = 7.5e-3

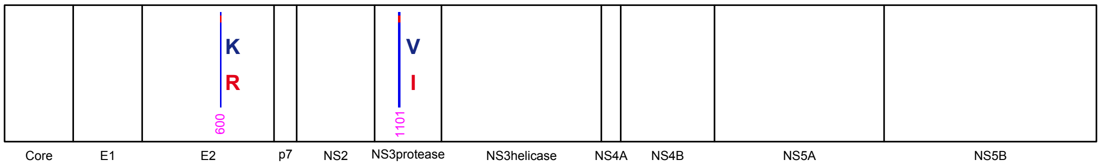

Cluster 12. Hits = 11; distribution of residues = 25/2; p = 7.5e-3

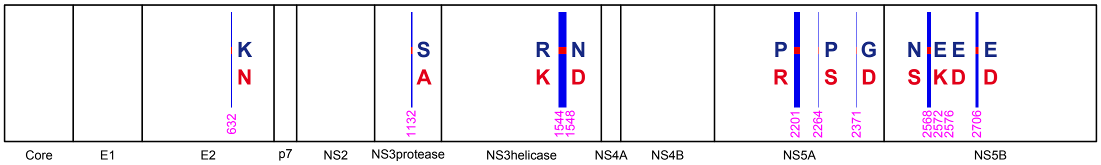

Cluster 20. Hits = 2; distribution of residues = 18/9; p = 1.4e-5

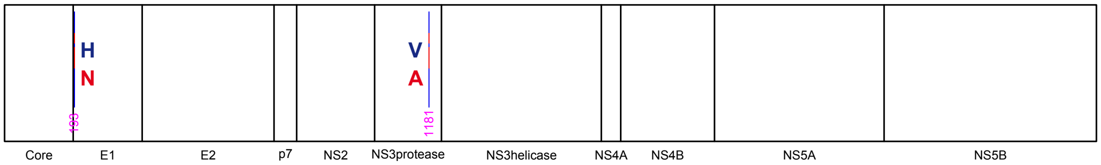

Cluster 28. Hits = 6; distribution of residues = 25/2; p = 7.5e-3

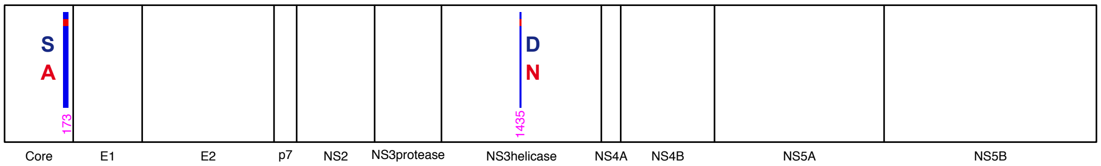

Cluster 29. Hits = 2; distribution of residues = 25/2; p = 7.5e-3

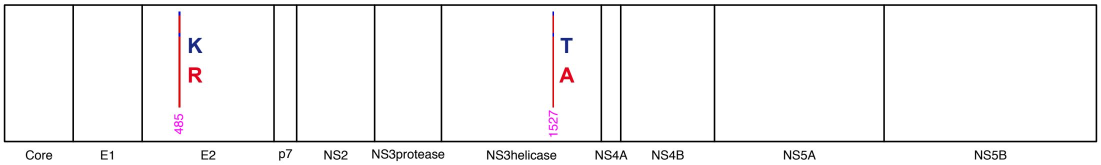

Cluster 30. Hits = 2; distribution of residues = 24/2/1; p = 6.8e-4

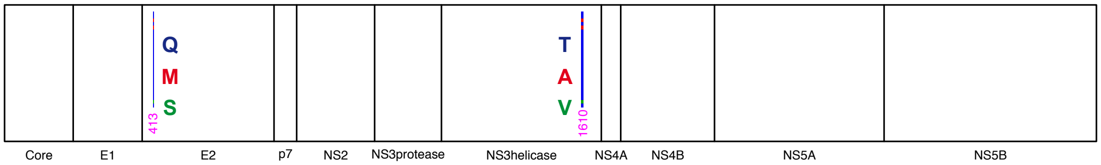

Cluster 32. Hits = 2; distribution of residues = 23/4; p = 5.3e-4

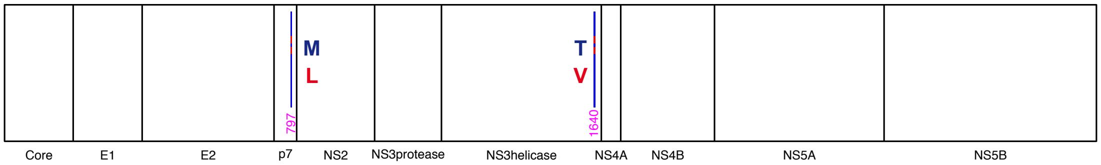

Cluster 33. Hits = 2; distribution of residues = 22/4/1; p = 6.8e-4

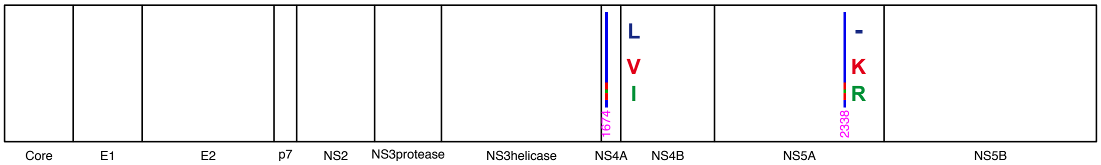

Cluster 34. Hits = 2; distribution of residues = 24/3; p = 1.7e-3

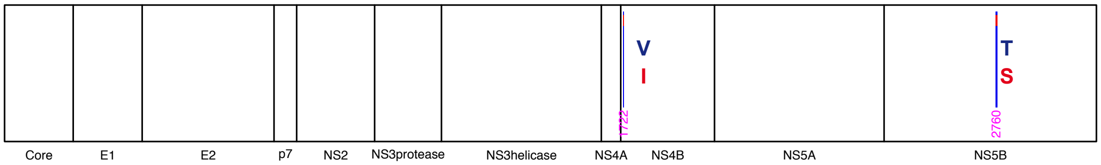

Cluster 35. Hits = 3; distribution of residues = 20/7; p = 4.9e-5

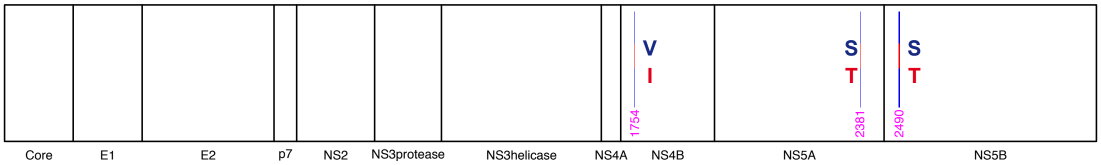

Cluster 38. Hits = 8; distribution of residues = 24/3; p = 1.7e-3

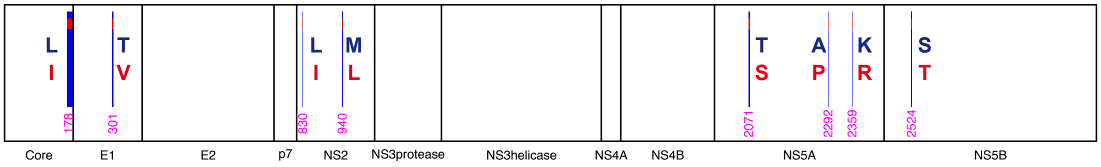

Cluster 39. Hits = 8; distribution of residues = 22/5; p = 2.0e-4

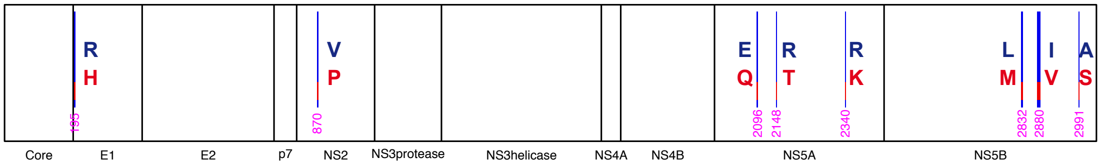

Cluster 40. Hits = 2; distribution of residues = 24/3; p = 1.7e-3

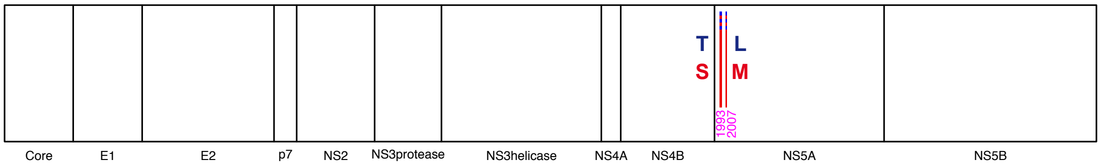

Cluster 41. Hits = 2; distribution of residues = 25/2; p = 7.5e-3

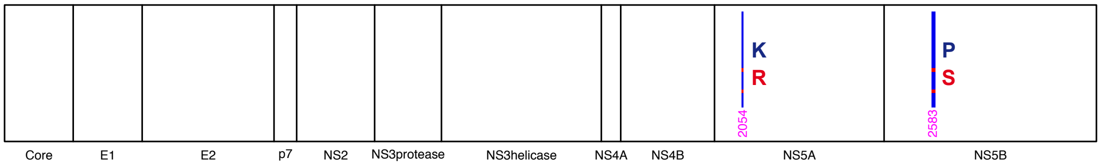

Cluster 42. Hits = 2; distribution of residues = 25/2; p = 7.5e-3

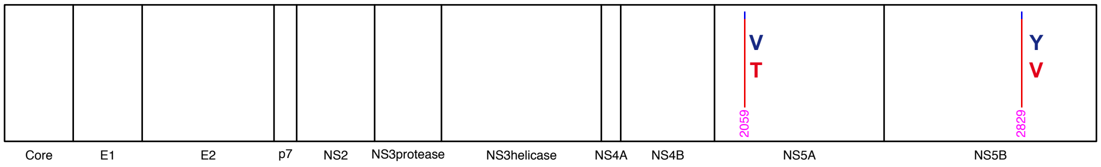

Cluster 43. Hits = 3; distribution of residues = 25/2; p = 7.5e-3

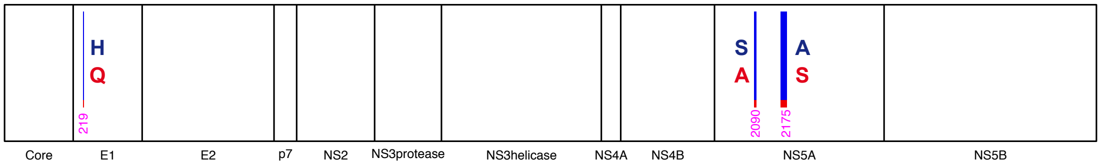

Cluster 44. Hits = 3; distribution of residues = 23/4; p = 5.3e-4

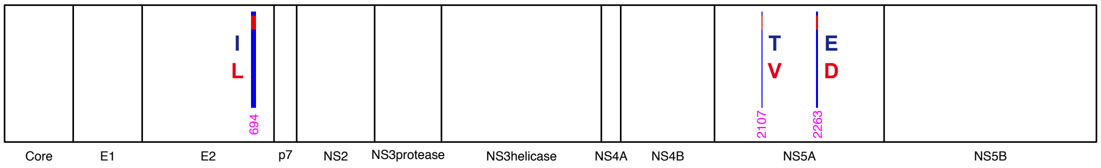

Cluster 46. Hits = 2; distribution of residues = 25/2; p = 7.5e-3

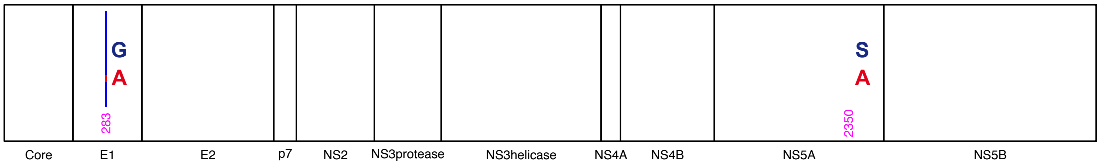

Cluster 47. Hits = 2; distribution of residues = 25/2; p = 7.5e-3

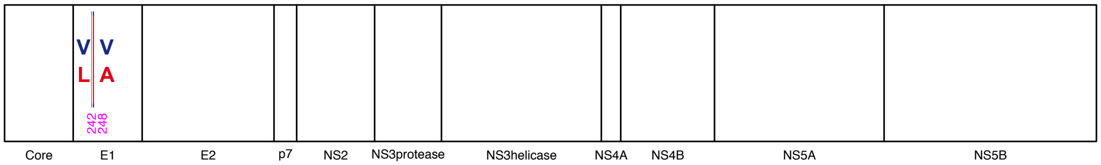

Cluster 48. Hits = 2; distribution of residues = 21/6; p = 1.1e-4

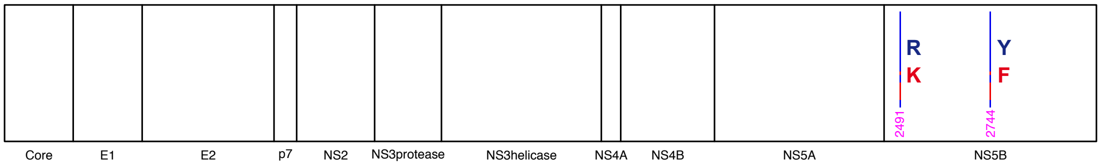

Cluster 50. Hits = 2; distribution of residues = 25/2; p = 7.5e-3

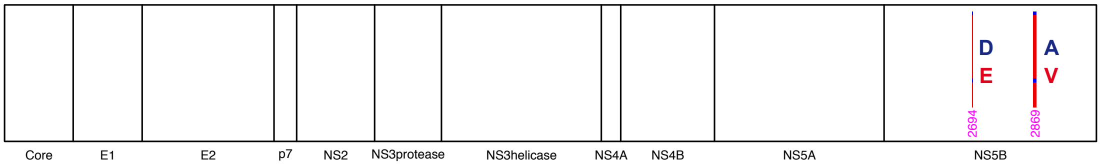

Cluster 51. Hits = 2; distribution of residues = 24/2/1; p = 6.8e-4

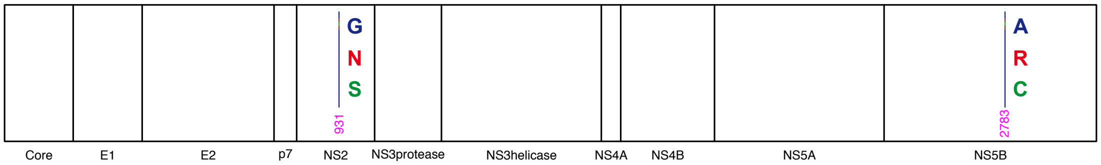

Cluster 52. Hits = 2; distribution of residues = 24/3; p = 1.7e-3

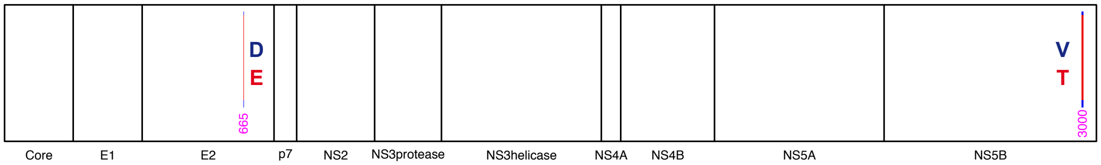

Cluster 53. Hits = 2; distribution of residues = 23/4; p = 5.3e-4

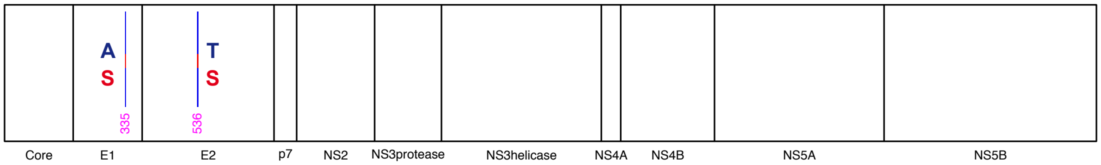

Cluster 55. Hits = 2; distribution of residues = 23/4; p = 5.3e-4

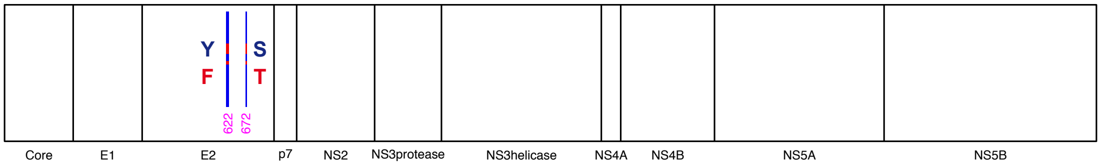

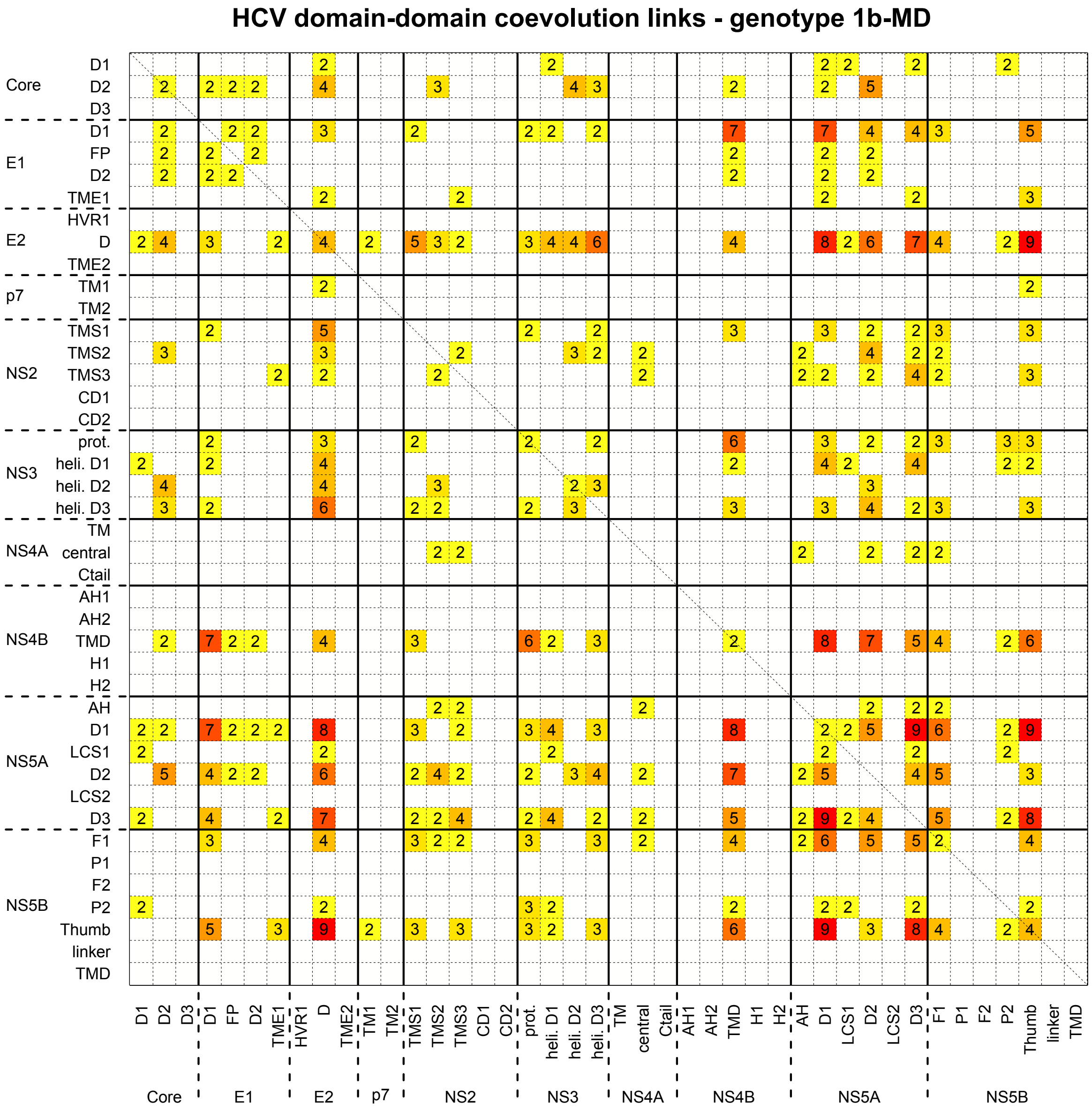

Supplementary Figure 4. Matrix describing HCV domain-domain coevolution links in genotype 1b-MD.

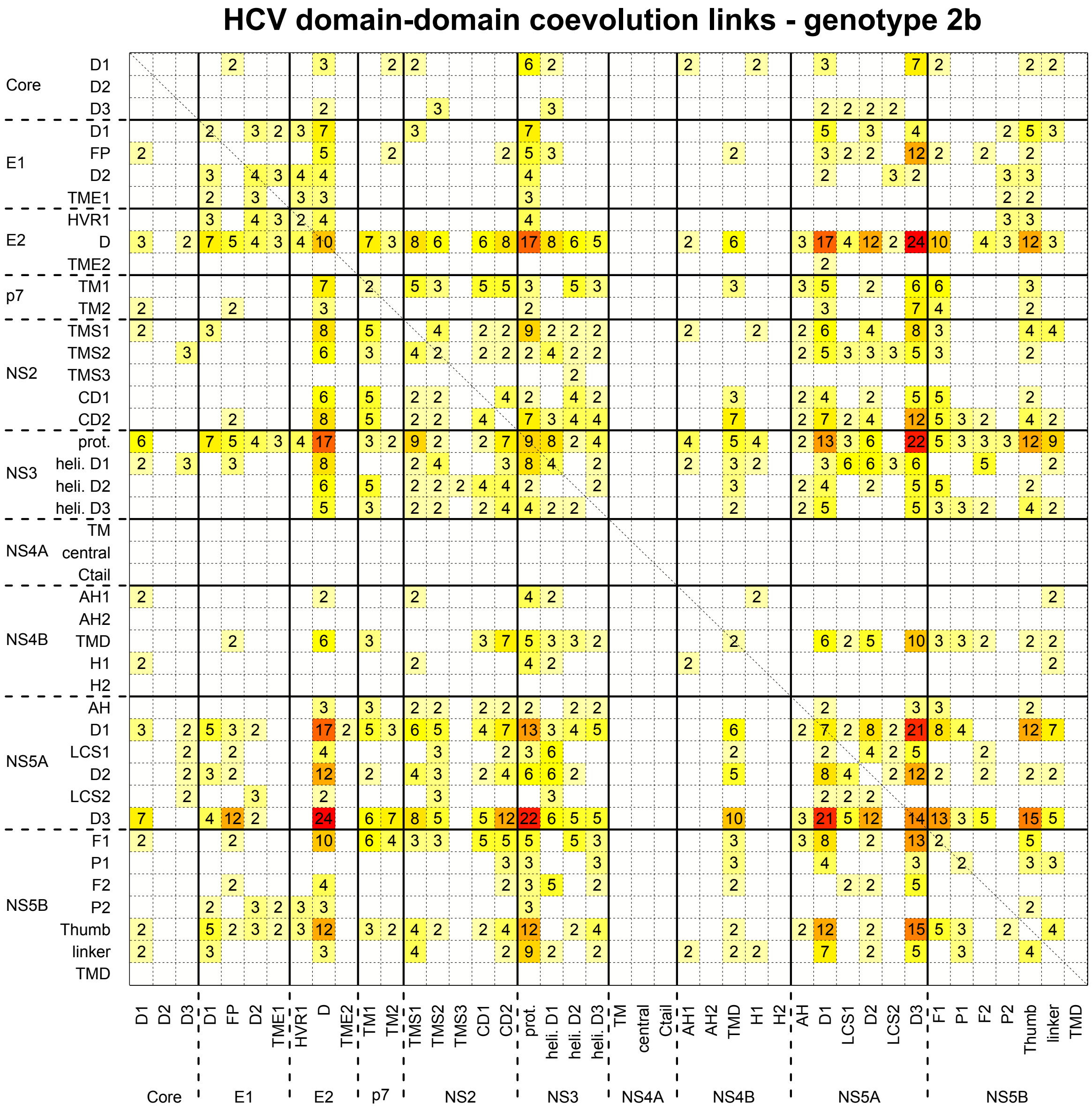

Supplementary Figure 5. Matrix describing HCV domain-domain coevolution links in genotype 2b.

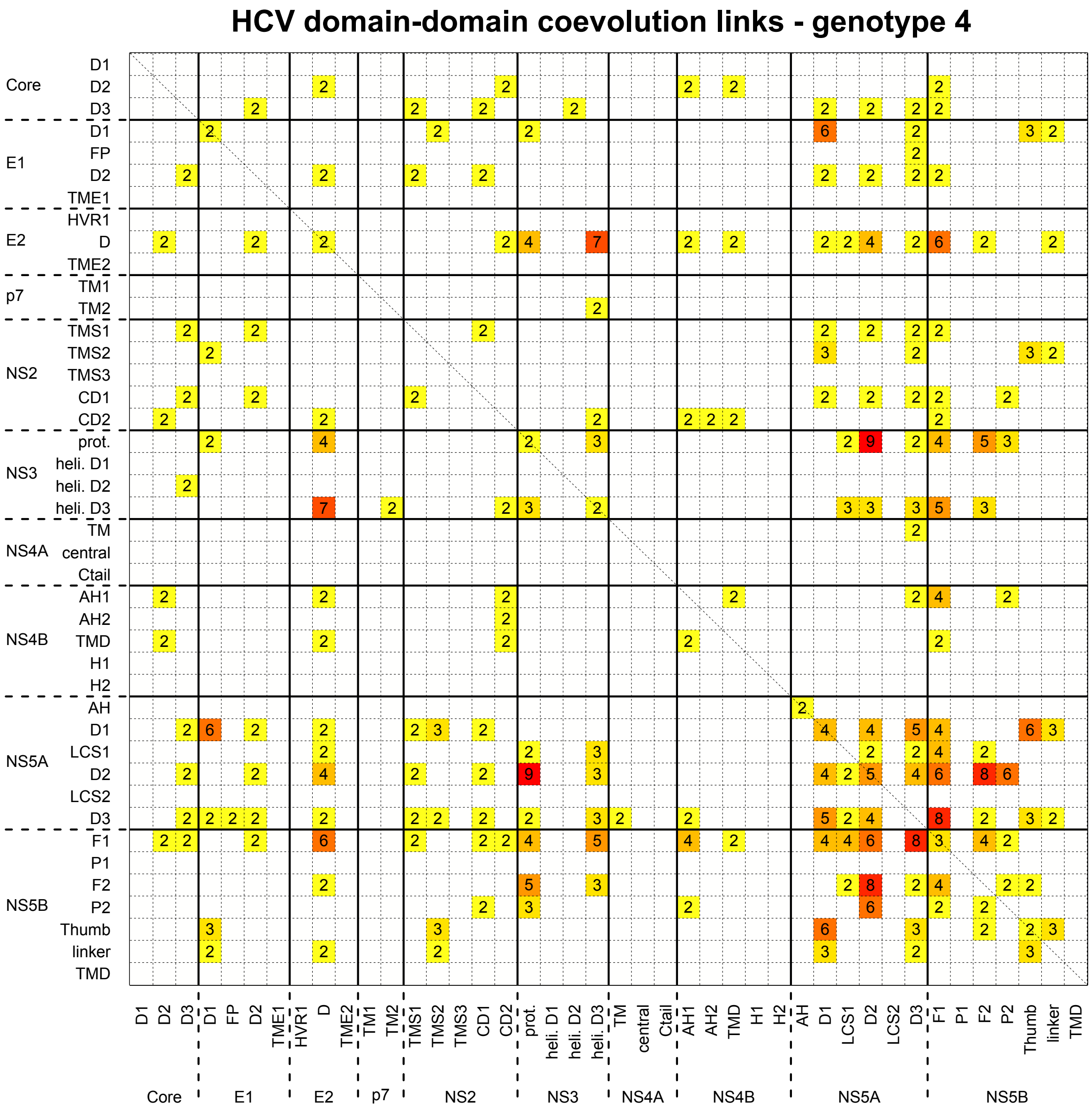

Supplementary Figure 6. Matrix describing HCV domain-domain coevolution links in genotype 4.

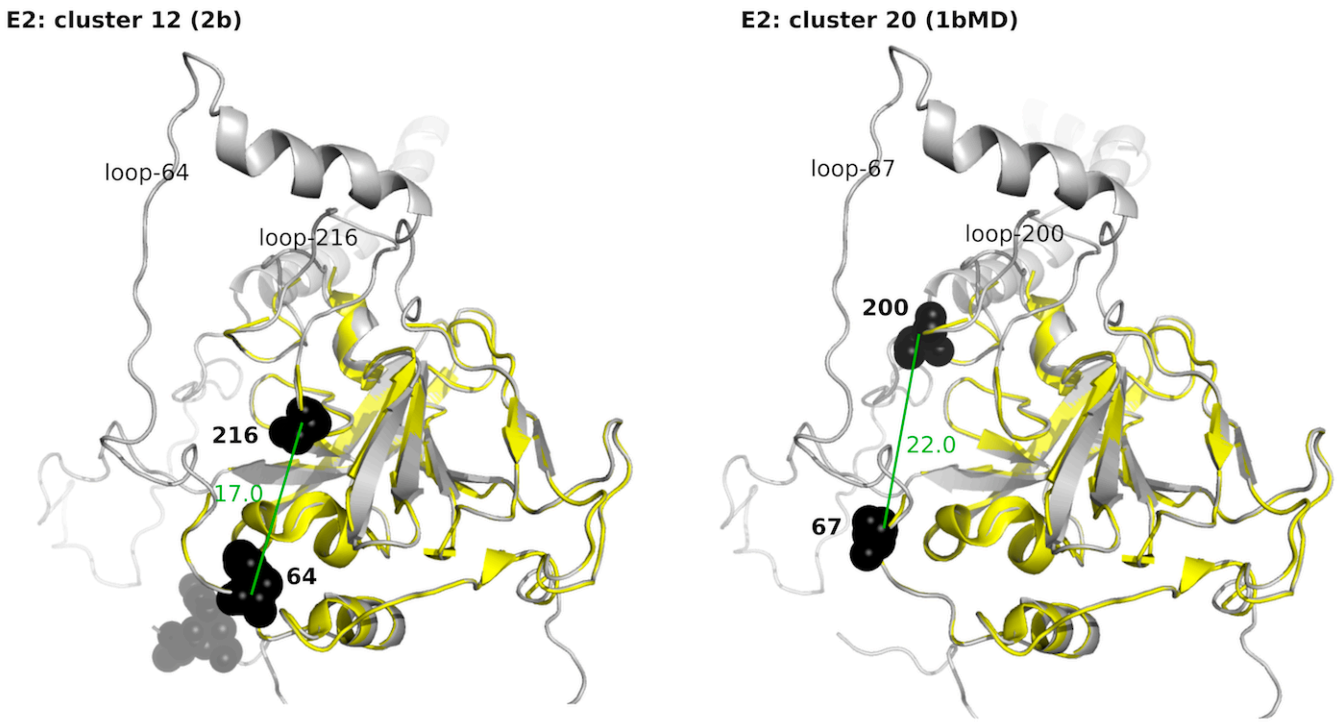

Supplementary Figure 7. Coevolving residues located in the structure of the E2 protein and highlighting intra-molecular interactions. In each structure, black residues form a cluster of coevolving residues. The distance between their C_α atoms is indicated in the figures. Observe the proximity of the two pairs of residues identified as pairwise coevolving (they form two distinct coevolution clusters).

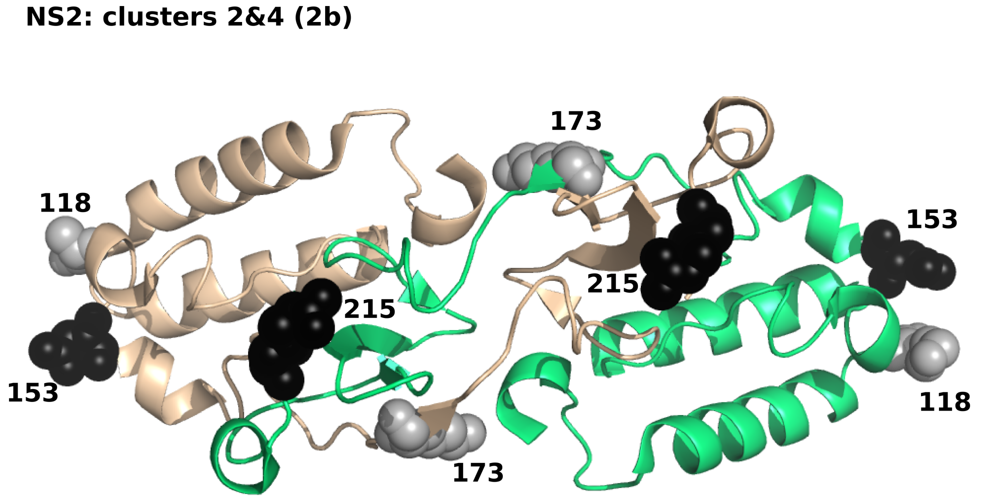

Supplementary Figure 8. Coevolving residues located in the structure of the NS2 protein and highlighting intra-molecular interactions. NS2 forms a dimer whose structure is represented by the position of the two beige and green NS2 copies. Two clusters of pairs of coevolving residues, indicated in grey and black respectively, are reported in the two structures. They are proximal by grey-black pairs with a C_α atoms distance < 21Å: 118-153 and 173-215.

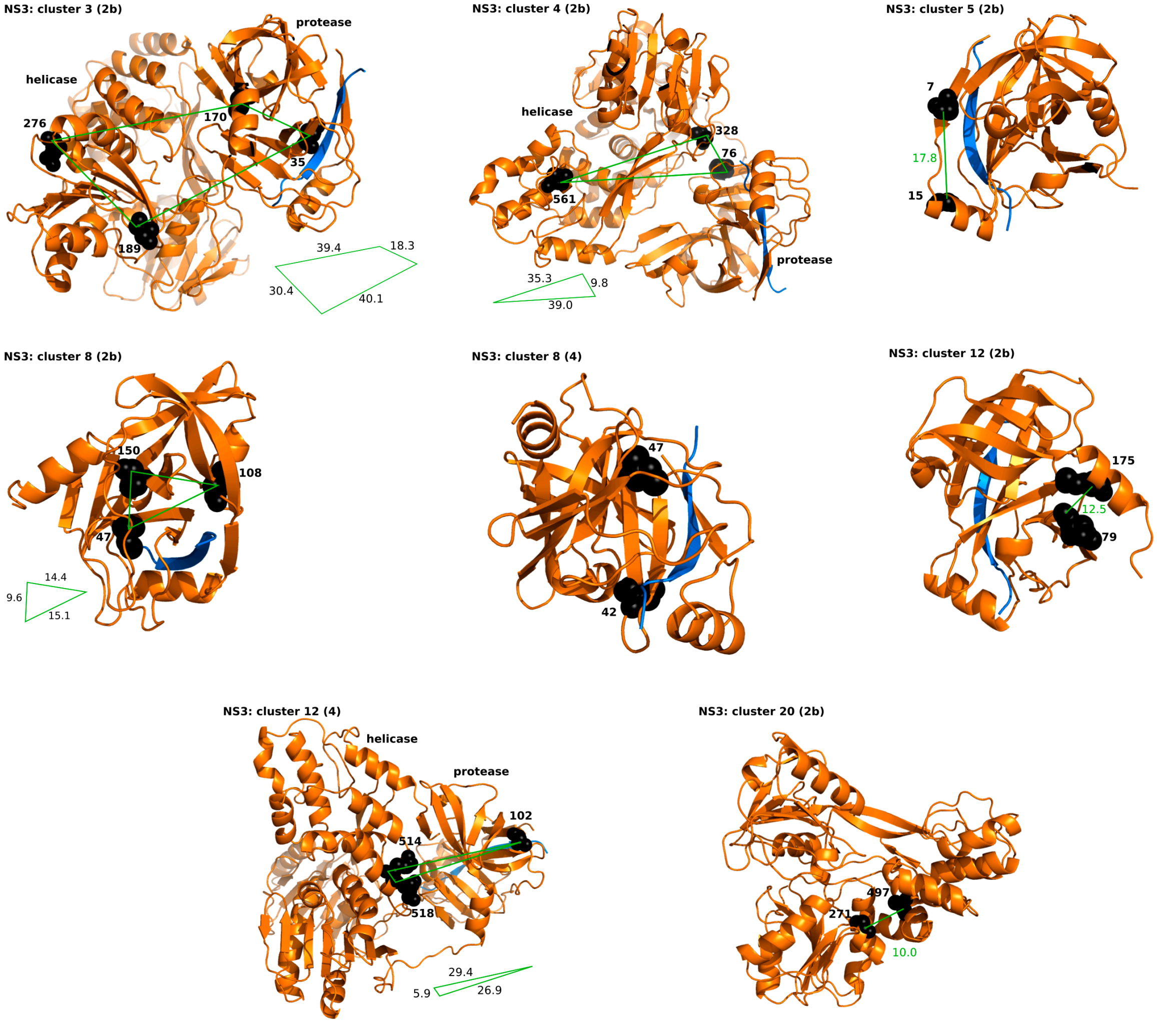

Supplementary Figure 9. Coevolving residues forming clusters located in the structure of the NS3 protein and highlighting intra-molecular interactions. The distance between their C_α atoms is indicated in the figures.

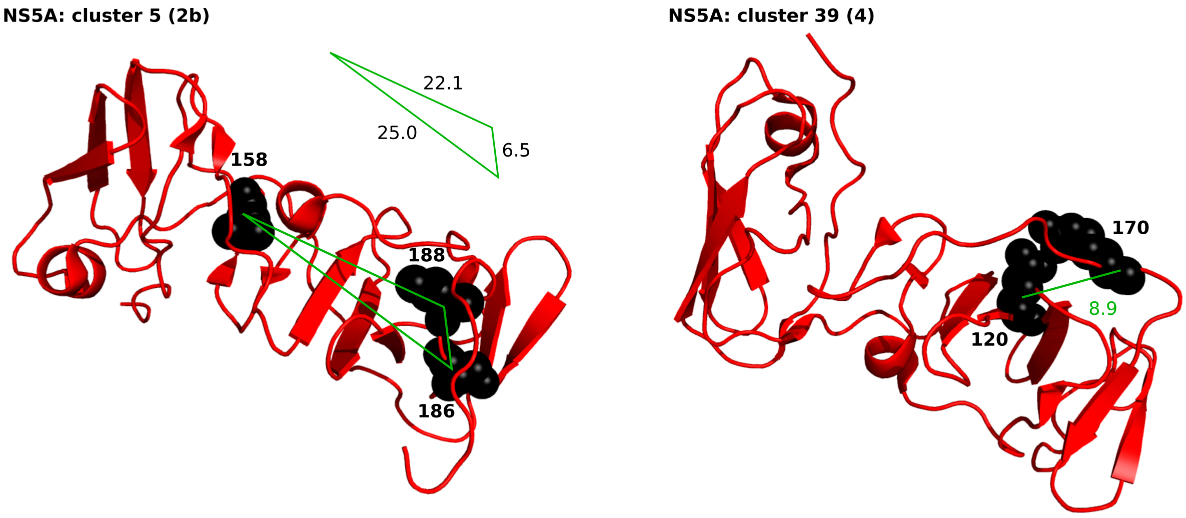

Supplementary Figure 10. Coevolving residues forming clusters located in the structure of the NS5A protein and highlighting intra-molecular interactions. The distance between their C_α atoms is indicated in the figures.

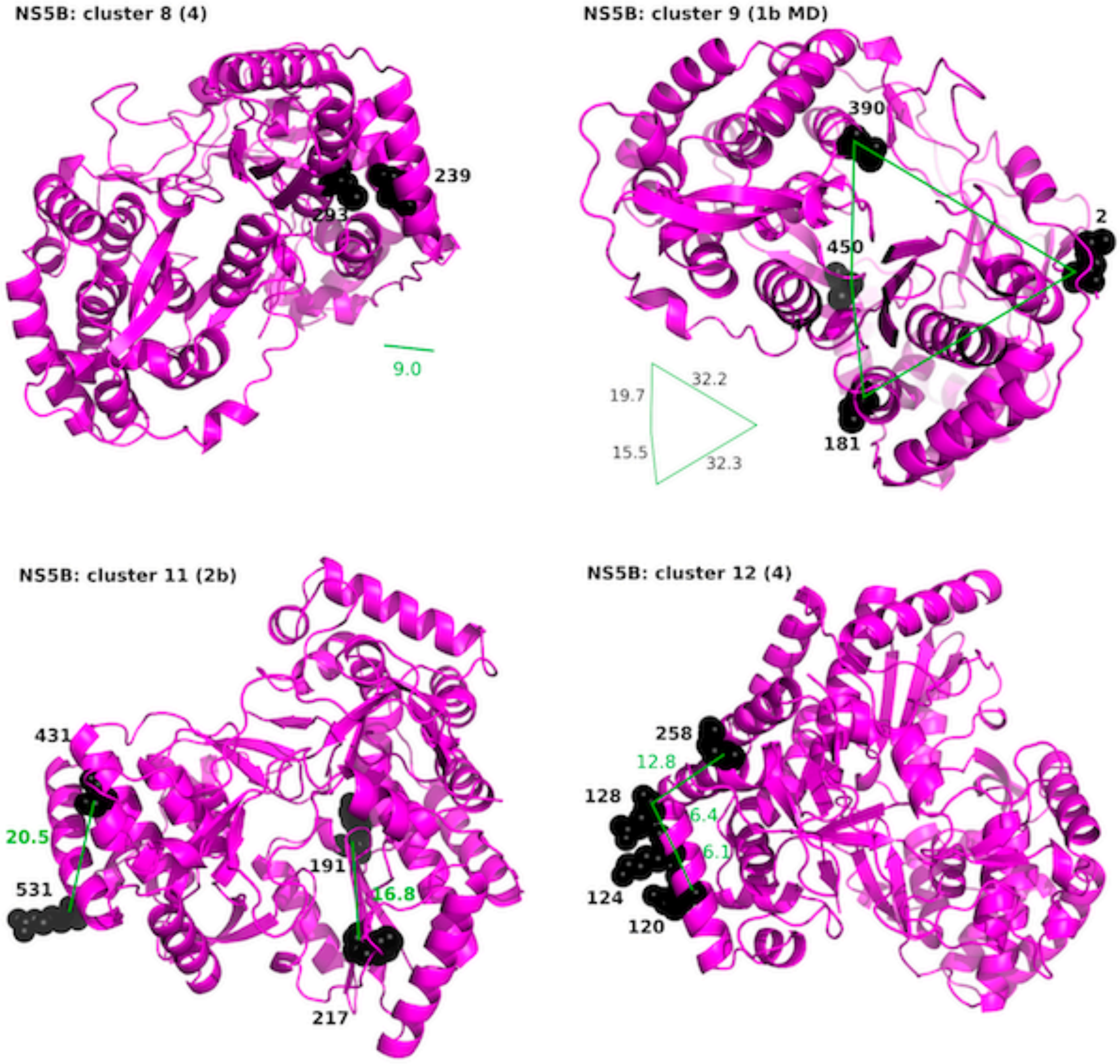

Supplementary Figure 11. Coevolving residues forming clusters located in the structure of the NS5B protein and highlighting intra-molecular interactions. The distance between their C_α atoms is indicated in the figures. Cluster 12 (4) is also plotted in Figure 8; we argued that, with high probability, the NS5B site is in interaction with NS3 (see Supplementary Figure 10 and Figure 8), displaying three other coevolving residues belonging to the same cluster.

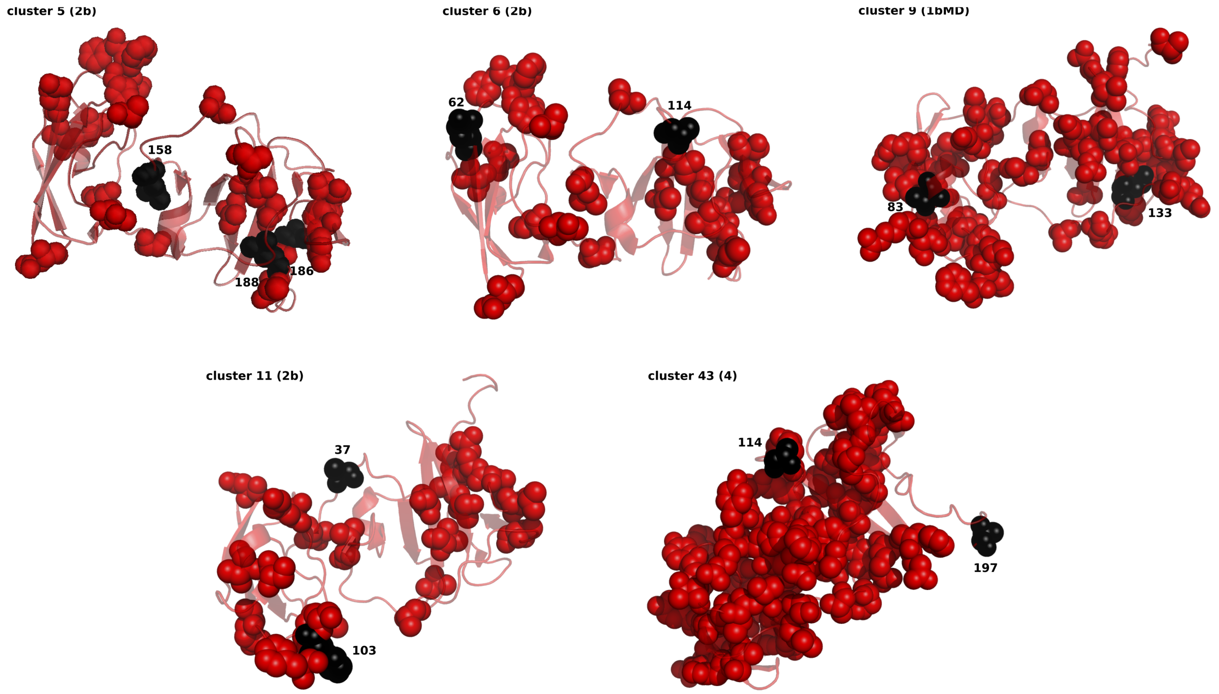

Supplementary Figure 12. Clusters of coevolving residues are represented in black on the structure of the NS5A protein. The name of the cluster and the genotype (in parenthesis) are reported for each structure. Compare to Figure 9. Residues that are not 100% conserved in the sequence alignment associated to the genotype are represented in red atomic balls. In contrast, the transparent cartoon indicates all residues in the protein that are 100% conserved. Note that for genotypes 1b-MD, 2b and 4 we have 113, 139 and 92 fully conserved (100%) residues respectively, with a total of 69 residues that appear in all the three genotypes as 100% conserved. If we consider also those positions that are completely conserved with the exception of one sequence, we obtain 132, 145 and 118 conserved residues excluded by the analysis for the three genotypes and a total of 93 residues that are common to all genotypes and conserved. This means that BIS^2 analysis of NS5A concerned only 31, 18 and 45 residues for genotype 1b-MD, 2b and 4.

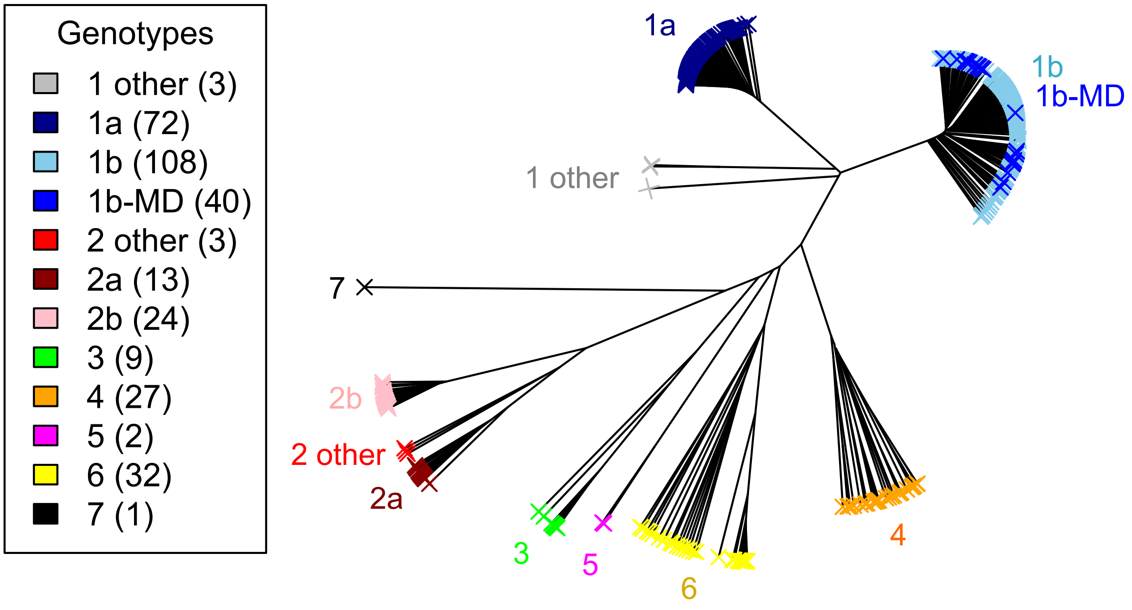

Supplementary Figure 13. Distance tree of non-redundant available full-length HCV polyprotein sequences. HCV sequences are structures in 7 genotypes defined by sequence divergence. The associated distance tree shows that genotypes are clearly distinguished by sequence divergence and that within a genotype, some sub-genotypes might be distinguished further (they correspond to subtrees in the tree). The number of sequences for each genotype is reported (in parenthesis) in the legend.

Supplementary Figure 14:

Trees associated to clusters described in Supplementary Figures 1-3

(Leaves in a tree are labelled with the hits of the cluster)

HCV_1b_MD_mafft BIS_dim1_cluster6_sym100_env100 #hits=4 distribution=36/4 p=1.1e−05

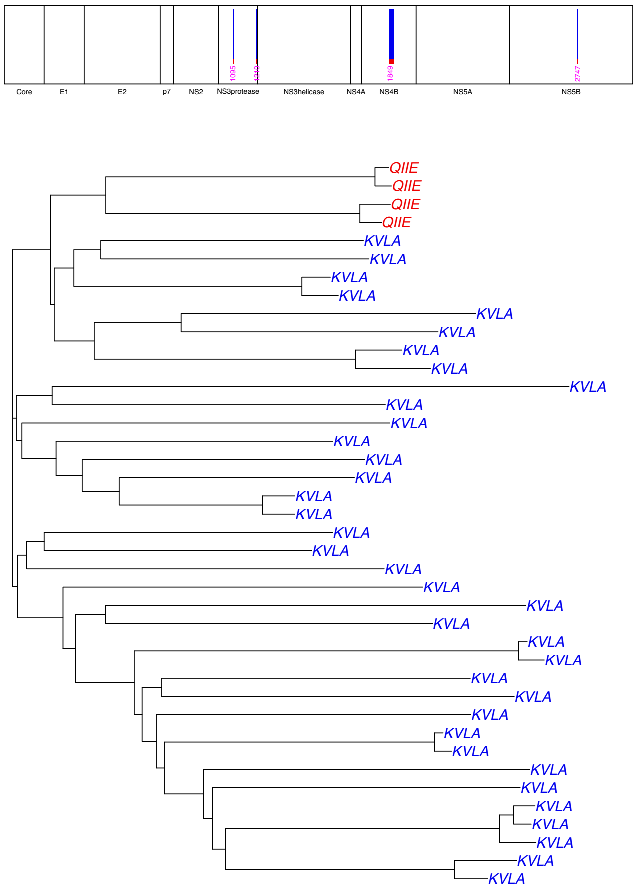

HCV_1b_MD_mafft BIS_dim1_cluster9_sym100_env100 #hits=16 distribution=38/2 p=0.0013

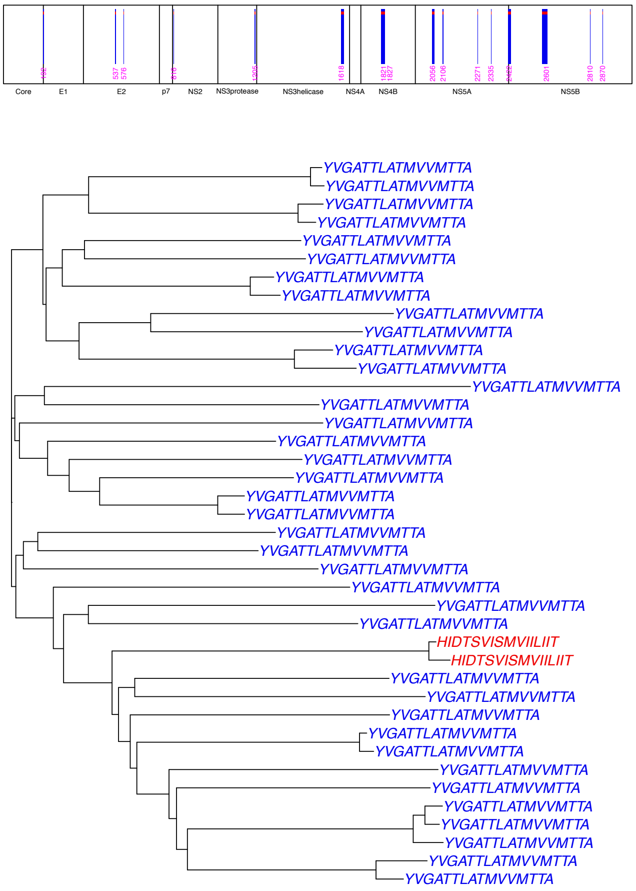

HCV_1b_MD_mafft BIS_dim1_cluster11_sym100_env100 #hits=6 distribution=38/2 p=0.0013

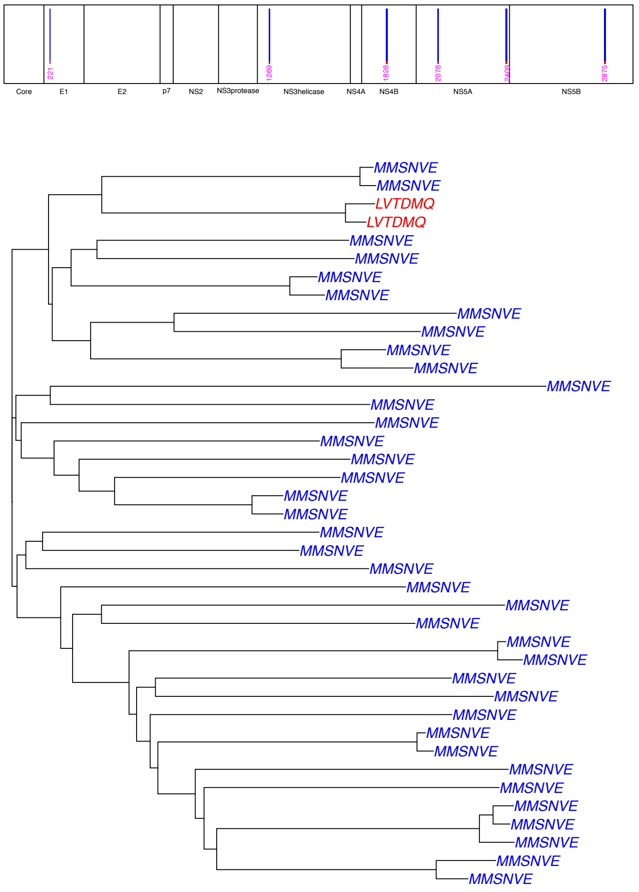

HCV_1b_MD_mafft BIS_dim1_cluster13_sym100_env100 #hits=7 distribution=38/2 p=0.0013

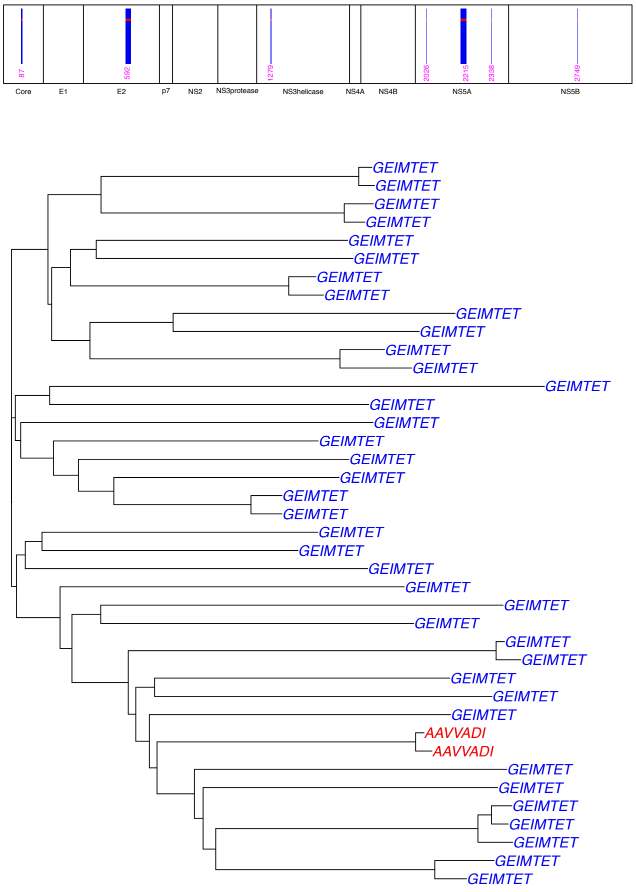

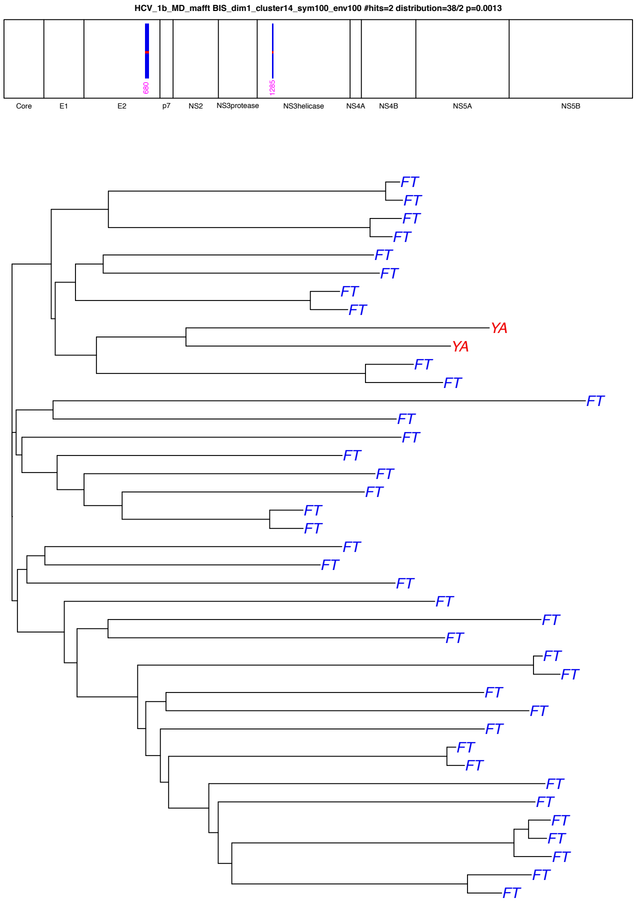
HCV_1b_MD_mafft BIS_dim1_cluster14_sym100_env100 #hits=2 distribution=38/2 p=0.0013
Core
E1
E2
p7
NS2
NS3protease
NS3helicase
NS4A
NS4B
NS5A
NS5B
FT
YA

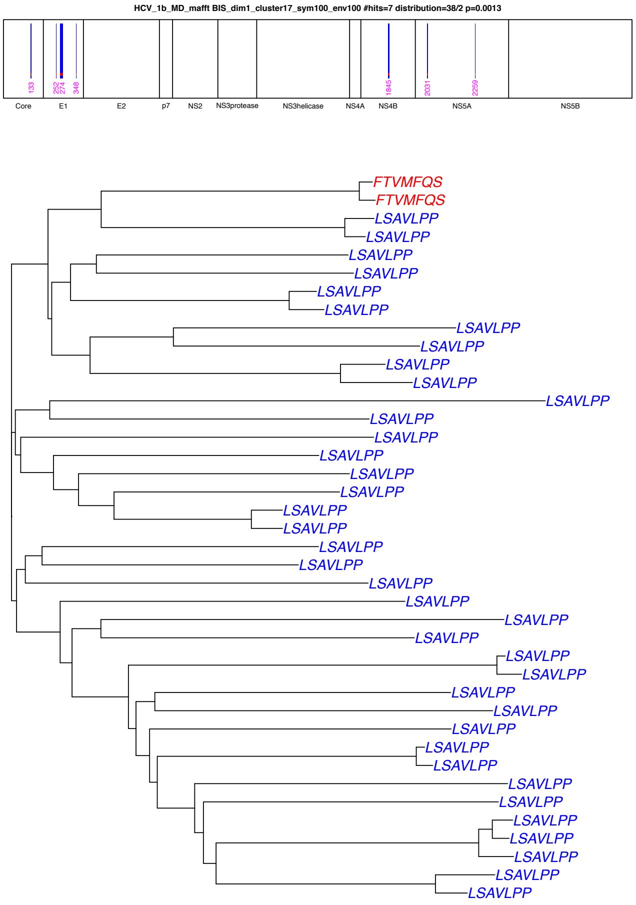
HCV_1b_MD_mafft BIS_dim1_cluster17_sym100_env100 #hits=7 distribution=38/2 p=0.0013
Core
E1
E2
p7
NS2
NS3protease
NS3helicase
NS4A
NS4B
NS5A
NS5B
FTVMFQS
LSAVLPP

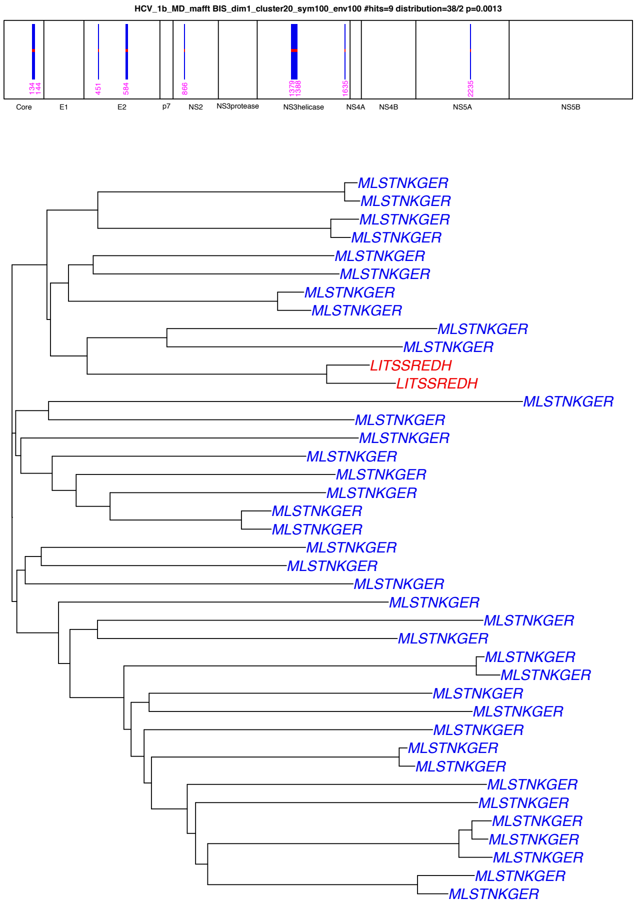
HCV_1b_MD_mafft BIS_dim1_cluster20_sym100_env100 #hits=9 distribution=38/2 p=0.0013
Core
E1
E2
p7
NS2
NS3protease
NS3helicase
NS4A
NS4B
NS5A
NS5B
MLSTNKGER
LITSSREDH

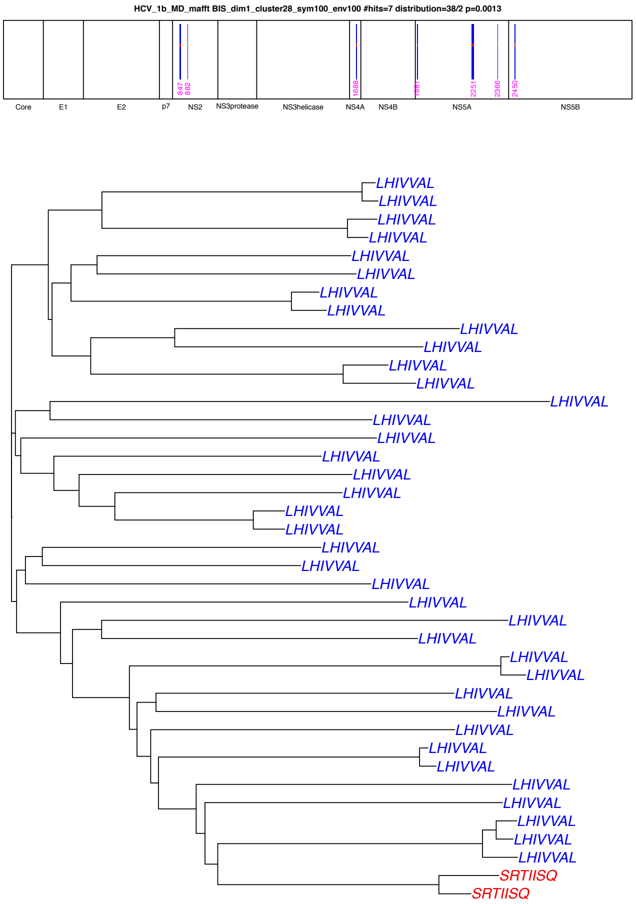
HCV_1b_MD_mafft BIS_dim1_cluster28_sym100_env100 #hits=7 distribution=38/2 p=0.0013
Core
E1
E2
p7
NS2
NS3protease
NS3helicase
NS4A
NS4B
NS5A
NS5B
LHIVVAL
SRTIISQ

HCV_1b_MD_mafft BIS_dim1_cluster29_sym100_env100 #hits=2 distribution=38/2 p=0.0013

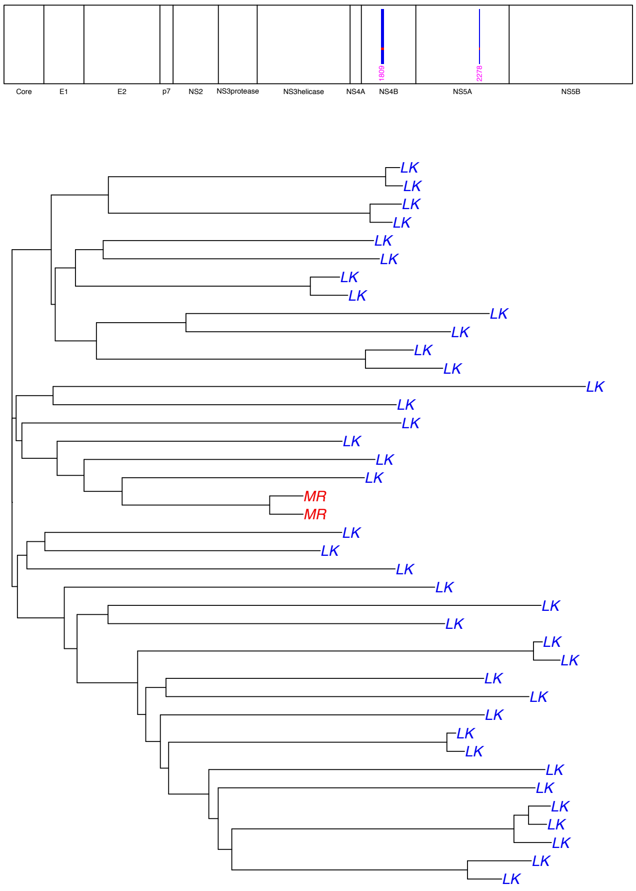

HCV_1b_MD_mafft BIS_dim1_cluster32_sym100_env100 #hits=7 distribution=37/3 p=1e−04

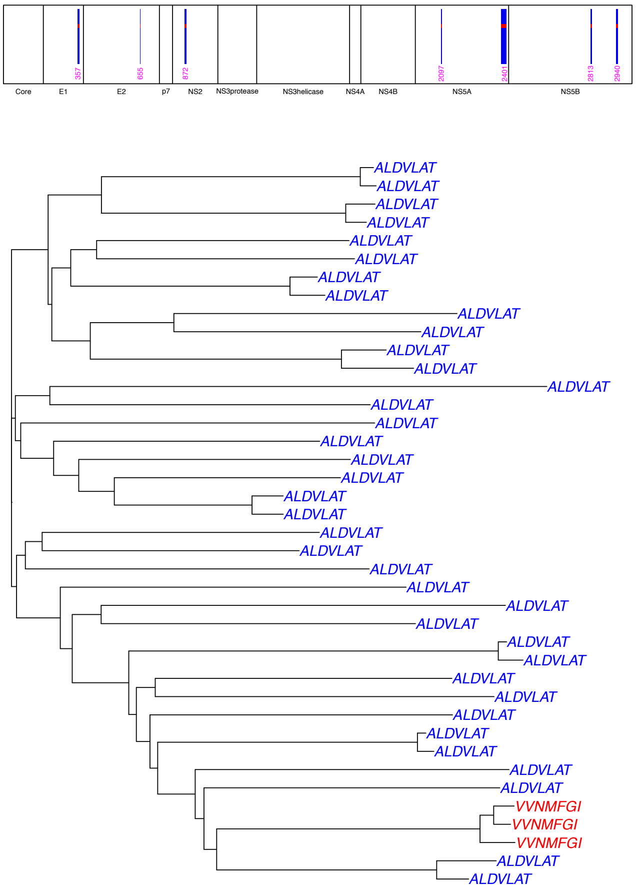

HCV_1b_MD_mafft BIS_dim1_cluster36_sym100_env100 #hits=2 distribution=38/2 p=0.0013

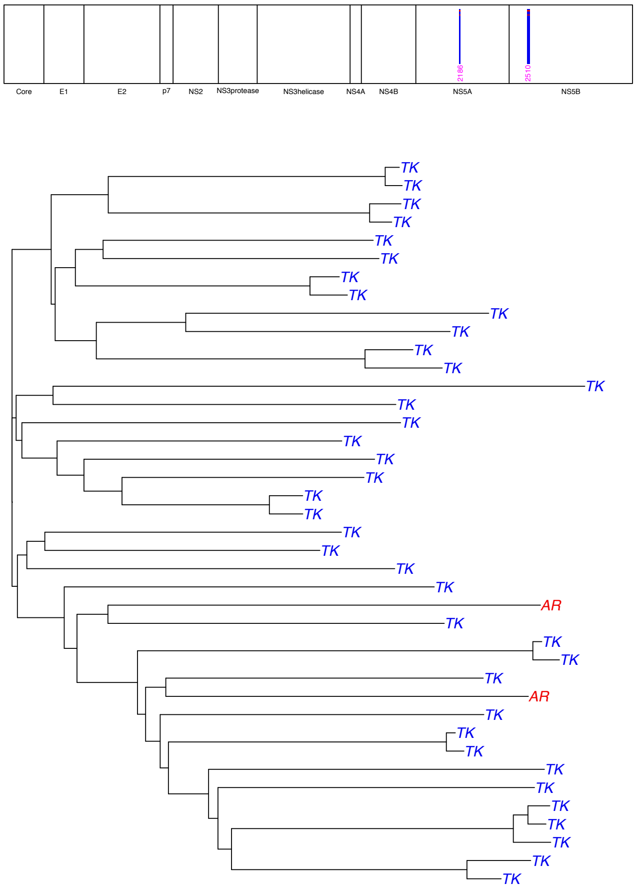

HCV_1b_MD_mafft BIS_dim1_cluster39_sym100_env100 #hits=2 distribution=23/17 p=1.1e−11

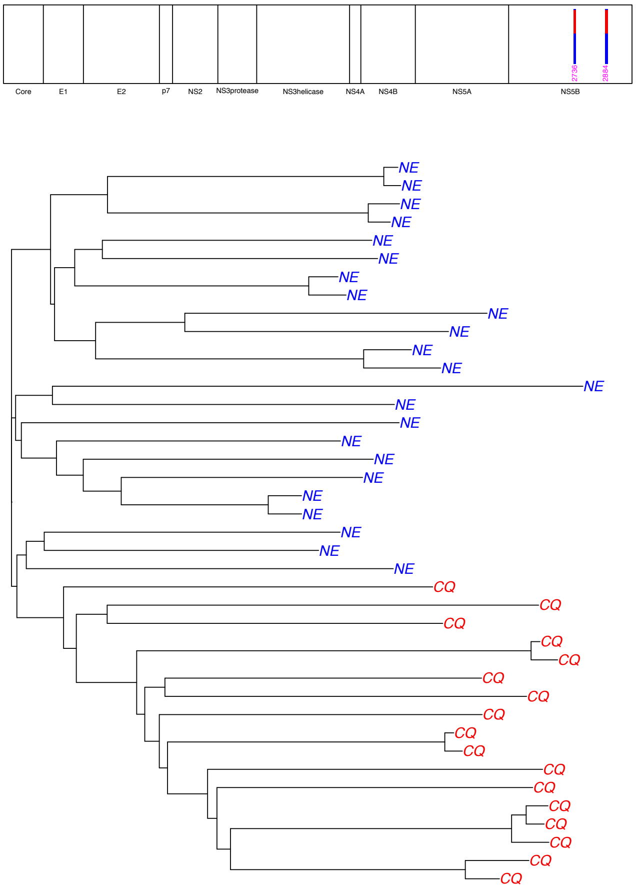

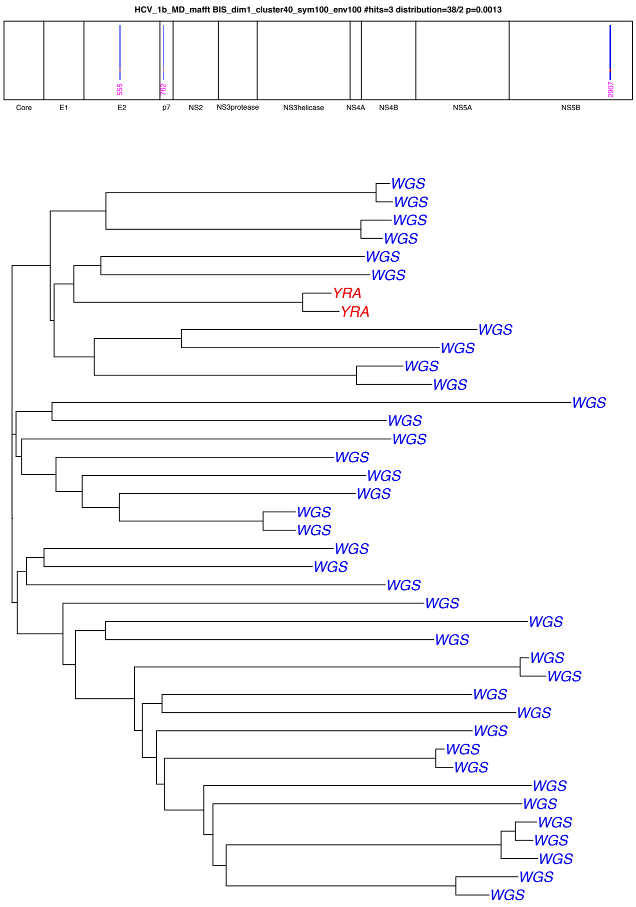
HCV_1b_MD_mafft BIS_dim1_cluster40_sym100_env100 #hits=3 distribution=38/2 p=0.0013
Core
E1
E2
p7
NS2
NS3protease
NS3helicase
NS4A
NS4B
NS5A
NS5B

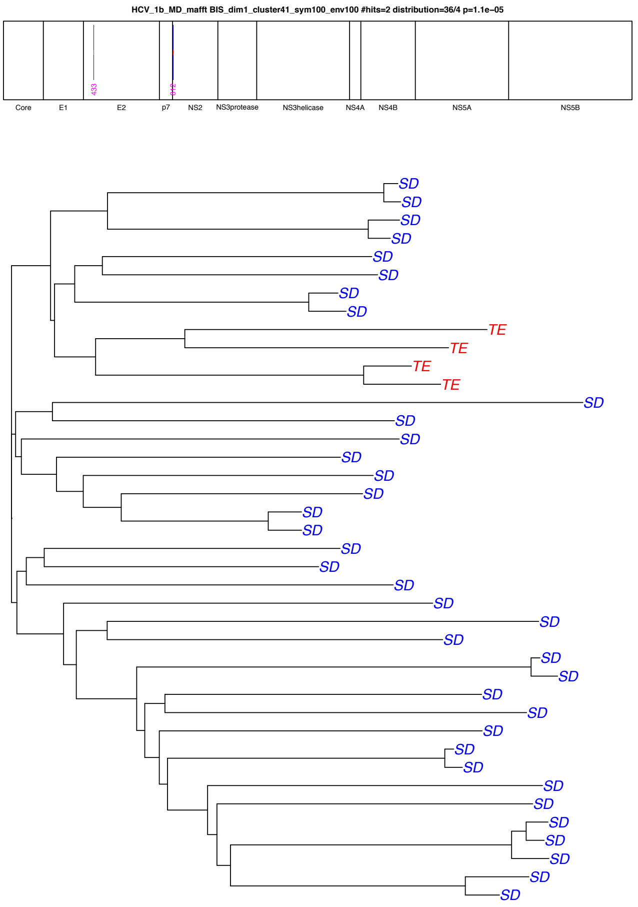
HCV_1b_MD_mafft BIS_dim1_cluster41_sym100_env100 #hits=2 distribution=36/4 p=1.1e−05
Core
E1
E2
p7
NS2
NS3protease
NS3helicase
NS4A
NS4B
NS5A
NS5B

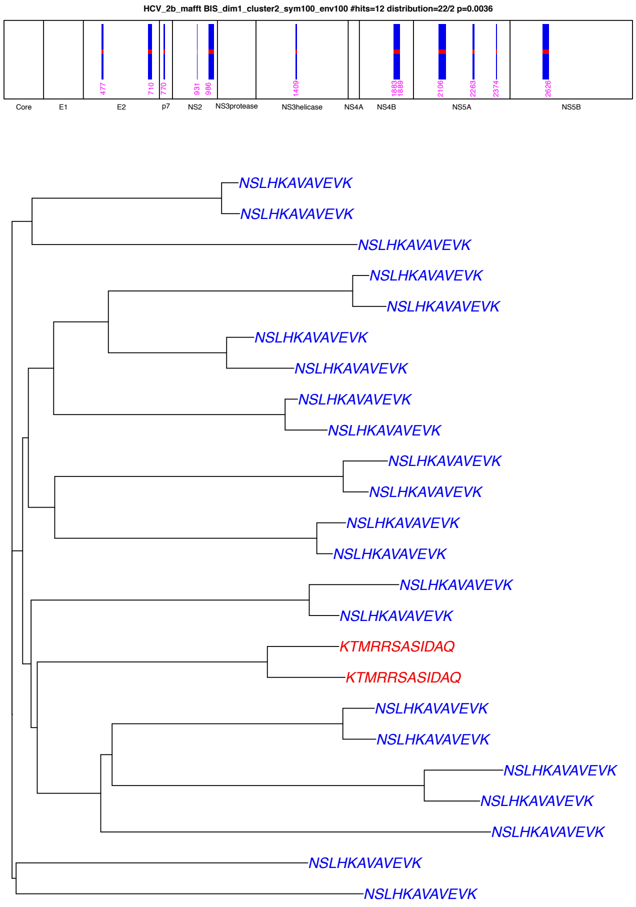
HCV_2b_mafft BIS_dim1_cluster2_sym100_env100 #hits=12 distribution=22/2 p=0.0036
Core
E1
E2
p7
NS2
NS3protease
NS3helicase
NS4A
NS4B
NS5A
NS5B

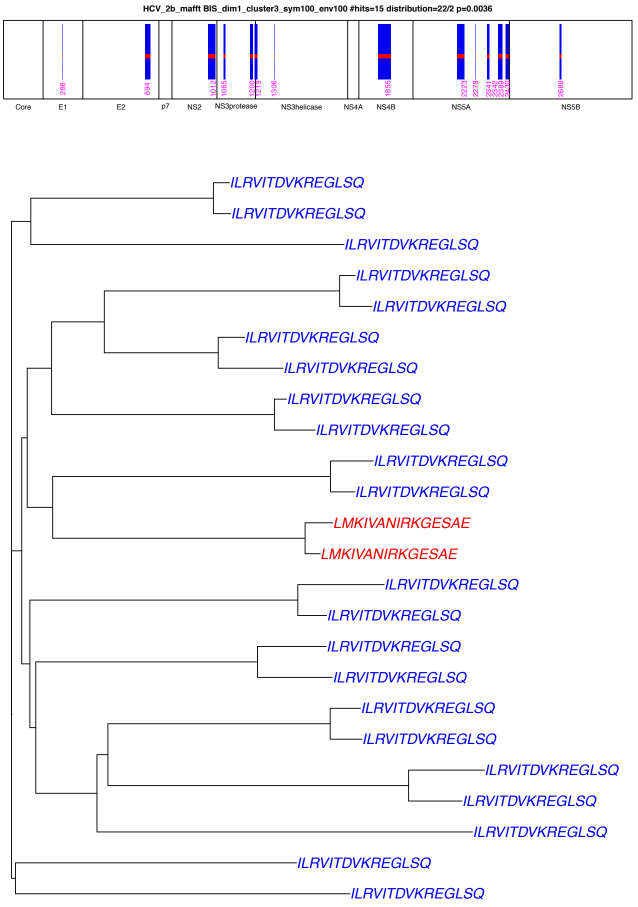
HCV_2b_mafft BIS_dim1_cluster3_sym100_env100 #hits=15 distribution=22/2 p=0.0036
Core
E1
E2
p7
NS2
NS3protease
NS3helicase
NS4A
NS4B
NS5A
NS5B

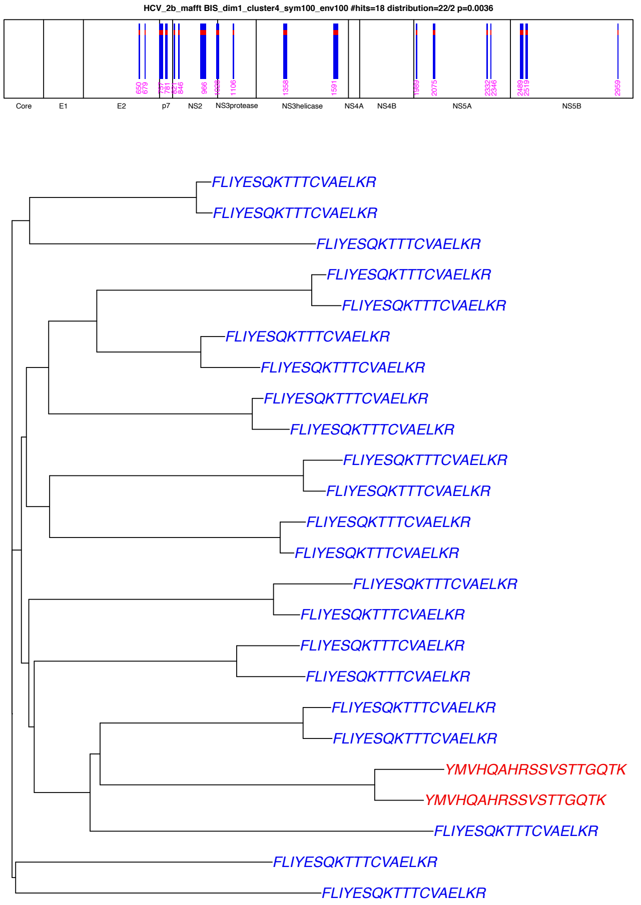
HCV_2b_mafft BIS_dim1_cluster4_sym100_env100 #hits=18 distribution=22/2 p=0.0036
Core
E1
E2
p7
NS2
NS3protease
NS3helicase
NS4A
NS4B
NS5A
NS5B
FLIYESQKTTTCVAELKR
FLIYESQKTTTCVAELKR
FLIYESQKTTTCVAELKR
FLIYESQKTTTCVAELKR
FLIYESQKTTTCVAELKR
FLIYESQKTTTCVAELKR
FLIYESQKTTTCVAELKR
FLIYESQKTTTCVAELKR
FLIYESQKTTTCVAELKR
FLIYESQKTTTCVAELKR
FLIYESQKTTTCVAELKR
FLIYESQKTTTCVAELKR
FLIYESQKTTTCVAELKR
FLIYESQKTTTCVAELKR
FLIYESQKTTTCVAELKR
FLIYESQKTTTCVAELKR
FLIYESQKTTTCVAELKR
FLIYESQKTTTCVAELKR
FLIYESQKTTTCVAELKR
YMVHQAHRSSVSTTGQTK
YMVHQAHRSSVSTTGQTK
FLIYESQKTTTCVAELKR
FLIYESQKTTTCVAELKR
FLIYESQKTTTCVAELKR

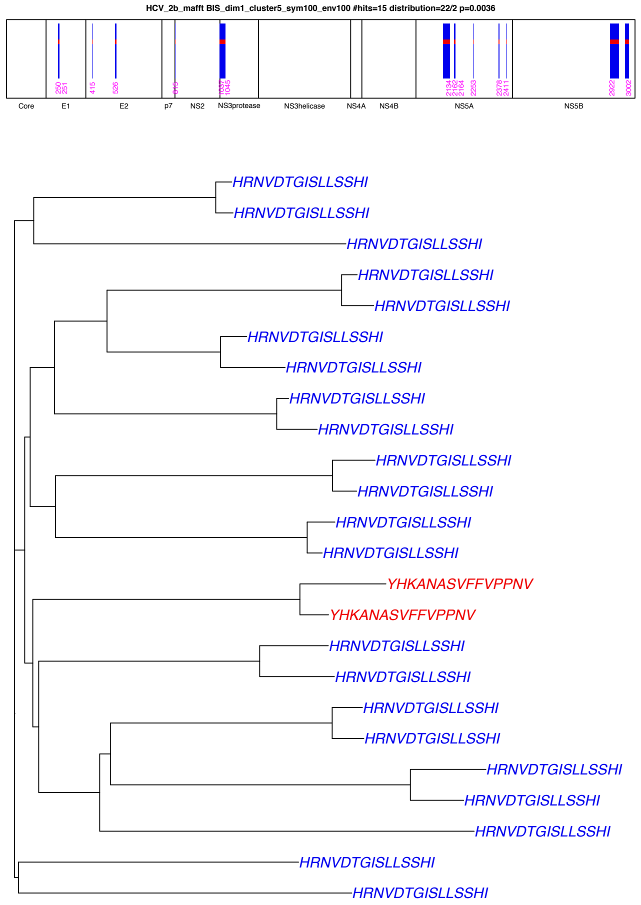
HCV_2b_mafft BIS_dim1_cluster5_sym100_env100 #hits=15 distribution=22/2 p=0.0036
Core
E1
E2
p7
NS2
NS3protease
NS3helicase
NS4A
NS4B
NS5A
NS5B
HRNVDTGISLLSSHI
HRNVDTGISLLSSHI
HRNVDTGISLLSSHI
HRNVDTGISLLSSHI
HRNVDTGISLLSSHI
HRNVDTGISLLSSHI
HRNVDTGISLLSSHI
HRNVDTGISLLSSHI
HRNVDTGISLLSSHI
HRNVDTGISLLSSHI
HRNVDTGISLLSSHI
HRNVDTGISLLSSHI
HRNVDTGISLLSSHI
YHKANASVFFVPPNV
YHKANASVFFVPPNV
HRNVDTGISLLSSHI
HRNVDTGISLLSSHI
HRNVDTGISLLSSHI
HRNVDTGISLLSSHI
HRNVDTGISLLSSHI
HRNVDTGISLLSSHI
HRNVDTGISLLSSHI
HRNVDTGISLLSSHI
HRNVDTGISLLSSHI

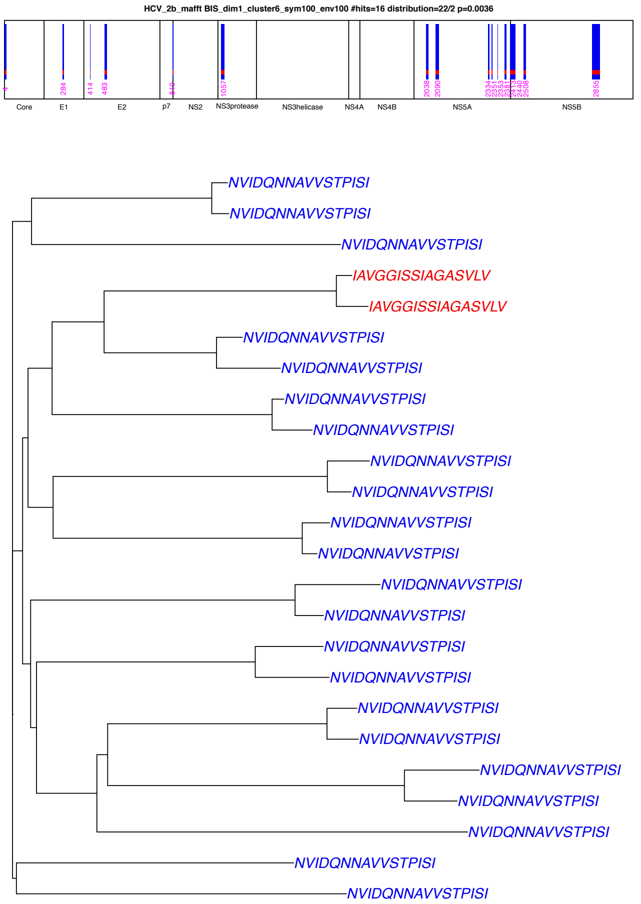
HCV_2b_mafft BIS_dim1_cluster6_sym100_env100 #hits=16 distribution=22/2 p=0.0036
Core
E1
E2
p7
NS2
NS3protease
NS3helicase
NS4A
NS4B
NS5A
NS5B
NVIDQNNAVVSTPISI
NVIDQNNAVVSTPISI
NVIDQNNAVVSTPISI
IAVGGISSIAGASVLV
IAVGGISSIAGASVLV
NVIDQNNAVVSTPISI
NVIDQNNAVVSTPISI
NVIDQNNAVVSTPISI
NVIDQNNAVVSTPISI
NVIDQNNAVVSTPISI
NVIDQNNAVVSTPISI
NVIDQNNAVVSTPISI
NVIDQNNAVVSTPISI
NVIDQNNAVVSTPISI
NVIDQNNAVVSTPISI
NVIDQNNAVVSTPISI
NVIDQNNAVVSTPISI
NVIDQNNAVVSTPISI
NVIDQNNAVVSTPISI
NVIDQNNAVVSTPISI
NVIDQNNAVVSTPISI
NVIDQNNAVVSTPISI
NVIDQNNAVVSTPISI
NVIDQNNAVVSTPISI

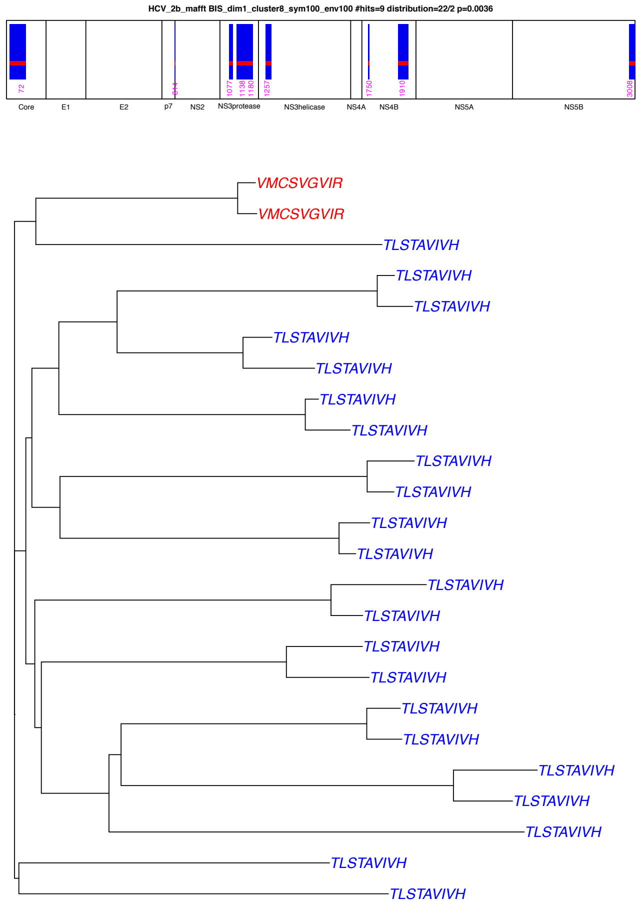
HCV_2b_mafft BIS_dim1_cluster8_sym100_env100 #hits=9 distribution=22/2 p=0.0036
Core
E1
E2
p7
NS2
NS3protease
NS3helicase
NS4A
NS4B
NS5A
NS5B
VMCSVGVIR
VMCSVGVIR
TLSTAVIVH
TLSTAVIVH
TLSTAVIVH
TLSTAVIVH
TLSTAVIVH
TLSTAVIVH
TLSTAVIVH
TLSTAVIVH
TLSTAVIVH
TLSTAVIVH
TLSTAVIVH
TLSTAVIVH
TLSTAVIVH
TLSTAVIVH
TLSTAVIVH
TLSTAVIVH
TLSTAVIVH
TLSTAVIVH
TLSTAVIVH
TLSTAVIVH
TLSTAVIVH
TLSTAVIVH

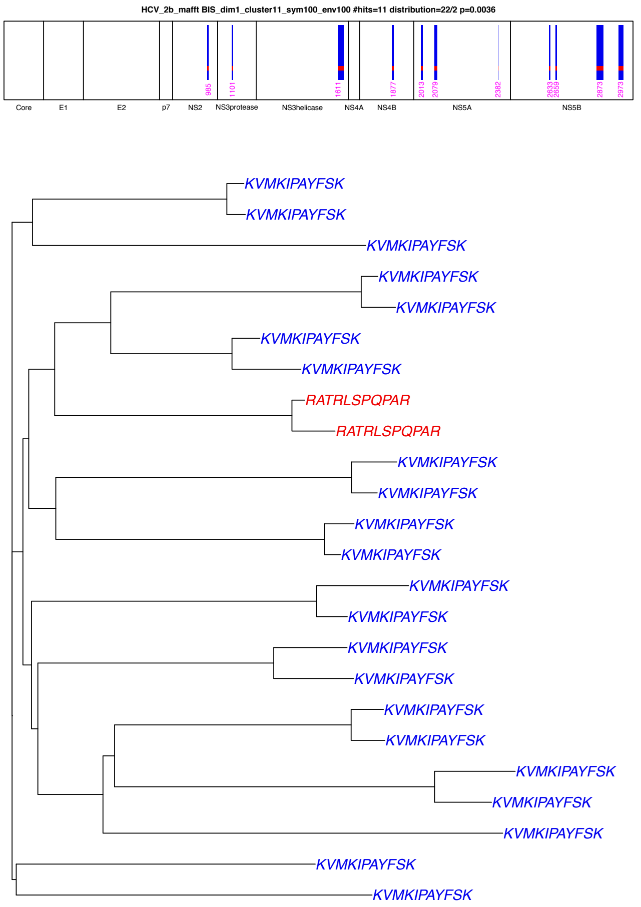
HCV_2b_mafft BIS_dim1_cluster11_sym100_env100 #hits=11 distribution=22/2 p=0.0036
Core
E1
E2
p7
NS2
NS3protease
NS3helicase
NS4A
NS4B
NS5A
NS5B
KVMKIPAYFSK
KVMKIPAYFSK
KVMKIPAYFSK
KVMKIPAYFSK
KVMKIPAYFSK
KVMKIPAYFSK
KVMKIPAYFSK
RATRLSPQPAR
RATRLSPQPAR
KVMKIPAYFSK
KVMKIPAYFSK
KVMKIPAYFSK
KVMKIPAYFSK
KVMKIPAYFSK
KVMKIPAYFSK
KVMKIPAYFSK
KVMKIPAYFSK
KVMKIPAYFSK
KVMKIPAYFSK
KVMKIPAYFSK
KVMKIPAYFSK
KVMKIPAYFSK
KVMKIPAYFSK
KVMKIPAYFSK

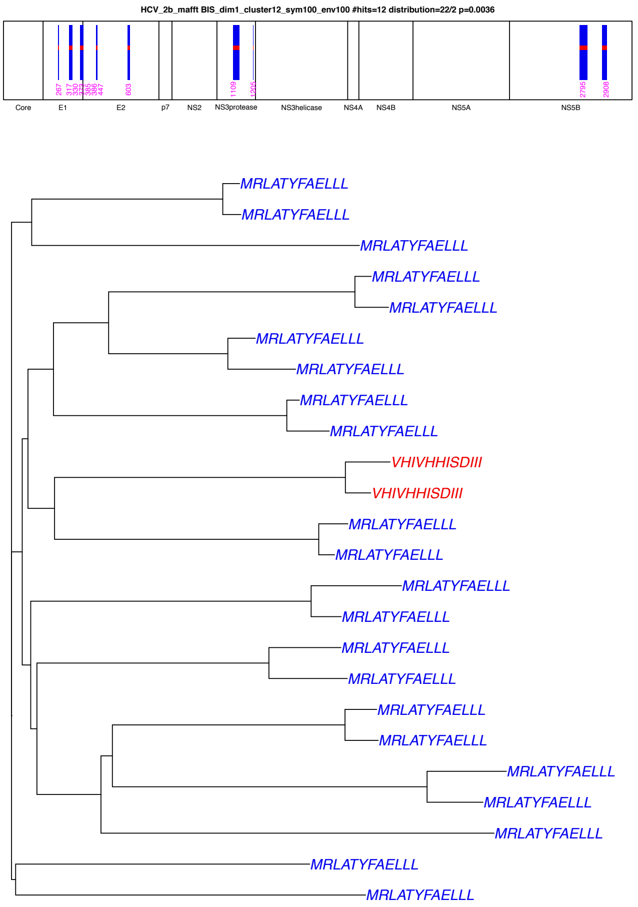
HCV_2b_mafft BIS_dim1_cluster12_sym100_env100 #hits=12 distribution=22/2 p=0.0036
Core
E1
E2
p7
NS2
NS3protease
NS3helicase
NS4A
NS4B
NS5A
NS5B
MRLATYFAELLL
MRLATYFAELLL
MRLATYFAELLL
MRLATYFAELLL
MRLATYFAELLL
MRLATYFAELLL
MRLATYFAELLL
MRLATYFAELLL
MRLATYFAELLL
VHIVHHISDIII
VHIVHHISDIII
MRLATYFAELLL
MRLATYFAELLL
MRLATYFAELLL
MRLATYFAELLL
MRLATYFAELLL
MRLATYFAELLL
MRLATYFAELLL
MRLATYFAELLL
MRLATYFAELLL
MRLATYFAELLL
MRLATYFAELLL
MRLATYFAELLL
MRLATYFAELLL

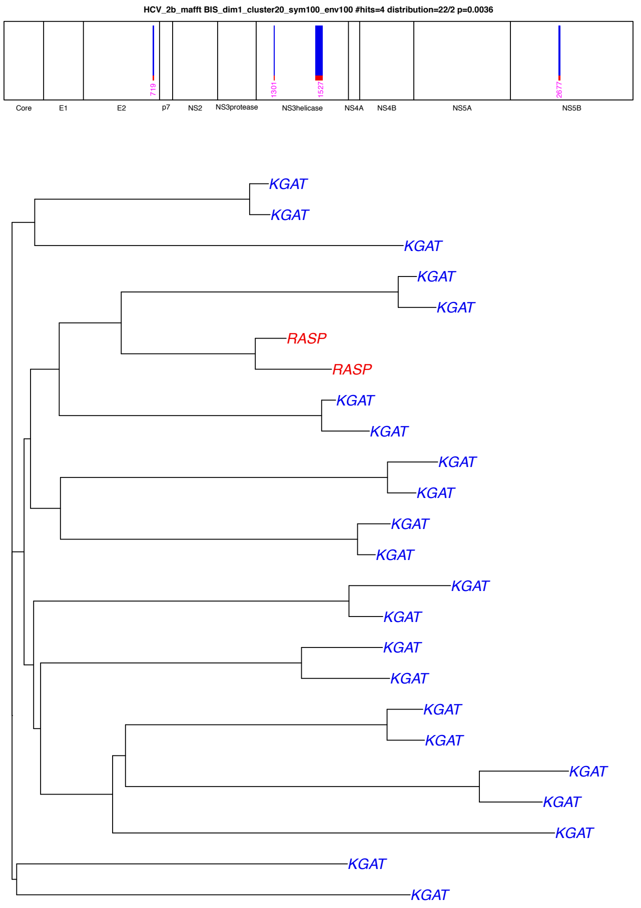
HCV_2b_mafft BIS_dim1_cluster20_sym100_env100 #hits=4 distribution=22/2 p=0.0036
Core
E1
E2
p7
NS2
NS3protease
NS3helicase
NS4A
NS4B
NS5A
NS5B
KGAT
KGAT
KGAT
KGAT
KGAT
RASP
RASP
KGAT
KGAT
KGAT
KGAT
KGAT
KGAT
KGAT
KGAT
KGAT
KGAT
KGAT
KGAT
KGAT
KGAT
KGAT
KGAT
KGAT

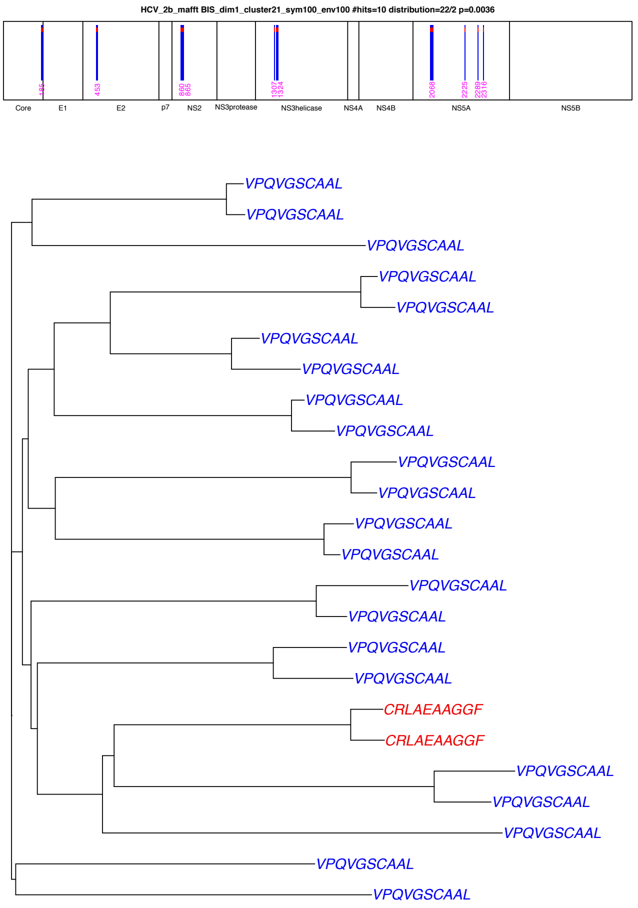
HCV_2b_mafft BIS_dim1_cluster21_sym100_env100 #hits=10 distribution=22/2 p=0.0036
Core
E1
E2
p7
NS2
NS3protease
NS3helicase
NS4A
NS4B
NS5A
NS5B
VPQVGSCAAL
VPQVGSCAAL
VPQVGSCAAL
VPQVGSCAAL
VPQVGSCAAL
VPQVGSCAAL
VPQVGSCAAL
VPQVGSCAAL
VPQVGSCAAL
VPQVGSCAAL
VPQVGSCAAL
VPQVGSCAAL
VPQVGSCAAL
VPQVGSCAAL
VPQVGSCAAL
VPQVGSCAAL
VPQVGSCAAL
CRLAEAAGGF
CRLAEAAGGF
VPQVGSCAAL
VPQVGSCAAL
VPQVGSCAAL
VPQVGSCAAL
VPQVGSCAAL

HCV_2b_mafft BIS_dim1_cluster22_sym100_env100 #hits=2 distribution=20/4 p=9.4e−05

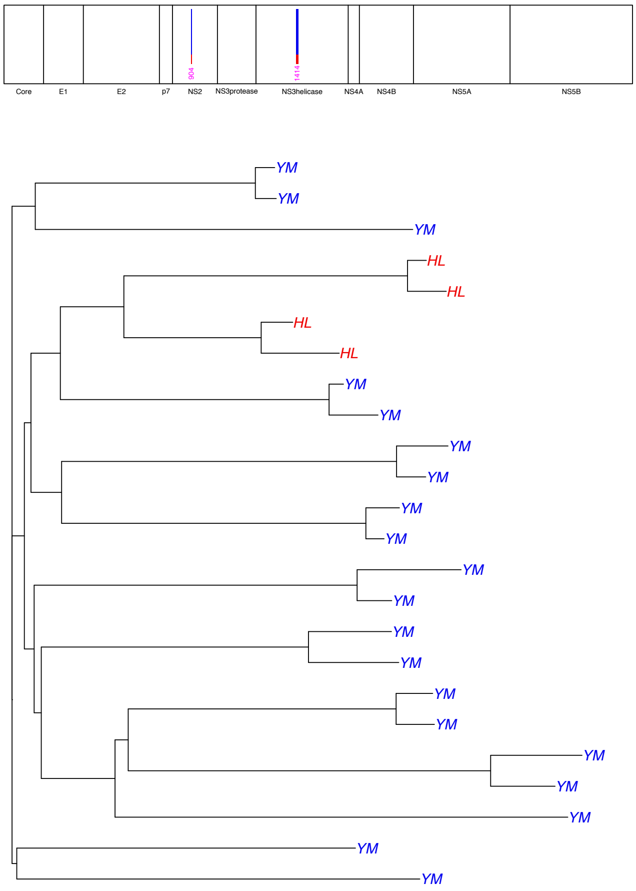

HCV_2b_mafft BIS_dim1_cluster24_sym100_env100 #hits=2 distribution=20/4 p=9.4e−05

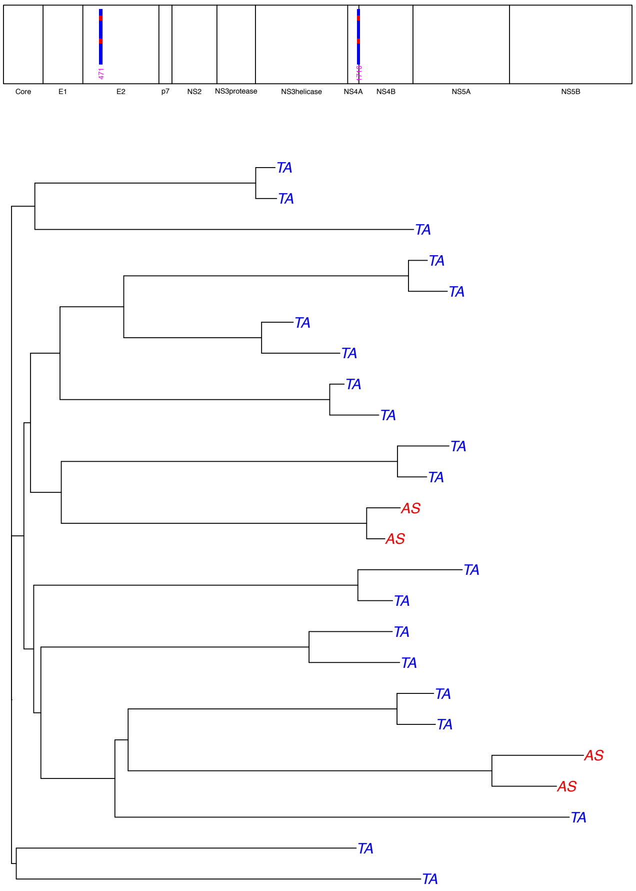

HCV_2b_mafft BIS_dim1_cluster26_sym100_env100 #hits=2 distribution=22/2 p=0.0036

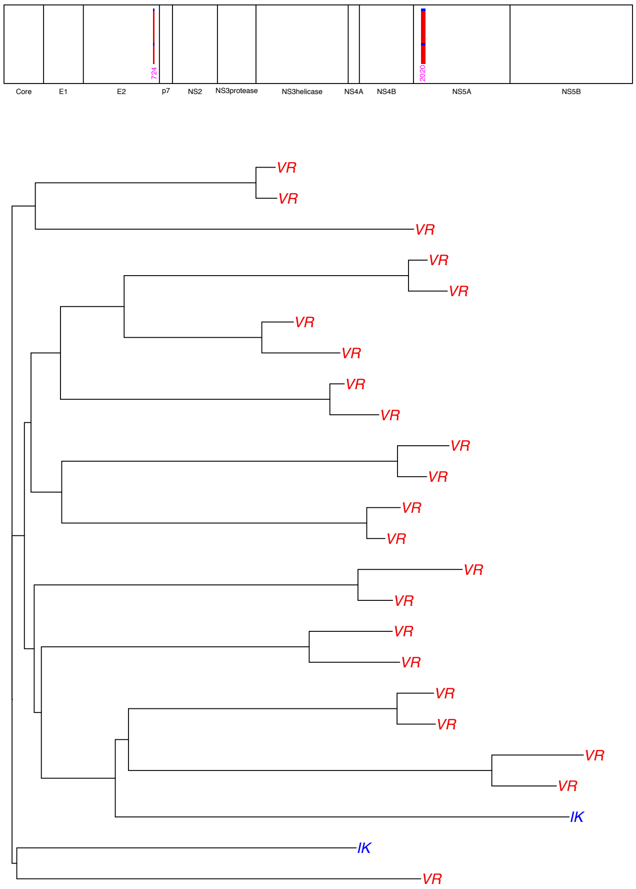

HCV_2b_mafft BIS_dim1_cluster29_sym100_env100 #hits=2 distribution=19/5 p=2.4e−05

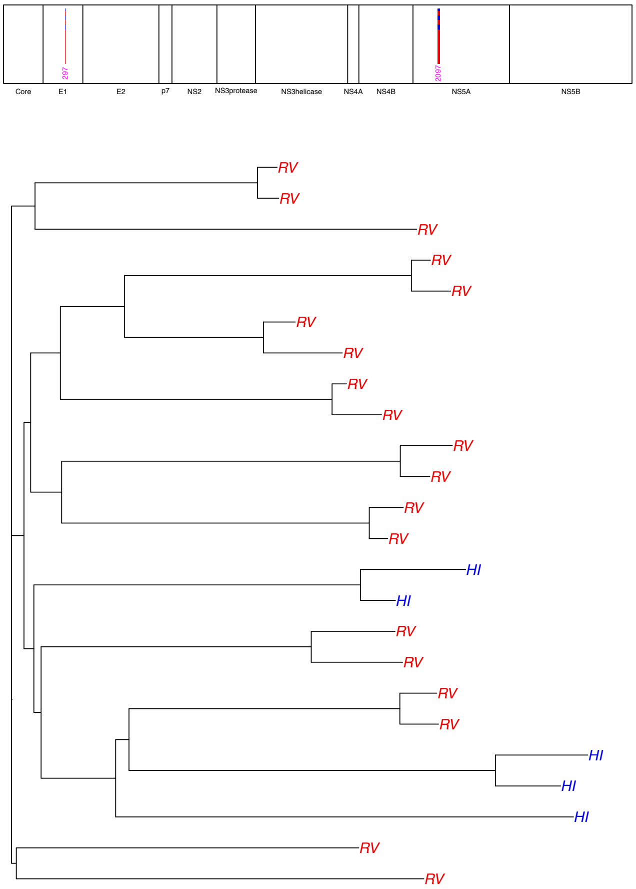

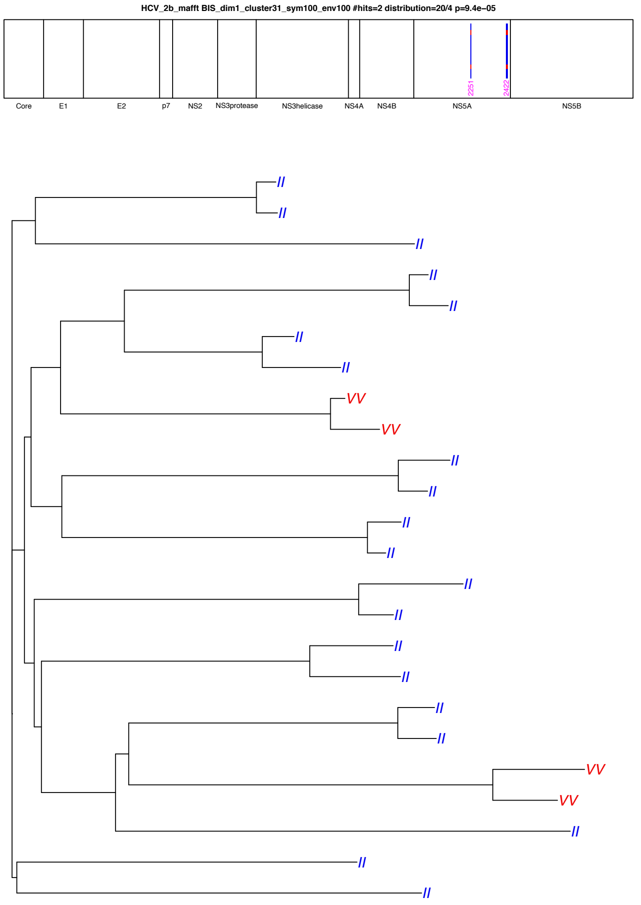
HCV_2b_mafft BIS_dim1_cluster31_sym100_env100 #hits=2 distribution=20/4 p=9.4e−05
2251
2422
Core
E1
E2
p7
NS2
NS3protease
NS3helicase
NS4A
NS4B
NS5A
NS5B
II
II
II
II
II
II
II
VV
VV
II
II
II
II
II
II
II
II
II
II
VV
VV
II
II
II

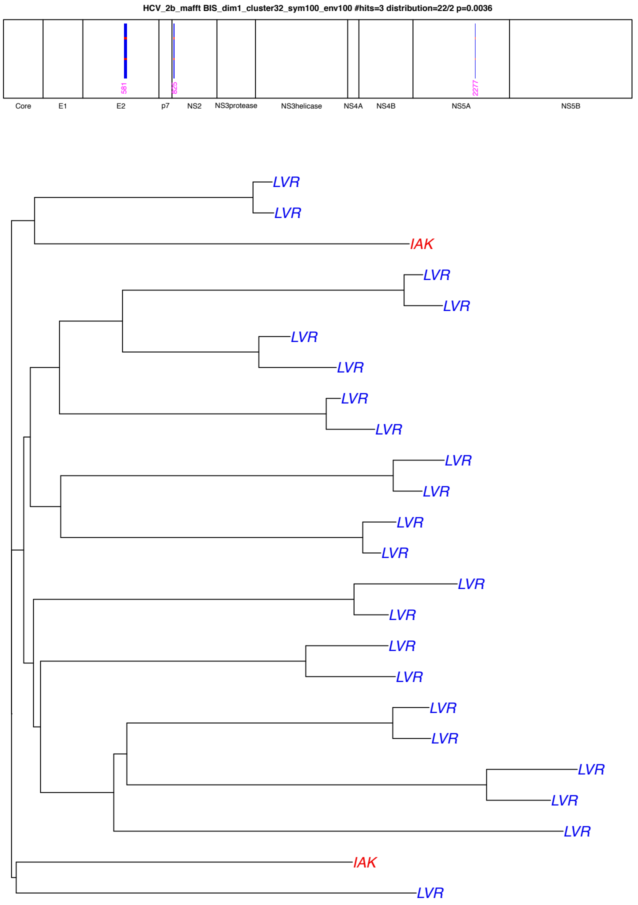
HCV_2b_mafft BIS_dim1_cluster32_sym100_env100 #hits=3 distribution=22/2 p=0.0036
581
825
2277
Core
E1
E2
p7
NS2
NS3protease
NS3helicase
NS4A
NS4B
NS5A
NS5B
LVR
LVR
IAK
LVR
LVR
LVR
LVR
LVR
LVR
LVR
LVR
LVR
LVR
LVR
LVR
LVR
LVR
LVR
LVR
LVR
LVR
LVR
IAK
LVR

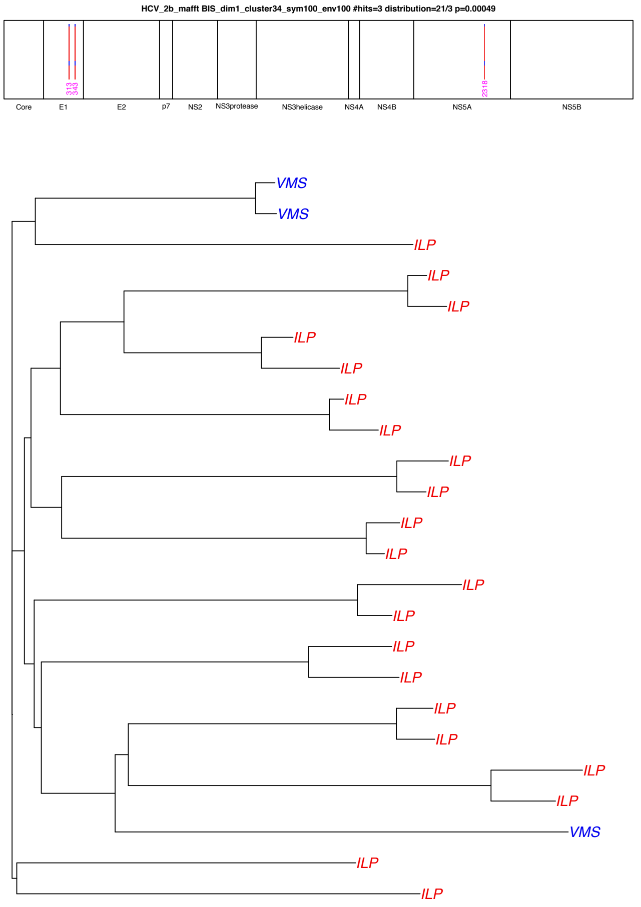
HCV_2b_mafft BIS_dim1_cluster34_sym100_env100 #hits=3 distribution=21/3 p=0.00049
313
343
2318
Core
E1
E2
p7
NS2
NS3protease
NS3helicase
NS4A
NS4B
NS5A
NS5B
VMS
VMS
ILP
ILP
ILP
ILP
ILP
ILP
ILP
ILP
ILP
ILP
ILP
ILP
ILP
ILP
ILP
ILP
ILP
ILP
ILP
ILP
VMS
ILP
ILP

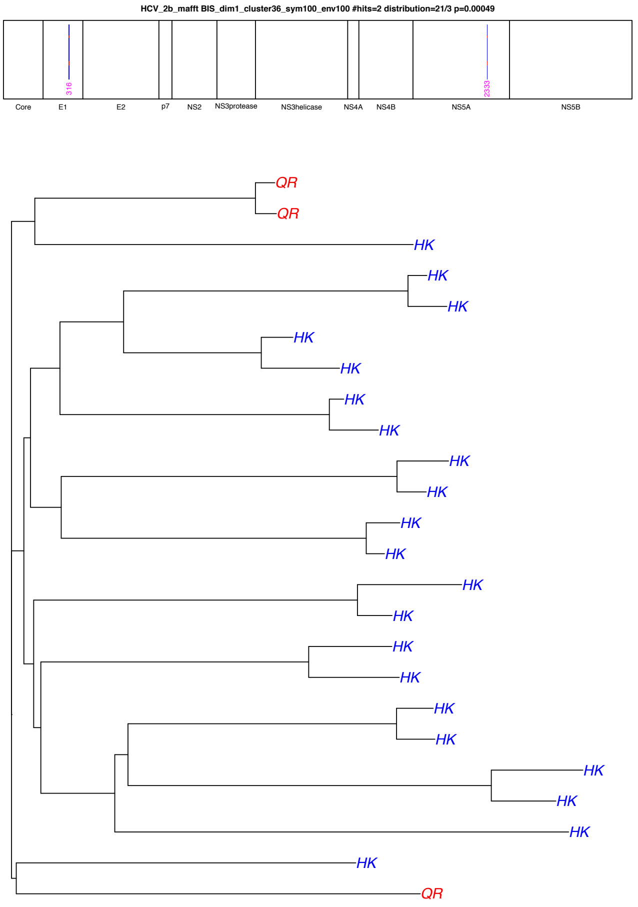
HCV_2b_mafft BIS_dim1_cluster36_sym100_env100 #hits=2 distribution=21/3 p=0.00049
316
2333
Core
E1
E2
p7
NS2
NS3protease
NS3helicase
NS4A
NS4B
NS5A
NS5B
QR
QR
HK
HK
HK
HK
HK
HK
HK
HK
HK
HK
HK
HK
HK
HK
HK
HK
HK
HK
HK
HK
HK
HK
QR

HCV_2b_mafft BIS_dim1_cluster37_sym100_env100 #hits=3 distribution=20/4 p=9.4e−05

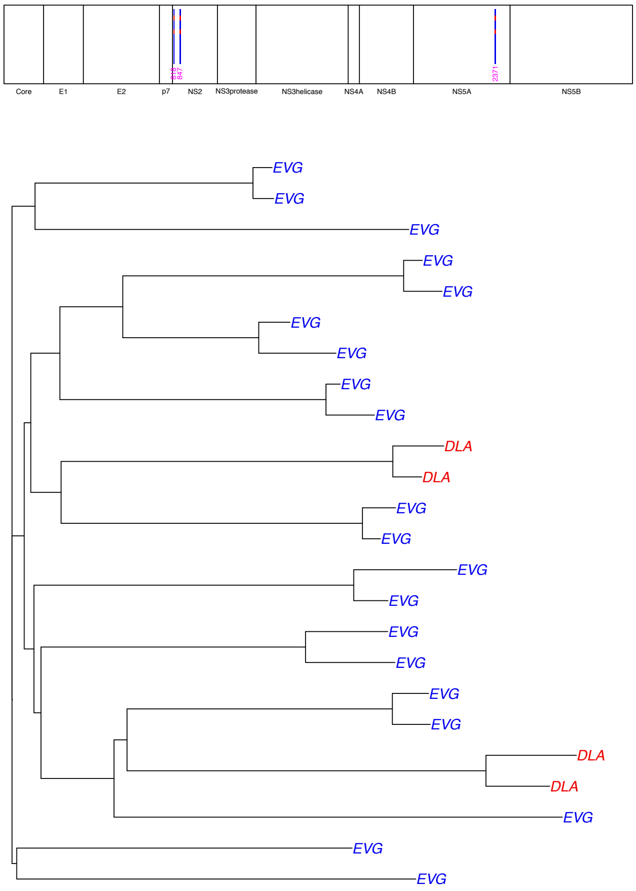

HCV_2b_mafft BIS_dim1_cluster38_sym100_env100 #hits=2 distribution=20/4 p=9.4e−05

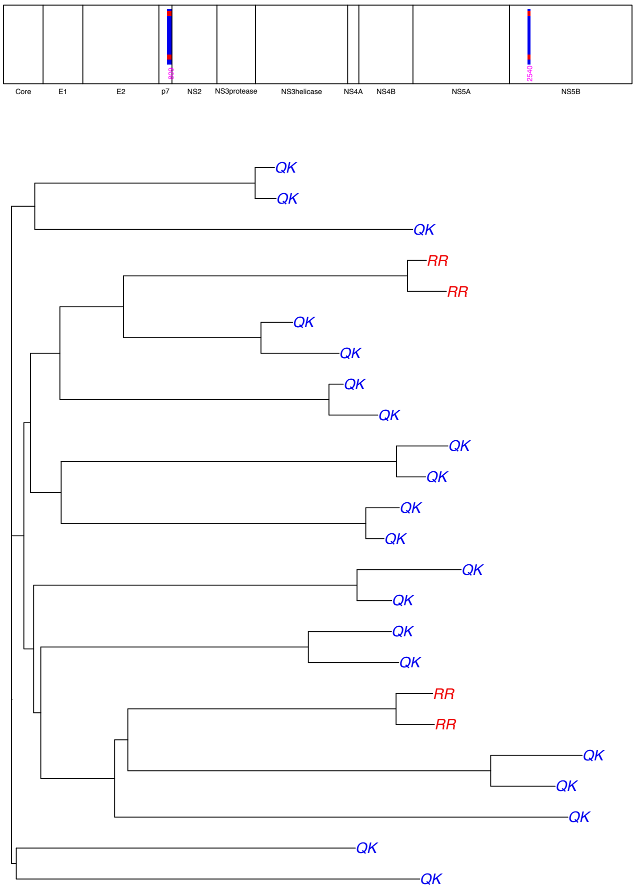

HCV_2b_mafft BIS_dim1_cluster39_sym100_env100 #hits=2 distribution=20/4 p=9.4e−05

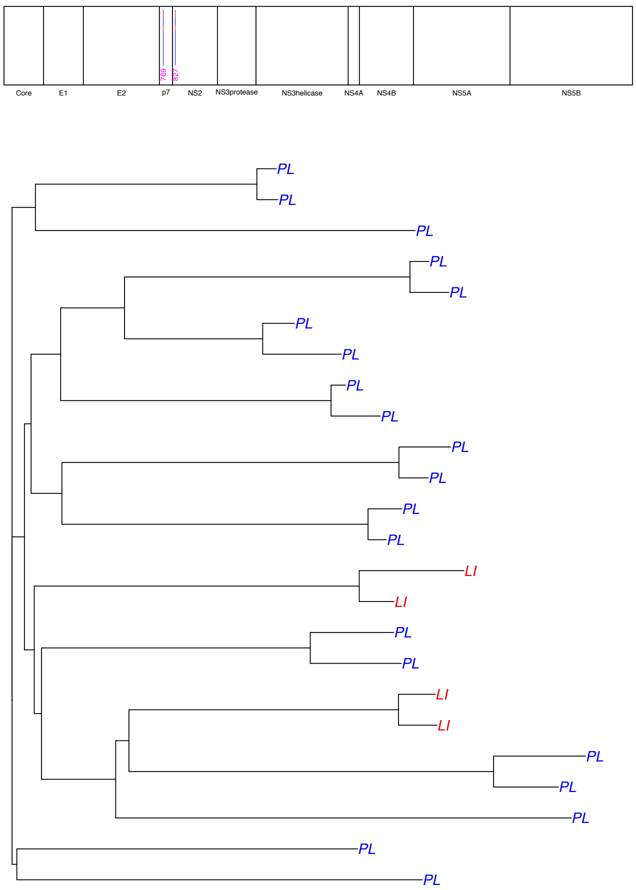

HCV_4_nodup_mafft BIS_dim1_cluster3_sym100_env100 #hits=6 distribution=24/3 p=0.00034

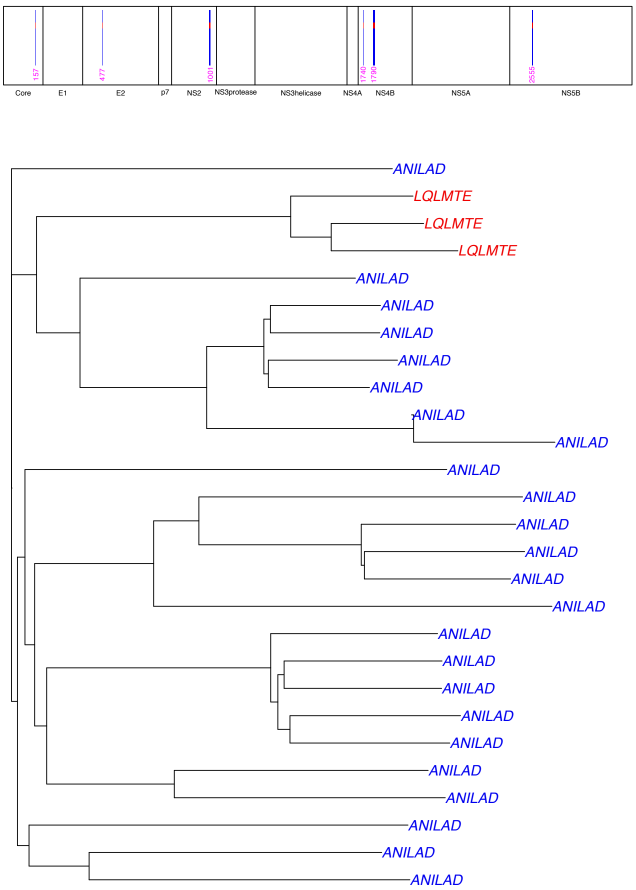

HCV_4_nodup_mafft BIS_dim1_cluster4_sym100_env100 #hits=2 distribution=25/2 p=0.0028

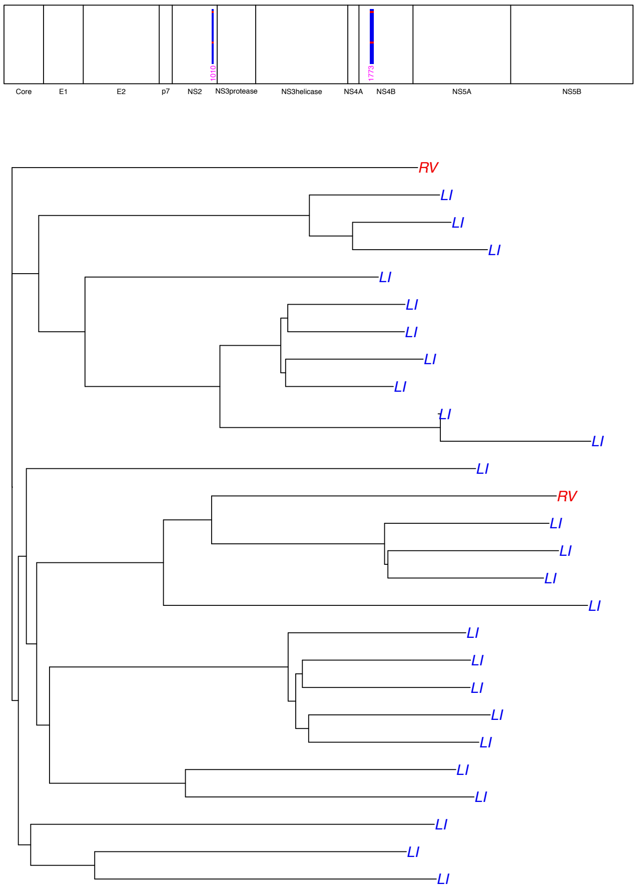

HCV_4_nodup_mafft BIS_dim1_cluster6_sym100_env100 #hits=2 distribution=23/3/1 p=1.4e−05

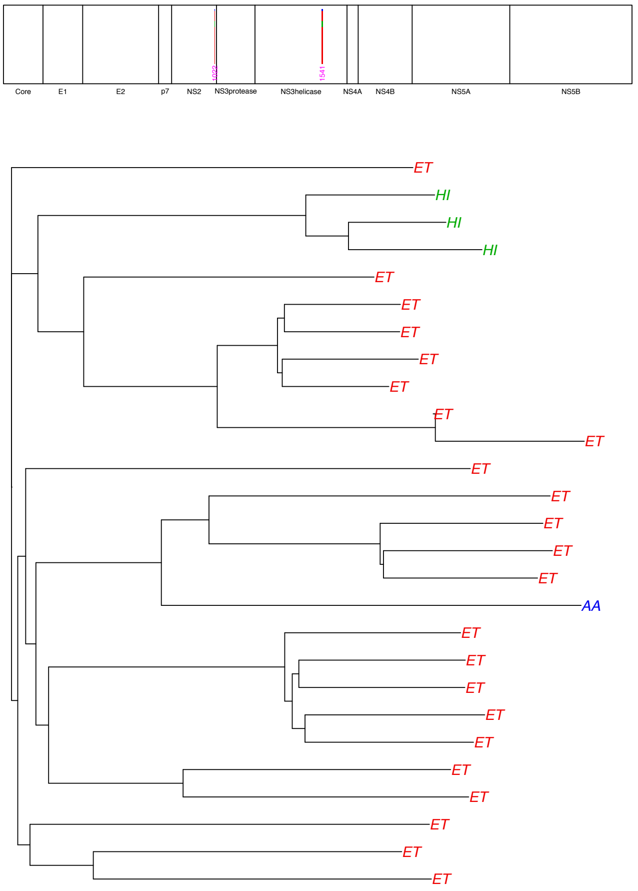

HCV_4_nodup_mafft BIS_dim1_cluster8_sym100_env100 #hits=9 distribution=22/5 p=1.2e−05

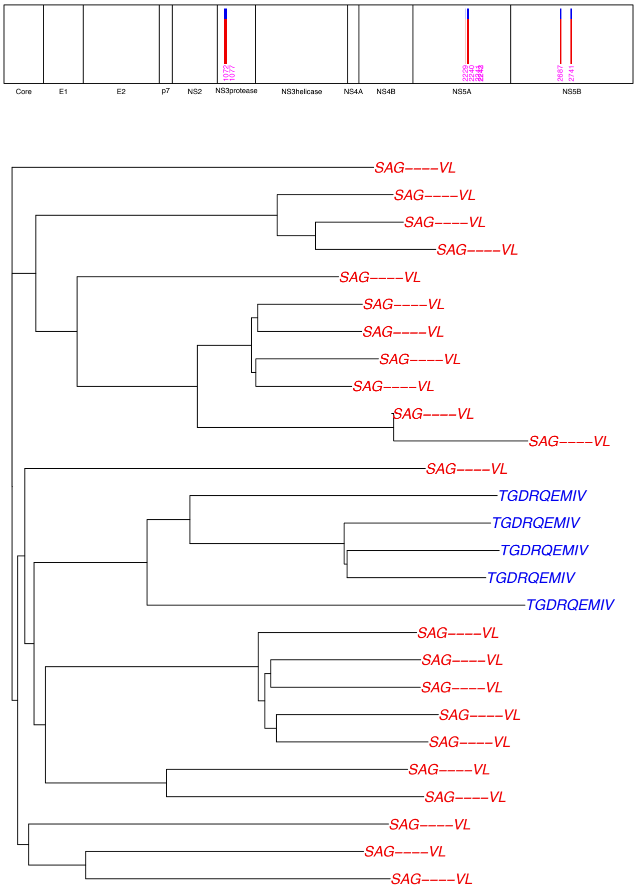

HCV_4_nodup_mafft BIS_dim1_cluster11_sym100_env100 #hits=2 distribution=25/2 p=0.0028

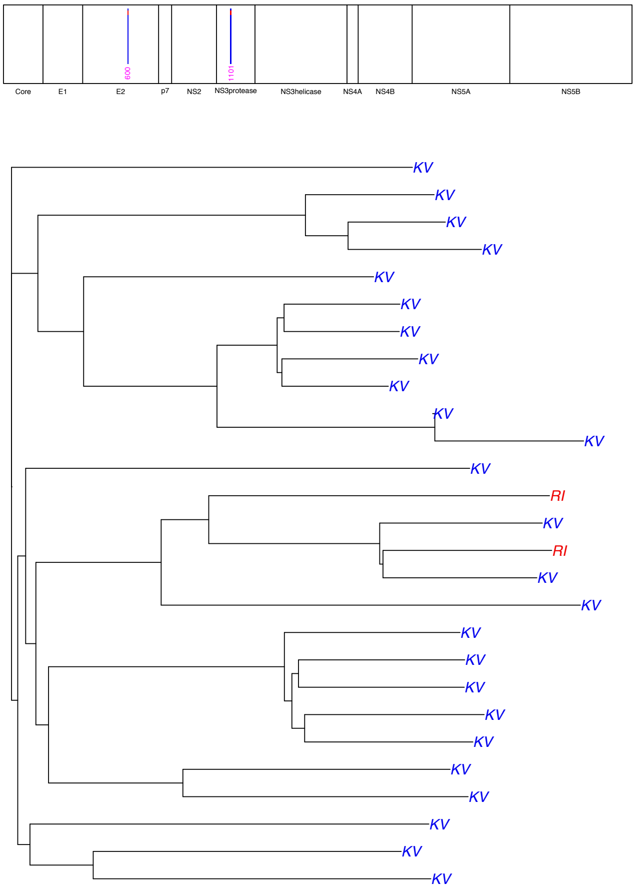

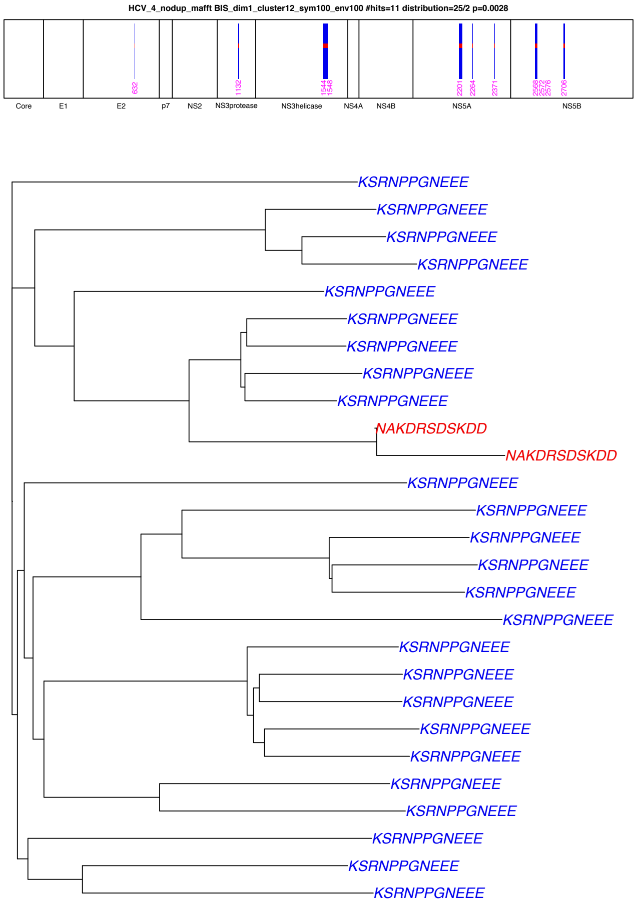
HCV_4_nodup_mafft BIS_dim1_cluster12_sym100_env100 #hits=11 distribution=25/2 p=0.0028
Core
E1
E2
p7
NS2
NS3protease
NS3helicase
NS4A
NS4B
NS5A
NS5B
KSRNPPGNEEE
KSRNPPGNEEE
KSRNPPGNEEE
KSRNPPGNEEE
KSRNPPGNEEE
KSRNPPGNEEE
KSRNPPGNEEE
KSRNPPGNEEE
KSRNPPGNEEE
NAKDRSDSKDD
NAKDRSDSKDD
KSRNPPGNEEE
KSRNPPGNEEE
KSRNPPGNEEE
KSRNPPGNEEE
KSRNPPGNEEE
KSRNPPGNEEE
KSRNPPGNEEE
KSRNPPGNEEE
KSRNPPGNEEE
KSRNPPGNEEE
KSRNPPGNEEE
KSRNPPGNEEE
KSRNPPGNEEE
KSRNPPGNEEE
KSRNPPGNEEE
KSRNPPGNEEE

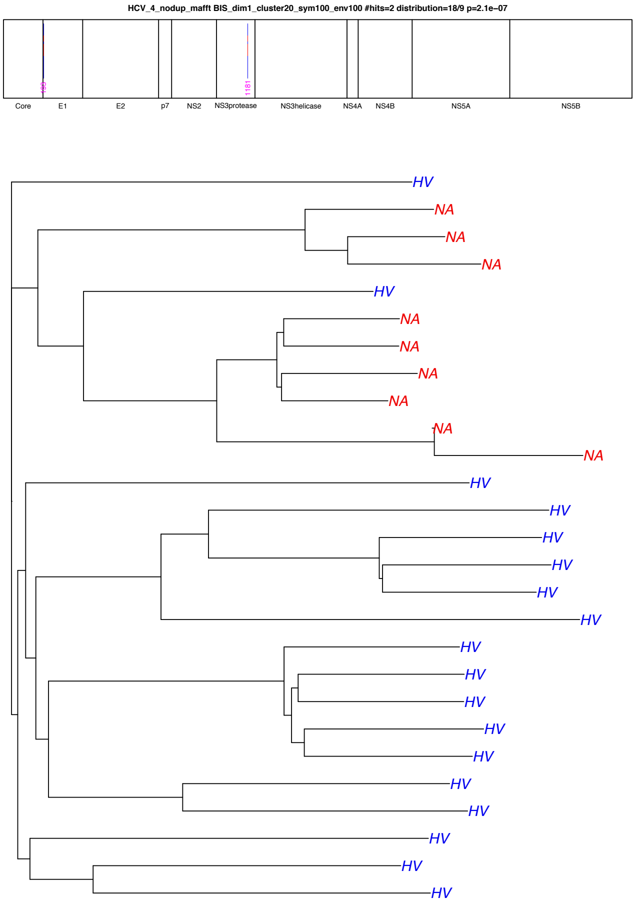
HCV_4_nodup_mafft BIS_dim1_cluster20_sym100_env100 #hits=2 distribution=18/9 p=2.1e−07
Core
E1
E2
p7
NS2
NS3protease
NS3helicase
NS4A
NS4B
NS5A
NS5B
HV
NA
NA
NA
HV
NA
NA
NA
NA
NA
NA
HV
HV
HV
HV
HV
HV
HV
HV
HV
HV
HV
HV
HV
HV
HV
HV

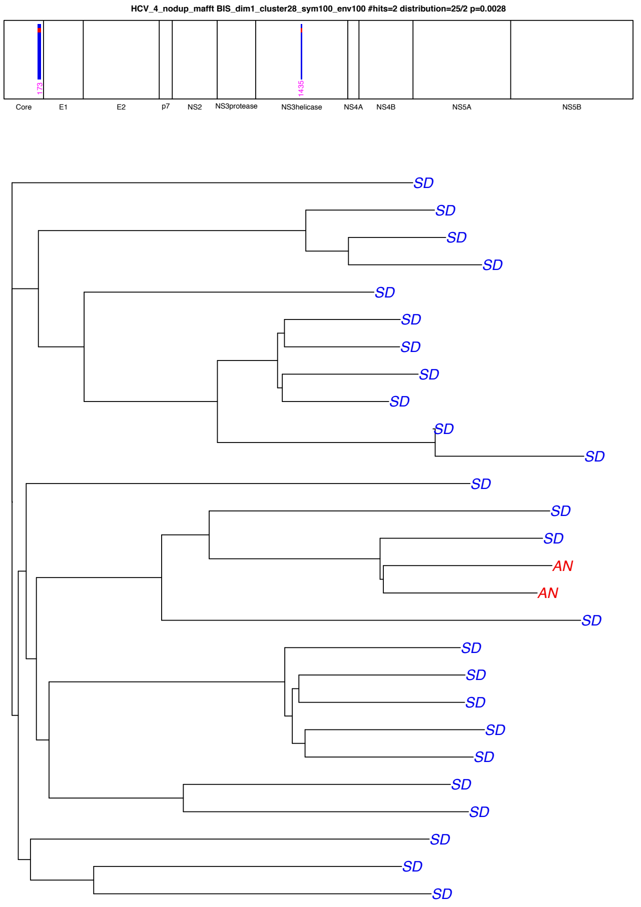
HCV_4_nodup_mafft BIS_dim1_cluster28_sym100_env100 #hits=2 distribution=25/2 p=0.0028
Core
E1
E2
p7
NS2
NS3protease
NS3helicase
NS4A
NS4B
NS5A
NS5B
SD
SD
SD
SD
SD
SD
SD
SD
SD
SD
SD
SD
SD
SD
AN
AN
SD
SD
SD
SD
SD
SD
SD
SD
SD
SD
SD

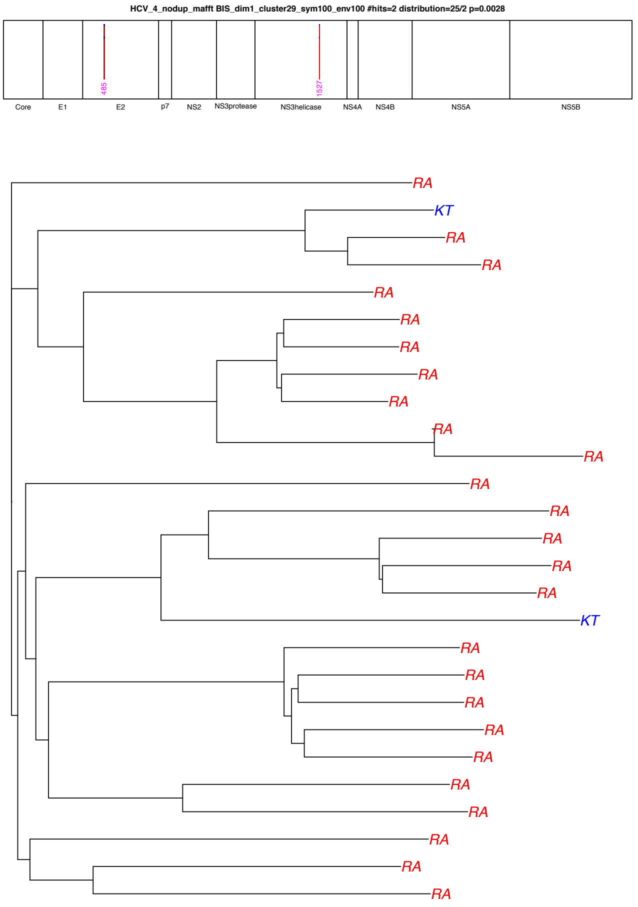
HCV_4_nodup_mafft BIS_dim1_cluster29_sym100_env100 #hits=2 distribution=25/2 p=0.0028
Core
E1
E2
p7
NS2
NS3protease
NS3helicase
NS4A
NS4B
NS5A
NS5B
RA
KT
RA
RA
RA
RA
RA
RA
RA
RA
RA
RA
RA
RA
RA
RA
KT
RA
RA
RA
RA
RA
RA
RA
RA
RA
RA

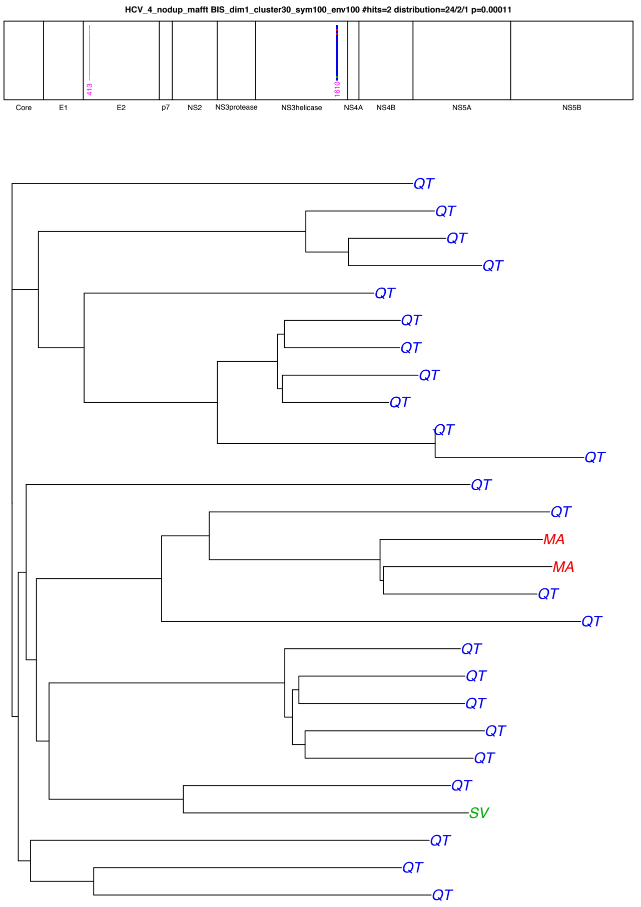
HCV_4_nodup_mafft BIS_dim1_cluster30_sym100_env100 #hits=2 distribution=24/2/1 p=0.00011
413
1610
Core
E1
E2
p7
NS2
NS3protease
NS3helicase
NS4A
NS4B
NS5A
NS5B
QT
QT
QT
QT
QT
QT
QT
QT
QT
QT
QT
QT
QT
MA
MA
QT
QT
QT
QT
QT
QT
QT
QT
SV
QT
QT
QT

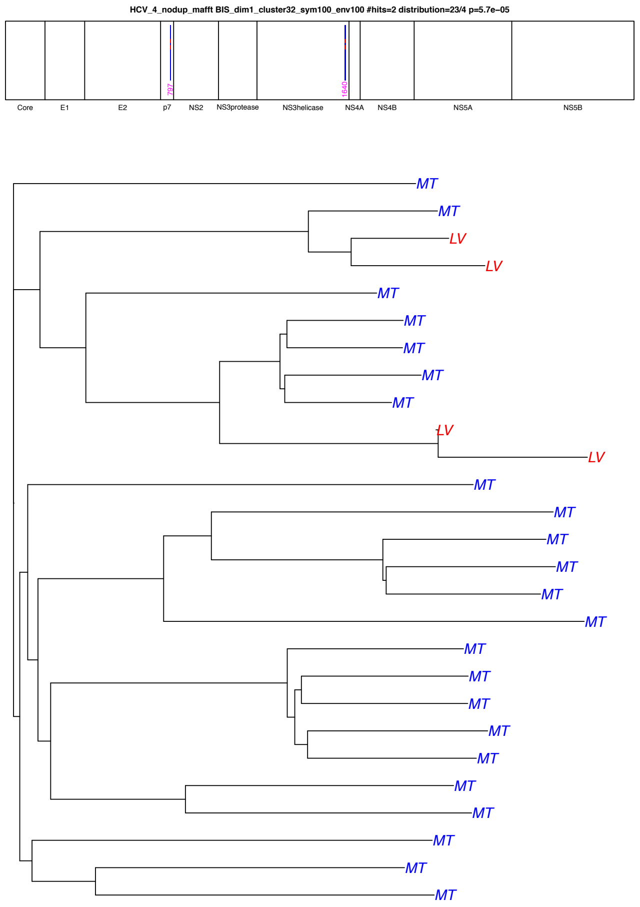
HCV_4_nodup_mafft BIS_dim1_cluster32_sym100_env100 #hits=2 distribution=23/4 p=5.7e−05
797
1640
Core
E1
E2
p7
NS2
NS3protease
NS3helicase
NS4A
NS4B
NS5A
NS5B
MT
MT
LV
LV
MT
MT
MT
MT
MT
LV
LV
MT
MT
MT
MT
MT
MT
MT
MT
MT
MT
MT
MT
MT
MT
MT
MT

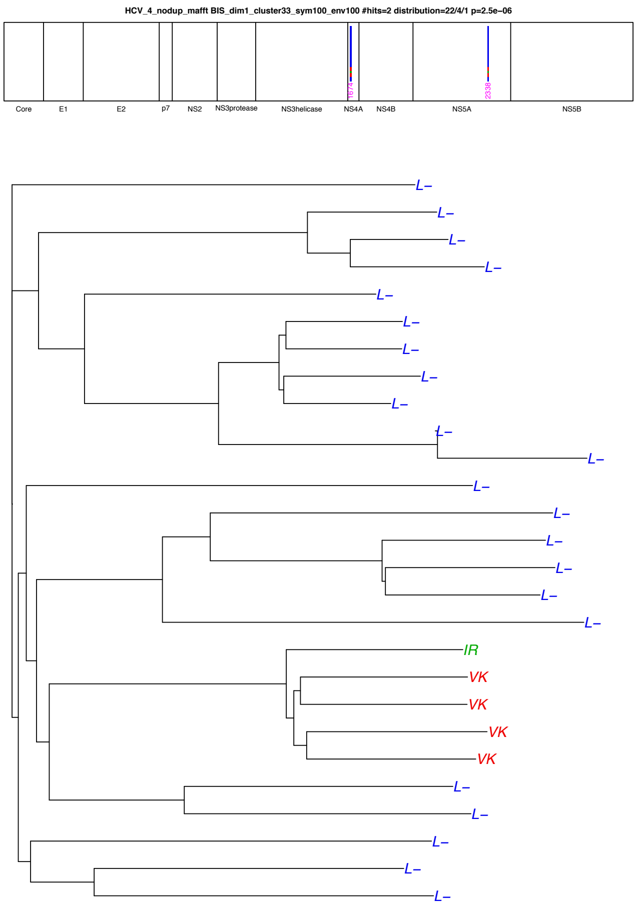
HCV_4_nodup_mafft BIS_dim1_cluster33_sym100_env100 #hits=2 distribution=22/4/1 p=2.5e−06
1674
2338
Core
E1
E2
p7
NS2
NS3protease
NS3helicase
NS4A
NS4B
NS5A
NS5B
L–
L–
L–
L–
L–
L–
L–
L–
L–
L–
L–
L–
L–
L–
L–
L–
L–
IR
VK
VK
VK
VK
L–
L–
L–
L–
L–

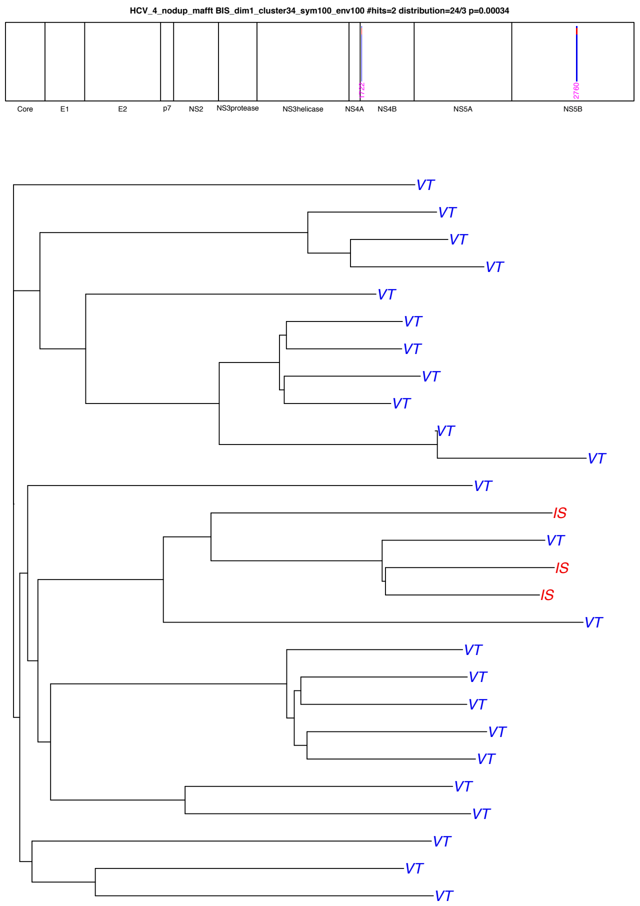
HCV_4_nodup_mafft BIS_dim1_cluster34_sym100_env100 #hits=2 distribution=24/3 p=0.00034
1722
2760
Core
E1
E2
p7
NS2
NS3protease
NS3helicase
NS4A
NS4B
NS5A
NS5B
VT
VT
VT
VT
VT
VT
VT
VT
VT
VT
VT
VT
IS
VT
IS
IS
VT
VT
VT
VT
VT
VT
VT
VT
VT
VT
VT

HCV_4_nodup_mafft BIS_dim1_cluster35_sym100_env100 #hits=3 distribution=20/7 p=1.1e−06

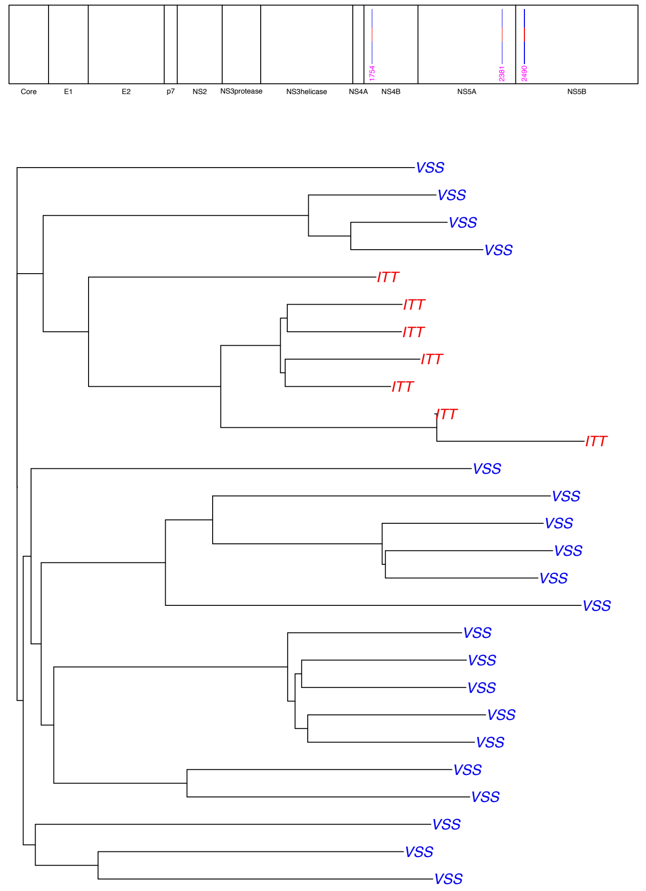

HCV_4_nodup_mafft BIS_dim1_cluster38_sym100_env100 #hits=8 distribution=24/3 p=0.00034

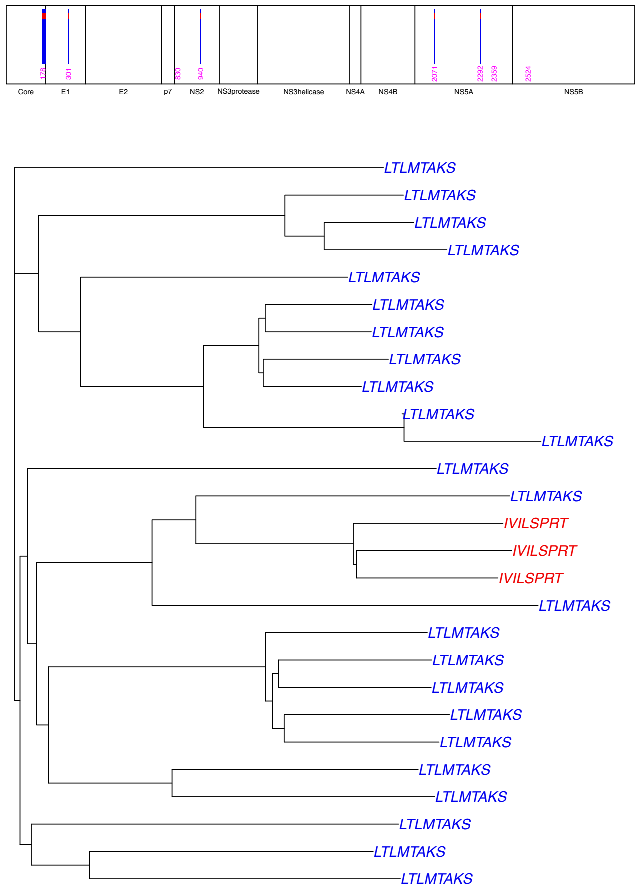

HCV_4_nodup_mafft BIS_dim1_cluster39_sym100_env100 #hits=8 distribution=22/5 p=1.2e−05

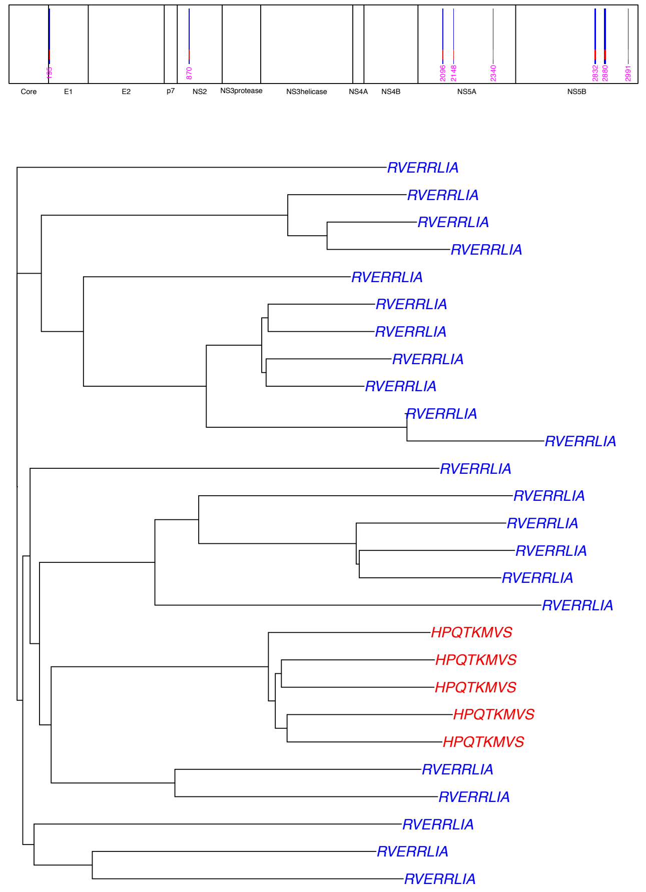

HCV_4_nodup_mafft BIS_dim1_cluster40_sym100_env100 #hits=2 distribution=24/3 p=0.00034

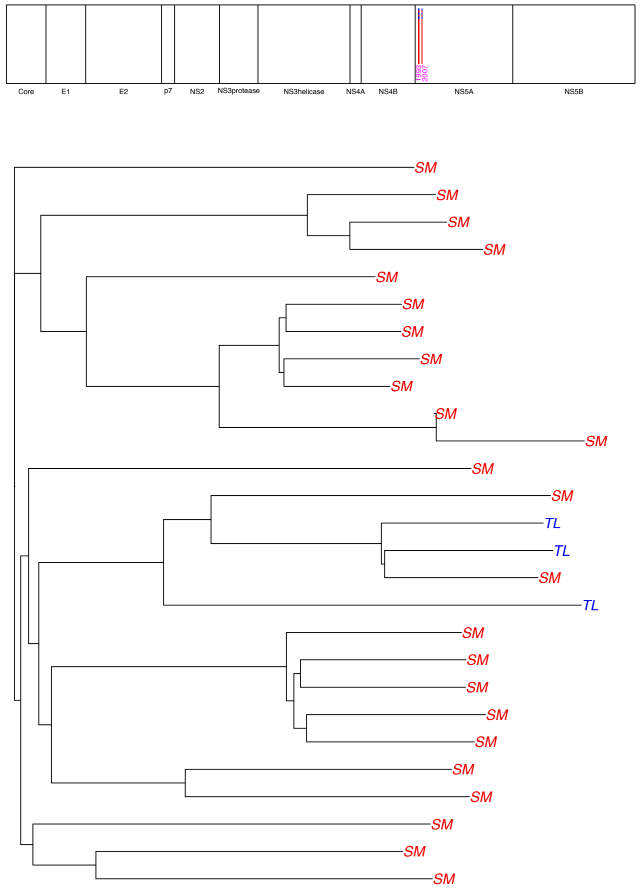

HCV_4_nodup_mafft BIS_dim1_cluster41_sym100_env100 #hits=2 distribution=25/2 p=0.0028

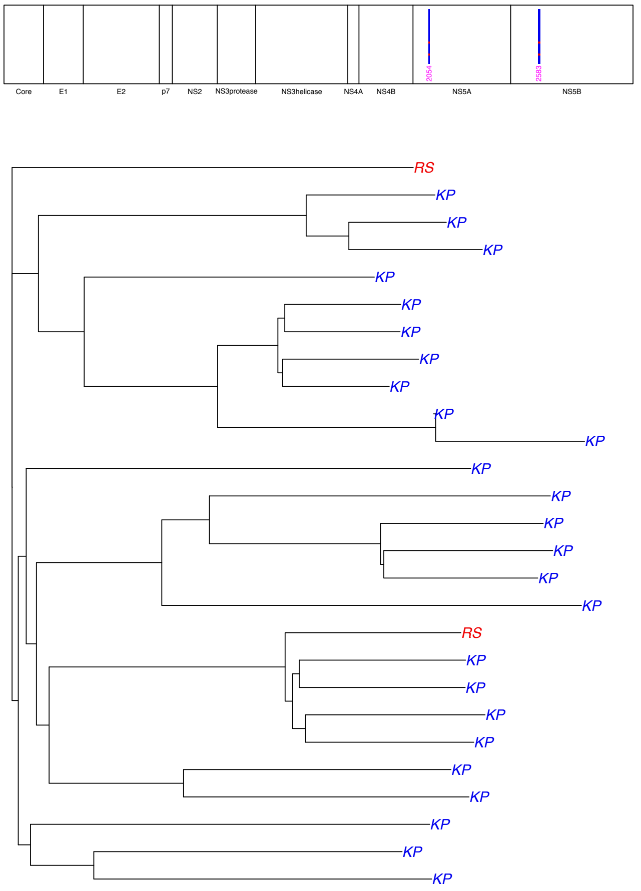

HCV_4_nodup_mafft BIS_dim1_cluster42_sym100_env100 #hits=2 distribution=25/2 p=0.0028

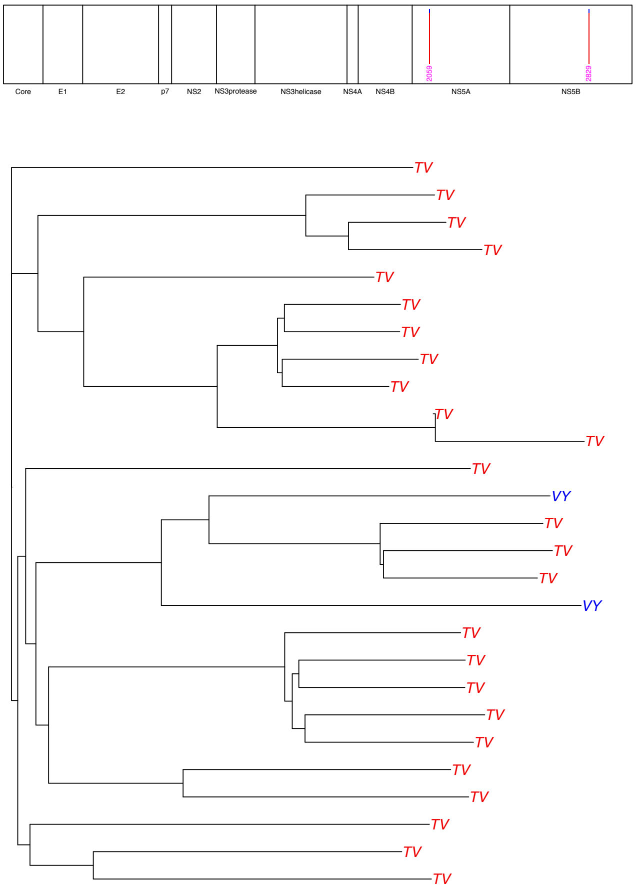

HCV_4_nodup_mafft BIS_dim1_cluster43_sym100_env100 #hits=3 distribution=25/2 p=0.0028

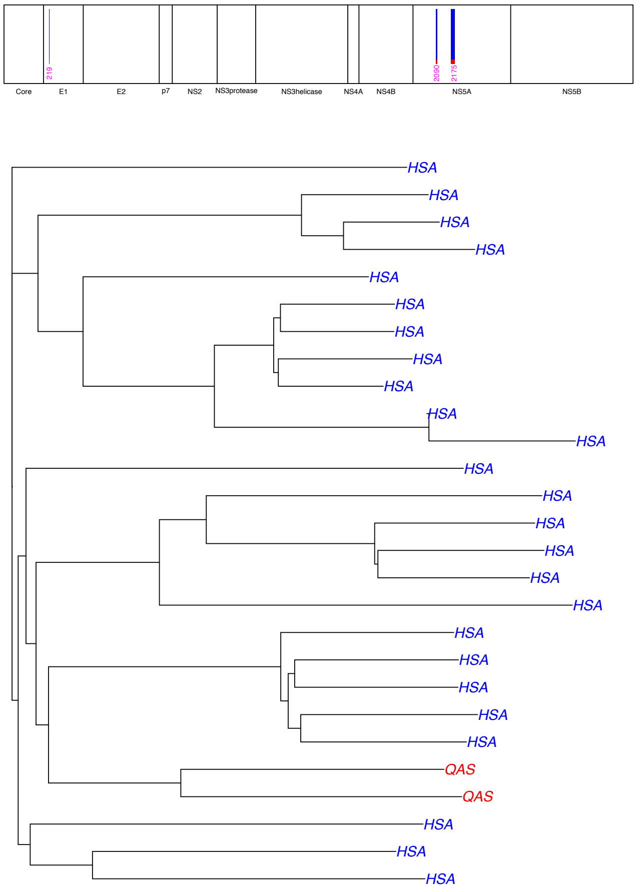

HCV_4_nodup_mafft BIS_dim1_cluster44_sym100_env100 #hits=3 distribution=23/4 p=5.7e−05

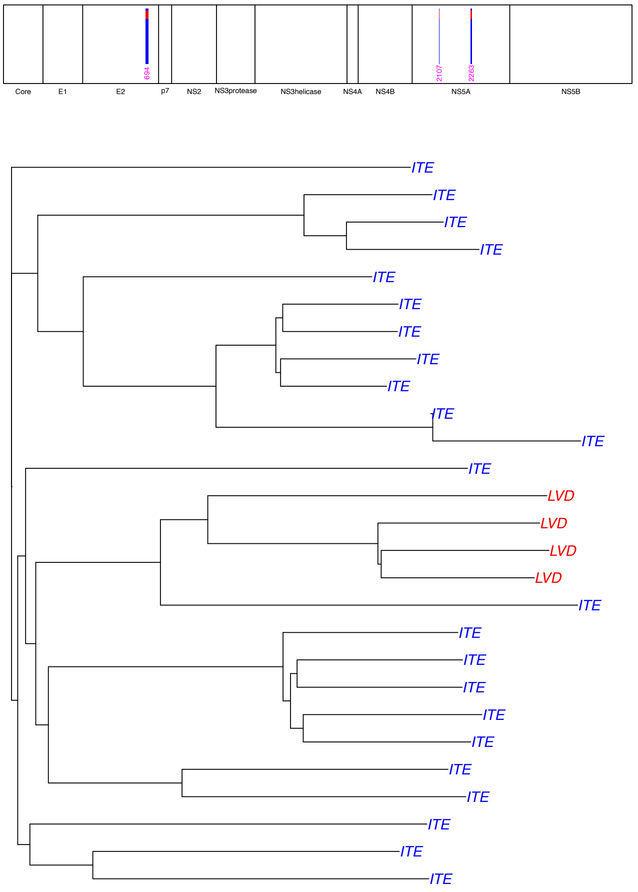

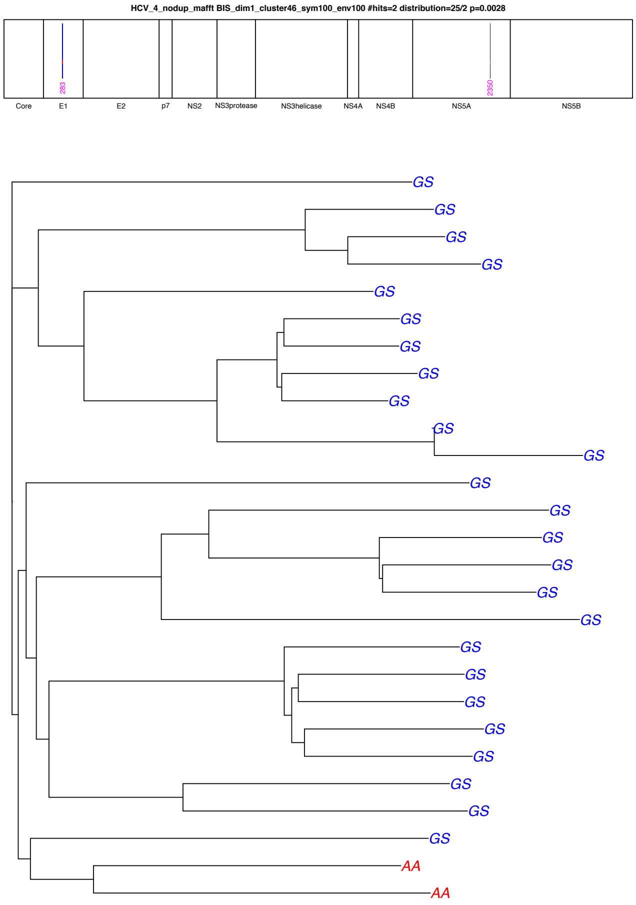
HCV_4_nodup_mafft BIS_dim1_cluster46_sym100_env100 #hits=2 distribution=25/2 p=0.0028
283
2350
Core
E1
E2
p7
NS2
NS3protease
NS3helicase
NS4A
NS4B
NS5A
NS5B
GS
AA

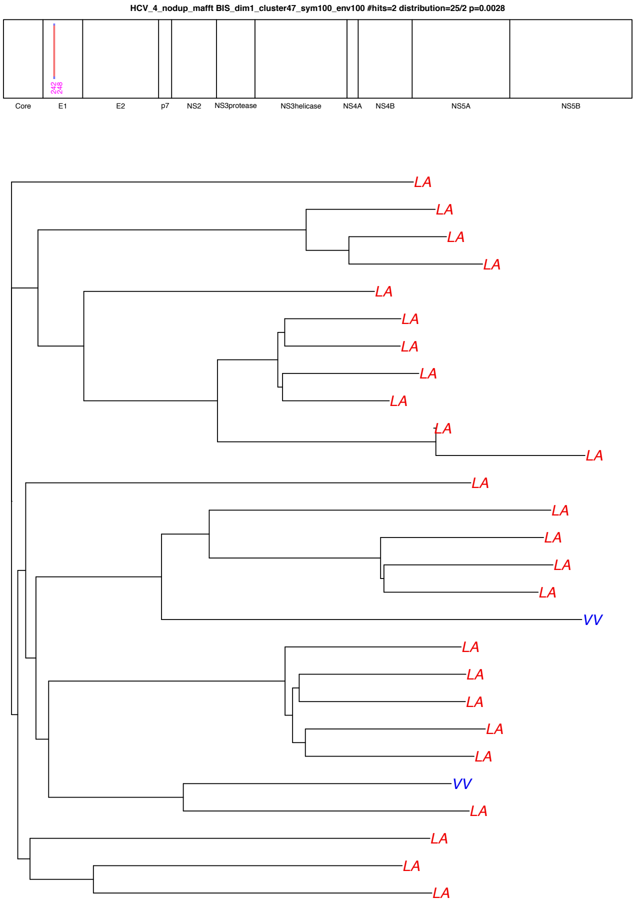
HCV_4_nodup_mafft BIS_dim1_cluster47_sym100_env100 #hits=2 distribution=25/2 p=0.0028
242
248
Core
E1
E2
p7
NS2
NS3protease
NS3helicase
NS4A
NS4B
NS5A
NS5B
LA
VV

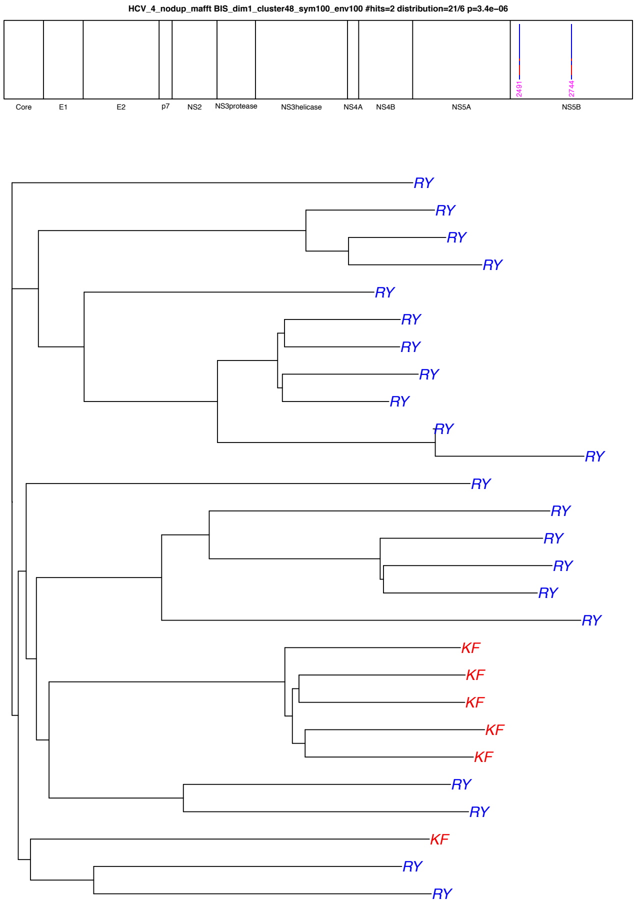
HCV_4_nodup_mafft BIS_dim1_cluster48_sym100_env100 #hits=2 distribution=21/6 p=3.4e−06
2491
2744
Core
E1
E2
p7
NS2
NS3protease
NS3helicase
NS4A
NS4B
NS5A
NS5B
RY
KF

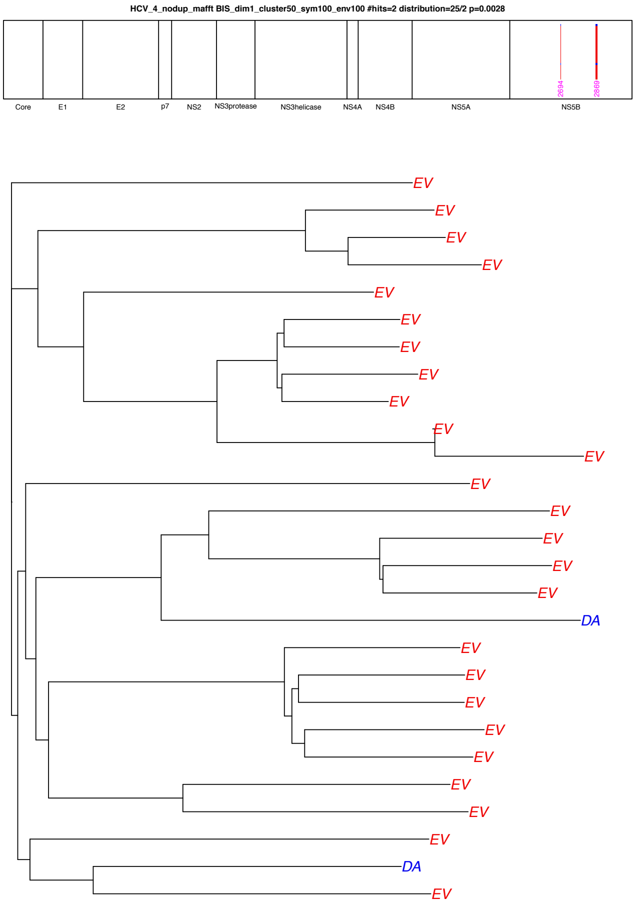
HCV_4_nodup_mafft BIS_dim1_cluster50_sym100_env100 #hits=2 distribution=25/2 p=0.0028
2694
2869
Core
E1
E2
p7
NS2
NS3protease
NS3helicase
NS4A
NS4B
NS5A
NS5B
EV
DA

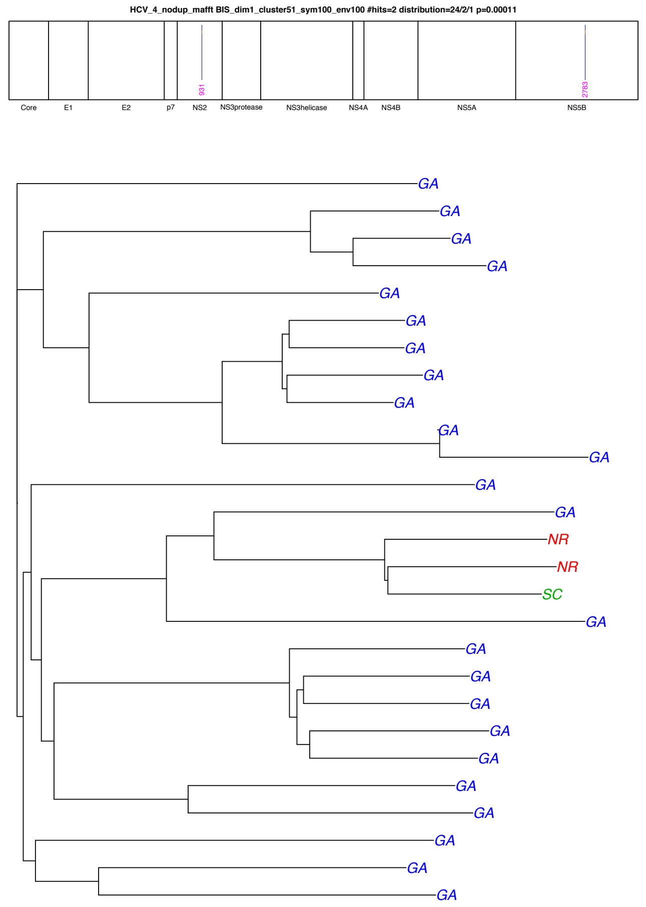
HCV_4_nodup_mafft BIS_dim1_cluster51_sym100_env100 #hits=2 distribution=24/2/1 p=0.00011
931
2783
Core
E1
E2
p7
NS2
NS3protease
NS3helicase
NS4A
NS4B
NS5A
NS5B
GA
GA
GA
GA
GA
GA
GA
GA
GA
GA
GA
GA
GA
NR
NR
SC
GA
GA
GA
GA
GA
GA
GA
GA
GA
GA
GA

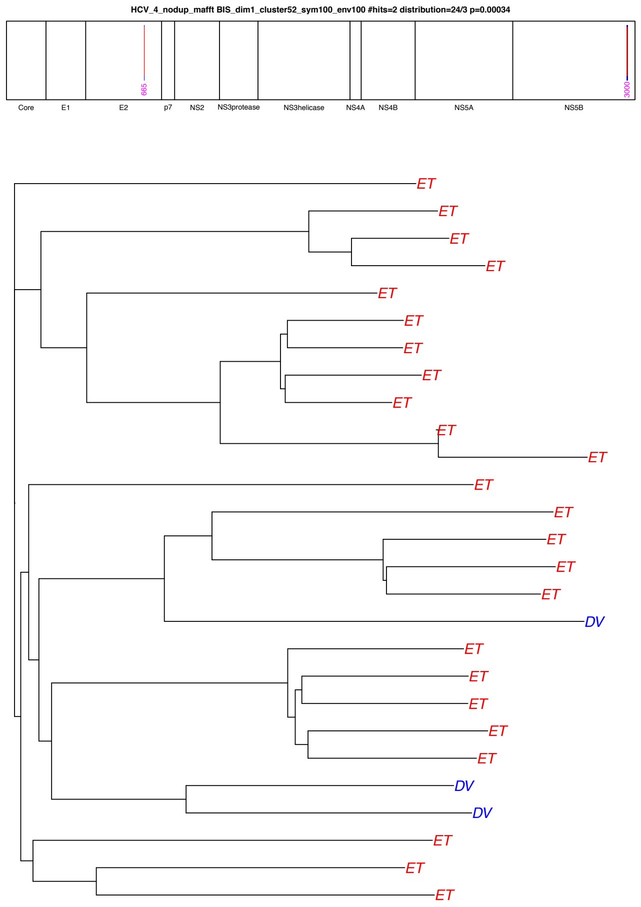
HCV_4_nodup_mafft BIS_dim1_cluster52_sym100_env100 #hits=2 distribution=24/3 p=0.00034
665
3000
Core
E1
E2
p7
NS2
NS3protease
NS3helicase
NS4A
NS4B
NS5A
NS5B
ET
ET
ET
ET
ET
ET
ET
ET
ET
ET
ET
ET
ET
ET
ET
ET
DV
ET
ET
ET
ET
ET
DV
DV
ET
ET
ET

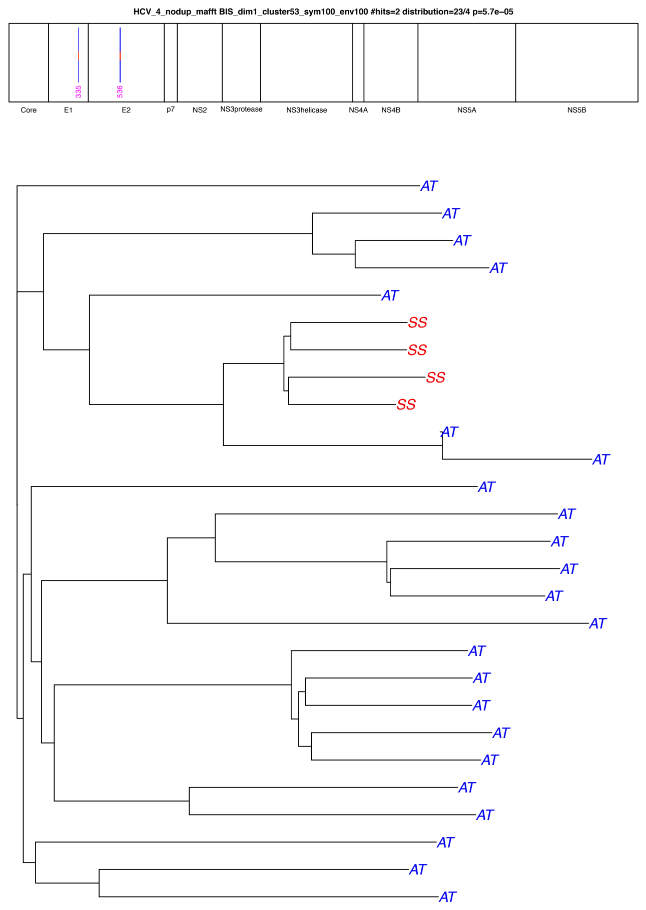
HCV_4_nodup_mafft BIS_dim1_cluster53_sym100_env100 #hits=2 distribution=23/4 p=5.7e−05
335
536
Core
E1
E2
p7
NS2
NS3protease
NS3helicase
NS4A
NS4B
NS5A
NS5B
AT
AT
AT
AT
AT
SS
SS
SS
SS
AT
AT
AT
AT
AT
AT
AT
AT
AT
AT
AT
AT
AT
AT
AT
AT
AT
AT

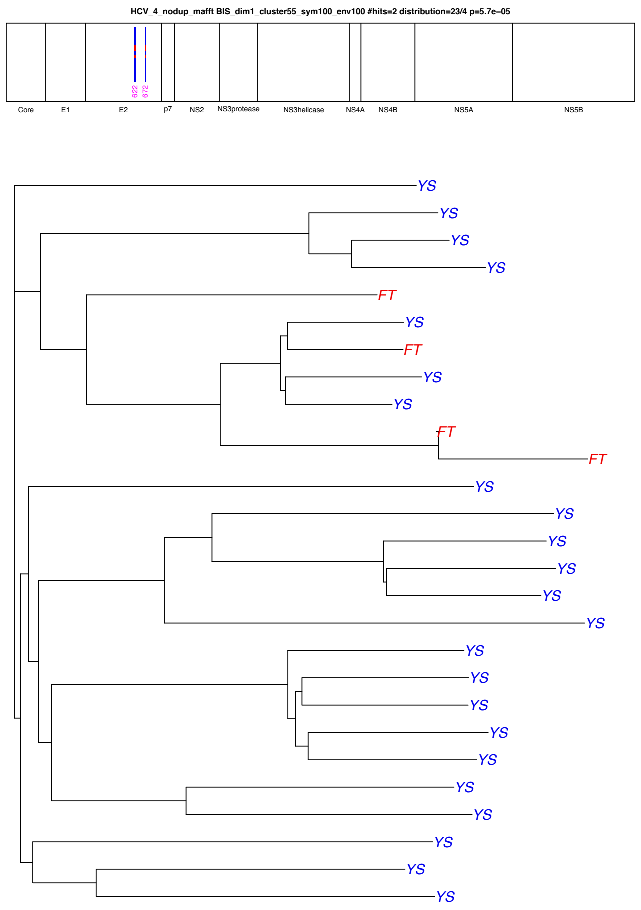
HCV_4_nodup_mafft BIS_dim1_cluster55_sym100_env100 #hits=2 distribution=23/4 p=5.7e−05
622
672
Core
E1
E2
p7
NS2
NS3protease
NS3helicase
NS4A
NS4B
NS5A
NS5B
YS
YS
YS
YS
FT
YS
FT
YS
YS
FT
FT
YS
YS
YS
YS
YS
YS
YS
YS
YS
YS
YS
YS
YS
YS
YS
YS
